# The Anti-Parkinsonian A2A Receptor Antagonist Istradefylline (KW-6002) Attenuates Behavioral Abnormalities, Neuroinflammation, and Neurodegeneration in Cerebral Ischemia: An Adenosinergic Signaling Link Between Stroke and Parkinson’s Disease

**DOI:** 10.3390/ijms26125680

**Published:** 2025-06-13

**Authors:** Michael G. Zaki, Elisabet Jakova, Mahboubeh Pordeli, Elina Setork, Changiz Taghibiglou, Francisco S. Cayabyab

**Affiliations:** 1 Department of Surgery, Neuroscience Research Cluster, College of Medicine, University of Saskatchewan, 107 Wiggins Road, Saskatoon, SK S7N 5E5, Canada; 2 Department of Anatomy, Physiology and Pharmacology, University of Saskatchewan, 107 Wiggins Road, Saskatoon, SK S7N 5E5, Canadachangiz.taghibiglou@usask.ca (C.T.)

**Keywords:** glutamate excitotoxicity, ischemic stroke, adenosine A2A receptor, adenosine A1 receptor, fEPSP, istradefylline, stroke model

## Abstract

Stroke, the third leading cause of death worldwide, is a major cause of functional disability. Cerebral ischemia causes a rapid elevation of adenosine, the main neuromodulator in the brain. The inhibition of adenosine A2A receptors (A2ARs) has been introduced as a potential target in neurodegenerative disorders involving extracellular adenosine elevation. Istradefylline, a selective A2AR antagonist, has been approved for Parkinson’s disease (PD) adjunctive therapy and showed neuroprotective effects in PD and Alzheimer’s disease. However, the role of A2ARs in post-stroke neuronal damage and behavioral deficits remains unclear. We recently showed that A2AR antagonism prevented the adenosine-induced post-hypoxia synaptic potentiation of glutamatergic neurotransmission following the hypoxia/reperfusion of hippocampal slices. Here, we investigated the potential neuroprotective effects of istradefylline in male *Sprague-Dawley* rats subjected to pial vessel disruption (PVD) used to model a small-vessel stroke. Rats were treated with either a vehicle control or istradefylline (3 mg/kg i.p.) following PVD surgery for three days. Istradefylline administration prevented anxiety and depressive-like behaviors caused by PVD stroke. In addition, istradefylline significantly attenuated ischemia-induced cognitive impairment and motor deficits. Moreover, istradefylline markedly reduced hippocampal neurodegeneration, as well as GFAP/Iba-1, TNF-α, nNOS, and iNOS levels after PVD, but prevented the downregulation of anti-inflammatory markers TGF-β1 and IL-4. Together, these results suggest a molecular link between stroke and PD and that the anti-PD drug istradefylline displays translational potential for drug repurposing as a neuroprotective agent for cerebral ischemic damage.

## 1. Introduction

Stroke is a medical emergency caused by a transient or permanent disruption in blood flow to a part of the brain and has become a major cause of death and long-term disability worldwide [1,2]. In 2020, stroke deaths accounted for 11% of total deaths worldwide, making stroke the second leading global cause of death behind ischemic heart diseases [3,4]. In fact, the recent global pandemic of severe acute respiratory syndrome coronavirus 2 (SARS-CoV-2), named as coronavirus disease in 2019 (COVID-19), increased both the risk and mortality of ischemic stroke [5]. Ischemic stroke is considered a cerebrovascular disorder that is involved in the pathogenesis of multiple neurodegenerative disorders [6], but whether there are shared molecular and biochemical pathways linking stroke and neurodegenerative diseases remains unclear. Parkinson’s disease (PD) is a neurodegenerative disorder that results from the neuronal loss of dopaminergic neurons in the basal ganglia with subsequent overactivity of cholinergic neurons, which leads to tremors, muscle rigidity, and motor hypokinesia as well as non-motor manifestations [7]. Both ischemic stroke and PD share many risk factors, including aging. A recent study showed that the ischemic stroke model in mice resulted in the overexpression of alpha-synuclein and Parkinsonism-like symptoms [8]. Interestingly, previous studies showed a higher risk of PD in patients who previously had an ischemic stroke, which suggested that PD may be associated with cerebrovascular disease [9,10]. In this study, we sought to investigate the potential connection between ischemic stroke and the motor deficits and non-motor symptoms that typically follow cerebral ischemia and explored the possible molecular mechanisms underlying the shared pathogenesis of neurodegeneration occurring in ischemic stroke and PD.

Cerebral ischemia leads to rapid elevation in extracellular adenosine concentrations due to the breakdown of extracellularly released ATP and the selective downregulation of concentrative nucleoside transporter 2 (CNT2); however, this adenosine elevation did not appear to depend on either CNT3 or equilibrative nucleoside transporter-1 (ENT-1), suggesting that this adaptive nucleoside transporter response contributes to the extracellular accumulation of adenosine during ischemia [11,12,13]. It is well known that the hypoxia-induced elevation of adenosine causes CNS preconditioning and provides neuroprotective effects for the neighboring ischemic penumbra, mainly through the inhibitory, Gαi-coupled adenosine A1 receptors (A1Rs), resulting in a reduction in synaptic transmission and glutamate excitotoxicity [14,15,16,17,18]. In addition to the presynaptic A1R-mediated synaptic depression, previous studies also showed that A1R activation induced clathrin-mediated endocytosis of GluA2 subunits of AMPARs during cerebral hypoxia through the activation of *p38*-mitogen-activated protein kinases (*p38*-MAPKs) and c-Jun-N-terminal kinases (JNKs) [19,20,21]. Likewise, GluA1 subunits, phosphorylated at Ser831 or Ser845 residues, were found to undergo A1R-dependent clathrin-mediated internalization by three protein phosphatases: PP1, PP2A, and PP2B [21,22,23]. Collectively, the A1R-mediated downregulation of GluA1 and GluA2 subunits during ischemic conditions contributes to the synaptic depression and decreased neuronal excitability in the hippocampus during hypoxia, leading to the well-known neuroprotective effect of A1Rs [21,22,23]. Interestingly, previous reports also showed that the synaptic depression observed during hypoxia was followed by facilitated synaptic transmission and increased neuronal death following reperfusion of hippocampal slices in a mechanism dependent on both A1R and A2AR [24] and, in particular, involving the crucial role of A1Rs in downregulating the surface-expressed GluA1 and GluA2 AMPARs during hypoxia, followed by the upregulation of surface-expressed GluA1 while GluA2 subunits remained depressed during reperfusion [21,23,24].

Unlike the inhibitory A1Rs, the lower-affinity A2AR facilitates glutamatergic neurotransmission by increasing presynaptic glutamate release as reflected by paired-pulse ratio (PPR) depression in extracellular field excitatory postsynaptic potential (fEPSP) recordings [19,21]; thus, A2AR can contribute to glutamate excitotoxicity and subsequent neuronal death in cerebral hypoxia. Moreover, the balance between the inhibitory actions of A1R and the excitatory effect of A2AR plays an important role in the modulation of long-term potentiation (LTP) and AMPAR trafficking. In fact, the use of the A2AR antagonist attenuated LTP in aged rats since aged rats have a lower density of A1R and higher density of the excitatory A2AR, contributing to more glutamatergic neurotransmission and subsequent neurodegeneration [25,26]. Thus, changes in adenosine tone and surface expression of A1Rs and A2ARs play a crucial role in the modulation of LTP, synaptic plasticity, AMPAR trafficking, and glutamate-induced excitotoxicity [21,22,23,24]. Unfortunately, the usage of A1R agonists as possible neuroprotective agents in ischemia has failed in its translation into clinical practice due to severe peripheral side effects, which include sedation, bradycardia, and hypotension [27]. Furthermore, ischemia itself can induce changes in the expression of adenosine receptors by triggering the downregulation of A1Rs and the upregulation of A2ARs in insulted areas [21,28]. In addition, it was found that the higher-affinity inhibitory A1Rs undergo desensitization following 24–48 h from the onset of ischemia. The observed A1R downregulation results from the prolonged stimulation of A1Rs by the elevated extracellular adenosine in conditions such as hypoxia; thus, the neuroprotective effect of A1R stimulation is believed to be short-lived [21,22,23,24,29]. Interestingly, the A1R desensitization may cause indirect neurotoxicity due to shifting the action of the elevated extracellular adenosine to the lower-affinity excitatory A2AR. Accordingly, it was suggested that a possible A1R-A2AR cross-talk is a major contributor to glutamate excitotoxicity in cerebral hypoxia, since subjecting hippocampal slices to ischemic insult caused the downregulation of A1Rs but the upregulation of A2ARs [21,22,23,24,29].

Accordingly, A2AR antagonism has been proposed to be an alternative neuroprotective approach in in vivo models of cerebral ischemia since A2AR knockout mice showed less neuronal damage and infarct size following transient focal ischemia [30,31]. Previously, an A2AR blockade was shown to attenuate the anoxic depolarization and subsequent neuronal death in severe ischemia in the hippocampal CA1 region [32]. In addition, a previous report showed that the preincubation of hippocampal slices with an A2AR antagonist prevented the development of adenosine-induced post-hypoxia synaptic potentiation or APSP (previously known as anoxic long-term potentiation) and inhibited neuronal death caused by the excessive AMPAR-mediated synaptic transmission following hypoxia [24]. However, recent reports showed that the chronic stimulation of A1R also caused hippocampal and substantia nigral neurodegeneration associated with the overexpression and misfolding of α-synuclein [33,34,35], but whether this neurodegeneration correlated with increased A2AR levels remains unclear. Moreover, we previously reported in a rodent focal cortical stroke model (i.e., the unilateral pial vessel disruption (PVD) model) that both ipsilateral and contralateral hippocampal brain slices showed neurodegeneration and a significant downregulation of A1R but an upregulation of A2AR surface expression [21]. Moreover, this PVD model was associated with an increased density of microglia and astrocytes at the site of injury [36], but whether an increased density of glial cells also occurred away from the cortical injury (e.g., hippocampus) remains unresolved. As neuroinflammation is commonly associated with the activation of microglia and astrocytes [37,38,39] in neurodegenerative disorders such as stroke, PD, Alzheimer’s disease, and multiple sclerosis, we hypothesize that an increased surface expression of A2ARs in hippocampal neuronal and glial cells plays key regulatory roles in neuroinflammation, neuronal death, and synaptic potentiation. To understand how adenosine receptor stimulation after a PVD-induced stroke affects AMPAR-mediated excitatory synaptic transmission, neurodegeneration, neuroinflammation, and behavioral abnormalities, we examined the potential neuroprotective effects of the A2AR antagonist istradefylline in cerebral ischemia using both ex vivo and in vivo models of ischemic stroke. Istradefylline (KW-6002, (E)-8-(3,4-Dimethoxystyryl)-1,3-diethyl-7-methyl-3,7-dihydro-1H-purine-2,6-dione, Nourianz^®^) is the first novel A2AR antagonist approved by the US FDA as an adjunctive therapy with levodopa and carbidopa for the treatment of PD patients experiencing “OFF” symptoms and did not exacerbate dyskinesia caused by levodopa [40,41,42]. Here, we aimed to investigate whether this clinically approved and selective A2AR antagonist shows neuroprotective effects in our in vivo small-vessel-stroke model.

## 2. Results

### 2.1. Istradefylline Shows Neuroprotective Potential in Ex Vivo Ischemic Stroke Model

**Figure 1:**
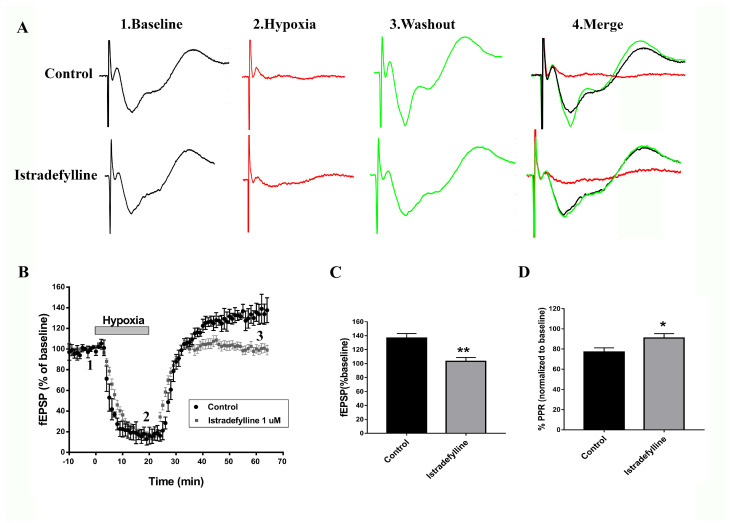
Istradefylline decreased presynaptic glutamate release and prevented the adenosine-induced post-hypoxia synaptic potentiation (APSP) following hypoxia/reperfusion. Hippocampal slices were preincubated with 1 µM istradefylline for 1 h before hypoxia. (**A**) Sample traces of the field excitatory postsynaptic potential (fEPSP) experiment showing an average of the last 5 min of the 10 min baseline in black color (1), 20 min hypoxia in red color (2), 45 min normoxic washout in green color (3), and an overlay of all traces (1 + 2 + 3). Scale bars show 10 ms (x) and 0.5 mV (y). (**B**) Time course graph showing normalized mean fEPSP of both control (no istradefylline in black color) and slices preincubated with istradefylline (gray color). (**C**) Summary bar graph showing the average fEPSP value as a percentage of the baseline (100%) in the last 5 min of normoxic washout. Istradefylline prevented the development of APSP, whereas control slices showed facilitated fEPSPs during normoxia. (**D**) Bar chart showing the % PPR calculated in the last 5 min of normoxic washout and normalized to baseline. All graphed values showed mean ± SEM. N = 6 independent fEPSP recordings per treatment group; N = 6 independent experiments for PI fluorescence images. Significance: * = *p* < 0.05 and ** = *p* < 0.01.

To determine whether istradefylline, an FDA-approved selective A2AR antagonist for PD, would prevent the previously reported adenosine-induced post-hypoxia synaptic potentiation (APSP) in hypoxia/reperfusion [24], hippocampal slices were subjected to 20 min of hypoxia followed by 45 min of normoxic perfusion as described before [23,24]. Figure 1A–C show that the hypoxia/reperfusion of hippocampal slices caused a significant elevation in synaptic transmission (i.e., APSP increased by 40%), which we previously showed to be dependent on AMPARs and A2ARs, since APSPs were abolished by the AMPAR antagonist perampanel and A2AR antagonist SCH442416 [24]. As shown in Figure 1A–C, the APSP was similarly abolished by the preincubation of slices with istradefylline; however, similar to SCH442416 [24], istradefylline did not affect the A1R-mediated synaptic depression occurring during hypoxia (Figure 1A,B). We then tested whether the enhanced synaptic transmission during APSP involves a greater probability of presynaptic glutamate release due to the increased activity of presynaptic A2ARs. Thus, we measured the paired-pulse ratio (PPR) as a function of presynaptic neurotransmitter release [19,21,24]. Interestingly, the APSP generated after hypoxia/reperfusion caused a significant 20% PPR depression compared to baseline before hypoxia (Figure 1D), suggesting enhanced presynaptic glutamate release, which underlies, in part, the observed APSP following hypoxia/reperfusion (Figure 1A–C; also see [24]). Moreover, the selective A2AR antagonist istradefylline markedly attenuated the PPR depression observed during normoxic reperfusion, indicating that this effect resulted from the inhibition of presynaptic neurotransmitter release after blocking the excitatory presynaptic A2AR (Figure 1D). In contrast, the PPR facilitation observed during hypoxia (i.e., ≈20% paired-pulse facilitation) was not altered by istradefylline.

### 2.2. Istradefylline Attenuated Hypoxia-Induced Hippocampal Cell Death

**Figure 2:**
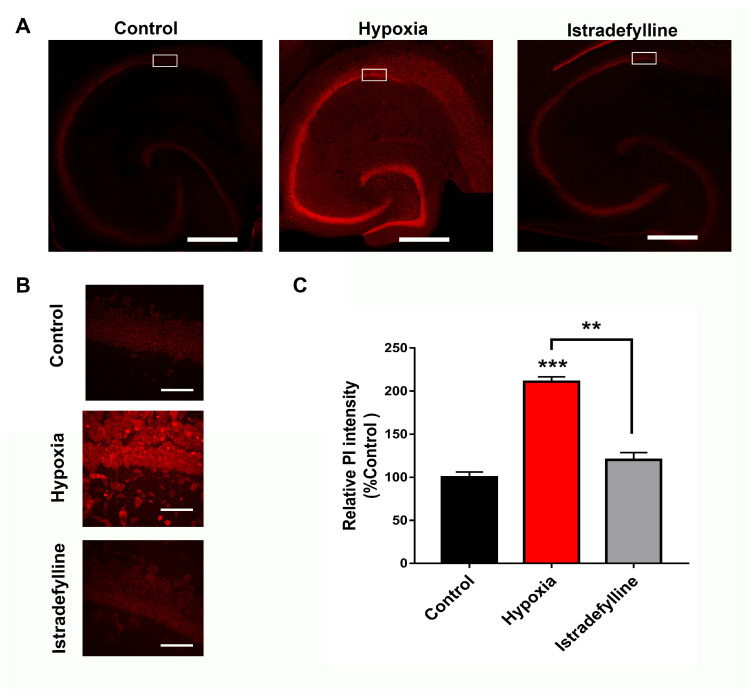
Administration of istradefylline significantly attenuated hippocampal cell death caused by hypoxia. Hippocampal slices were pretreated with 1 µM istradefylline for 1 h prior to hypoxia and stained with propidium iodide (PI), a fluorescent label for cell death. (**A**) Full hippocampal slices fluorescently stained with PI, taken at 10× magnification, showing PI fluorescence. Increased PI fluorescence indicates increased cell death. (**B**) Representative PI fluorescence images of the CA1 hippocampal cell layer (white boxed region in (**A**)) taken at 63× magnification. Scale bars: 1 mm (whole hippocampus, in (**A**)) and 10 µm (CA1, in (**B**)). (**C**) Bar graph showing analyzed densitometry values of the zoomed CA1 images (shown in (**B**)) to compare relative PI fluorescence intensity between treatment groups (control with no hypoxia in black color, hypoxia treated with vehicle control in red color, and hypoxia treated with istradefylline in gray color). All values were normalized to control (100%). All values showed mean ± SEM. Significance: ** = *p* < 0.01 and *** = *p* < 0.001. N = 6 independent experiments.

Since APSP caused neurotoxicity [24] and istradefylline prevented the APSP following hypoxia/reperfusion, we therefore hypothesized that the FDA-approved A2AR antagonist may have neuroprotective potential in ex vivo and in vivo animal models of cerebral ischemia. To test our hypothesis, we first used naïve hippocampal slices that were exposed to a hypoxia/reperfusion injury model and were later stained with propidium iodide (PI) as a fluorescent marker for cell death [21,23,24]. Figure 2 confirms the previously reported significant hippocampal cell death following hypoxia/reperfusion; however, the anti-Parkinsonian A2AR antagonist markedly decreased hypoxia-induced hippocampal cell death by ≈85%. These results suggest that istradefylline might have neuroprotective effects in cerebral ischemia.

### 2.3. Istradefylline Prevented PVD-Induced Memory Deficits

**Figure 3:**
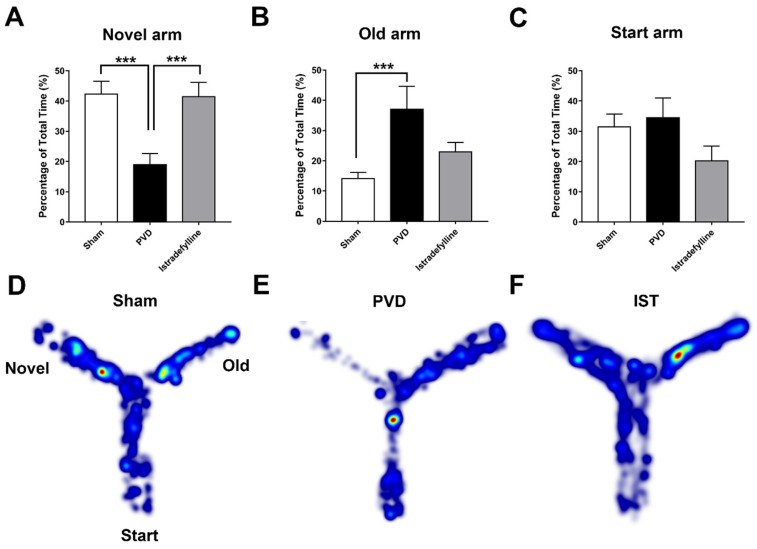
Administration of istradefylline prevented cognitive impairments caused by focal cortical ischemia. The PVD group exhibited the least time spent exploring the novel arm and the highest percentage of time spent in the old arm compared to the other treatment groups. Arm durations were calculated as a percentage of the 5 min second retrieval trial. (**A**–**C**) Bar graphs represent the percentage of time spent in novel, old, and start arms, respectively. (**D**–**F**) Representative heat maps of the 5 min second trial acquired from Ethovision for sham, PVD + DMSO/saline, and PVD + istradefylline 3 mg/kg, respectively. N values = 12 for each treatment group. Values are shown as mean ± SEM. Significance values: *** = *p* < 0.001.

We used the pial vessel disruption (PVD) focal cortical non-reperfusion ischemia model as previously described [21,36] to investigate the neuroprotective effects of istradefylline. First, we used the Y-maze assay to evaluate cognitive dysfunction occurring following ischemic stroke. Figure 3 shows that the daily administration of istradefylline for three days following PVD surgery significantly improved hippocampal-dependent spatial memory in rats subjected to PVD. Focal cortical ischemia mediated by PVD resulted in marked memory deficits (*p* < 0.001) as demonstrated by significantly less time spent in the novel arm (PVD–vehicle control-treated group; time spent in the novel arm of the Y-maze = 18.64 ± 3.89%) compared to the sham group (41.25 ± 3.46%, as shown in Figure 3A). On the other hand, PVD vehicle control-treated rats spent the most time (*p* < 0.05) exploring the old arm of the maze compared to sham and PVD–istradefylline-treated groups. Interestingly, the inhibition of A2ARs with istradefylline noticeably prevented PVD-induced memory deficits as reflected by the significantly increased time spent in the novel arm of the Y-maze (40.82 ± 4.58%, *p* < 0.001) compared to the PVD–vehicle control-treated group (18.64 ± 3.89%). In other words, the administration of istradefylline immediately after cerebral ischemic injury exhibited comparable results to the sham group and improved cognitive function.

### 2.4. Istradefylline Attenuated PVD-Induced Motor Deficits and Anxiety-like Behavior

**Figure 4:**
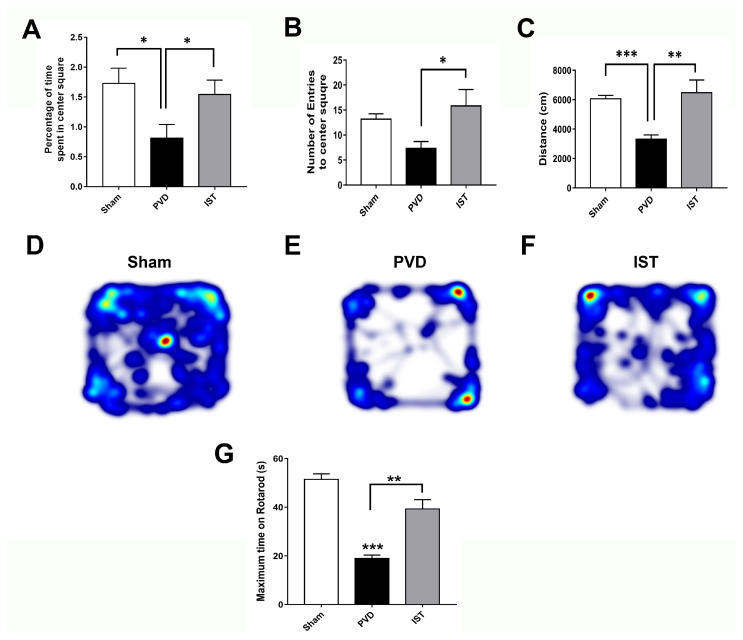
Istradefylline showed anxiolytic effects and inhibited motor deficits following ischemic stroke. Istradefylline significantly increased the percentage of time spent in the center square field and the number of entries into the center of the open field. In addition, A2AR antagonism restored motor activity and inhibited the reduction in distance moved by the rats in the maze during the task. (**A**) Bar chart showing the percentage of time spent in the center square of the field during the 15 min trial. (**B**) Bar chart showing the average number of entries into the center square of the maze during the task. (**C**) Bar chart showing the average distance (in cm) traveled by the rats in the field. (**D**–**F**) Representative heat maps from each treatment group were acquired by EthoVision. (**G**) Rats were pre-trained on the rotarod for 2 days before induction (two trials each day) and then tested 72 h following PVD surgery. The bar chart shows the average maximum time spent on the rotarod before falling. Istradefylline-treated rats preserved the overall motor function; however, the PVD–vehicle control group had the least latency before falling from the rotarod. N = 12 for each treatment group. Values are shown as mean ± SEM. Significance values: * = *p* < 0.05, ** = *p* < 0.01, and *** = *p* < 0.001.

Since motor dysfunction and post-stroke anxiety are common in patients following recovery from ischemic stroke [43,44,45], we used the open field test (OFT) to detect possible motor deficits and anxiety-like behavior following the induction of focal cortical ischemia. As shown in Figure 4A–F, the PVD group treated with the vehicle control showed significantly less time exploring the center square of the box arena compared to the sham group and spent most of the time exploring the edges of the square field (*p* < 0.05), indicating fear and anxiogenic behaviors occurring post-PVD. However, istradefylline treatment markedly increased not only the percent of time spent in the center square of the arena but also the number of entries into the center square compared to vehicle control-treated rats, indicating an anti-anxiety behavioral effect of istradefylline (Figure 4). Moreover, focal cortical ischemia caused by a PVD lesion caused a marked ≈ 50% reduction in the distance traveled by the rats within the maze during the 15 min, suggesting that the PVD lesion resulted in severe motor deficits (*p* < 0.001) (Figure 4C,E). In contrast, the administration of istradefylline not only prevented the anxiety-like behavior caused by PVD but also preserved motor function (*p* < 0.01). As shown by the heat maps and the calculated distance traveled by the rats during the trial, the administration of istradefylline following PVD showed comparable results to the sham group (Figure 4C–F). Moreover, motor function assessed with the rotarod test showed that PVD treatments significantly reduced the ability of rats to remain on the rod compared to sham animals, and istradefylline treatment following PVD procedure significantly attenuated this PVD-induced motor deficit (Figure 4G). Therefore, the observed motor deficits caused by PVD, which were attenuated by istradefylline, suggest a crucial role of A2AR in motor dysfunction following ischemic stroke.

### 2.5. Istradefylline Attenuated Post-Stroke Depression

**Figure 5:**
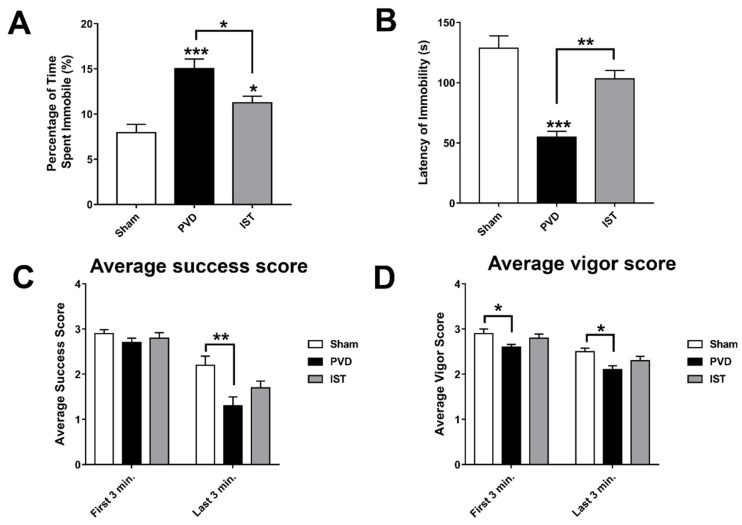
Istradefylline prevented depressive-like behavior. Rats were placed in a water tank for 10 min, and time spent immobile was calculated as a percentage of the total trial time. Latency to immobility was plotted as times in seconds. (**A**) The PVD–istradefylline-treated group attenuated the depressive-like behavior and showed a 50% reduction in time spent immobile compared to the vehicle control-treated group. (**B**) Istradefylline treatment showed 95% improvement in the latency time of immobility. (**C**,**D**) Average success and vigor scores were estimated as mentioned in the Methods Section. N = 12 for each treatment group. Values are shown as mean ± SEM. Significance values: * = *p* < 0.05, ** = *p* < 0.01, and *** = *p* < 0.001.

In addition to anxiety caused by PVD, we tried to identify other behavioral abnormalities following cerebral ischemia such as depression. In fact, many patients experience clinical depression following a stroke [46]. We used the forced swim test (FST) to monitor depressive-like behavior and further validate the observed motor deficits in the rotarod task and OFT. Interestingly, the PVD–vehicle control group showed 40% and 17% more time spent immobile compared to sham and PVD–istradefylline-treated groups, respectively (Figure 5A,B). In addition, PVD caused a significant reduction in the latency time of immobility, suggesting despair and depressive behavior. However, treatment with the selective A2AR antagonist istradefylline resulted in marked improvement in the latency time of immobility compared to the PVD–vehicle control group (*p* < 0.01). Moreover, in the last 3 min of the FST, the focal cortical ischemia caused a significant decrease in time spent swimming with their heads above water and swimming using all four limbs as indicated by low success and vigor scores, respectively (Figure 5C,D). Nevertheless, istradefylline partially improved both success and vigor scores. These results suggest that A2AR antagonism can be a potential target for the prevention of post-stroke depression and motor deficits.

### 2.6. Istradefylline Inhibited PVD-Induced Hippocampal Cell Death

**Figure 6:**
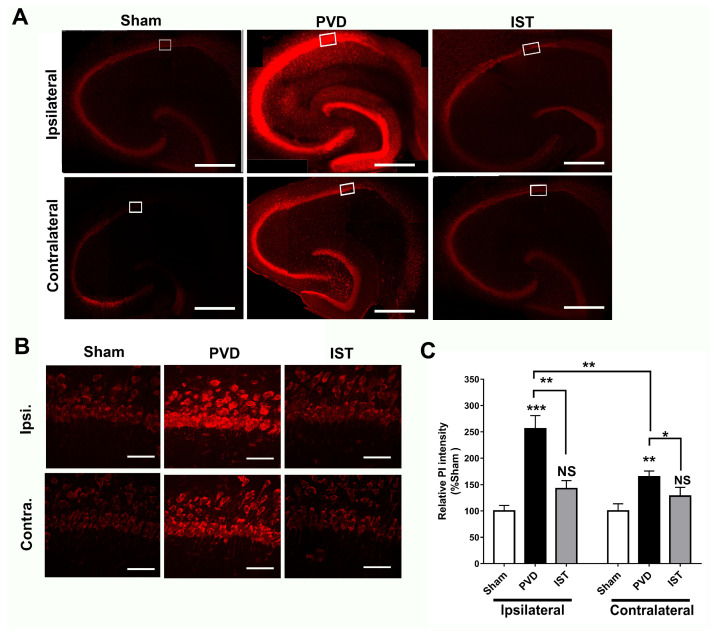
Istradefylline reduced hippocampal cell death caused by focal cortical ischemia. The administration of istradefylline (IST) showed neuroprotective properties and decreased hippocampal cell death in both ipsilateral and contralateral sides. Hippocampal slices were stained with propidium iodide (PI), a fluorescent marker for cell death. (**A**) Full montage of hippocampus showing PI fluorescence obtained with 10×. Increased PI fluorescence indicates increased cell death. (**B**) Magnification of 63× of the representative squares in A (white boxed region) showing the CA1 area of the hippocampus stained with PI, respectively. Scale bars: 1 mm (whole hippocampus, in (**A**)) and 10 µm (CA1, in (**B**)). (**C**) Summary bar graph showing relative PI intensity of the CA1 images shown in squares in (**A**) compared to the corresponding sham group. Levels of hippocampal neuronal damage were lower in the contralateral compared to the ipsilateral side of the PVD lesion. N = 5 for each treatment group. Values are shown as mean ± SEM. Significance values: * = *p* < 0.05, ** = *p* < 0.01, and *** = *p* < 0.001. NS = non-significant.

Since istradefylline treatment preserved memory and motor activity and attenuated anxiogenic and depressive behaviors in rats following the induction of ischemic stroke, we therefore tried to investigate the underlying cellular mechanisms for the improved functional outcomes following istradefylline treatments in our in vivo stroke model. Figure 6 shows that focal cortical ischemia induced by PVD surgery resulted in a marked increase in hippocampal cell death by 150% and 60% in the ipsilateral and contralateral sides of the hippocampus, respectively, compared to their corresponding sham hippocampal slices. It is important to note that the PVD lesion not only caused marked hippocampal cell death in the ipsilateral side but also triggered significant ischemic damage in the contralateral hippocampus. Indeed, cerebral ischemia caused a notable 50% more hippocampal cell death in the ipsilateral side compared to the contralateral part (*p* < 0.01). Interestingly, A2AR antagonism with istradefylline significantly attenuated hippocampal cell death in both ipsilateral and contralateral sides of the hippocampus, suggesting potential neuroprotective effects of this anti-Parkinsonian medication in cerebral ischemia.

### 2.7. Istradefylline Decreased Hippocampal Neurodegeneration Caused by Focal Cortical Ischemia

**Figure 7:**
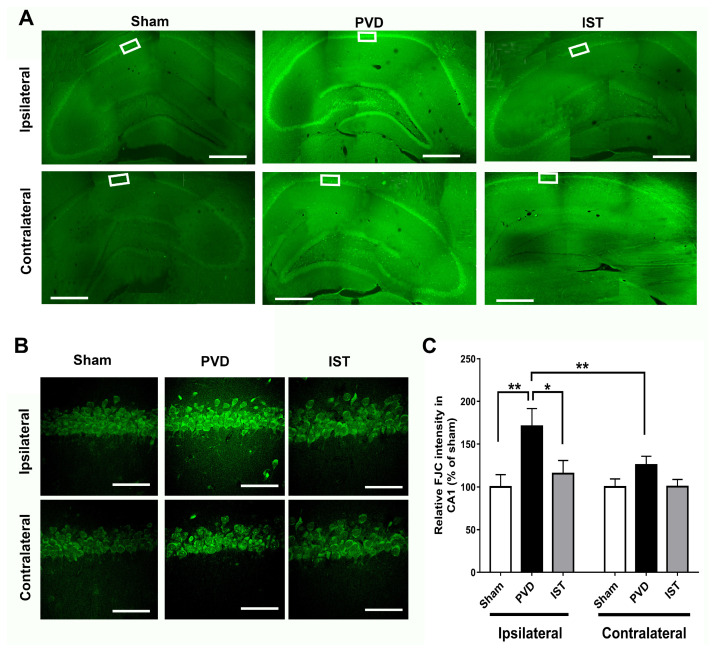
Istradefylline attenuated PVD-induced hippocampal neurodegeneration in both ipsilateral and contralateral sides. The administration of istradefylline attenuated neurodegeneration in the hippocampus following PVD. Coronal hippocampal slices were stained with FluoroJade C (FJC), a specific fluorescent marker for degenerating neurons. (**A**) Full montage of hippocampus fluorescently stained with FJC obtained with 10×. (**B**) Magnification of 63× of the representative squares in A (white boxed region) showing the CA1 area of the ipsilateral and contralateral hippocampus, respectively. Scale bars: 1 mm (whole hippocampus, in (**A**)) and 40 µm (CA1, in (**B**). (**C**) Summary bar graphs showing relative FJC intensity of the CA1 images shown in squares in (**A**). N = 5 for each treatment group. Values are shown as mean ± SEM. Significance values: * = *p* < 0.05 and ** = *p* < 0.01.

To further confirm the observed results from PI staining, we used FluoroJade-C (FJC) as a specific fluorescent marker for degenerating neurons. As expected, focal cortical ischemia caused significant neurodegeneration in the hippocampus on the ipsilateral side and to a lesser extent in the contralateral part. Figure 7 shows marked elevation in the fluorescence intensity of ipsilateral hippocampal neurons positively stained with FJC, indicating significant neurodegeneration following cerebral ischemia compared to the sham group (*p* < 0.01). However, the A2AR antagonist istradefylline significantly attenuated the PVD-induced hippocampal neurodegeneration in the ipsilateral side of the PVD lesion (*p* < 0.05) and showed comparable results to the sham group.

### 2.8. Istradefylline Inhibited Microglia- and Astrocyte-Induced Neuroinflammation

**Figure 8:**
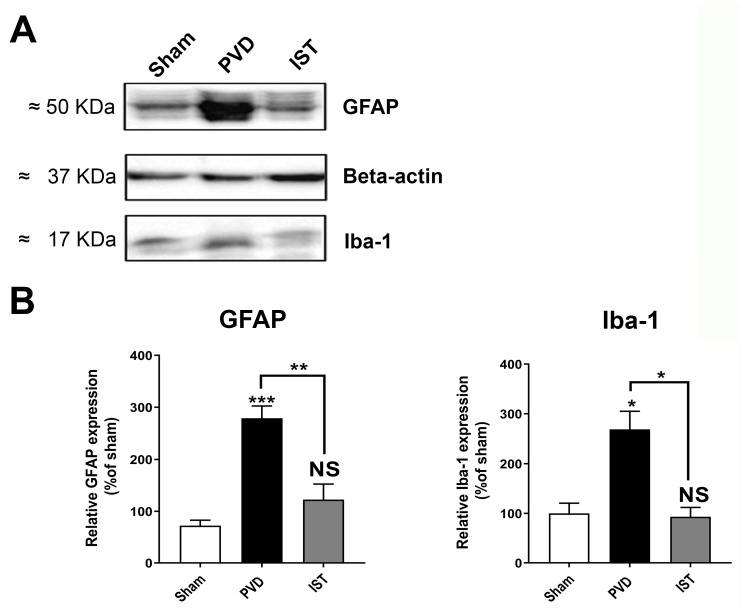
Istradefylline inhibited the activation of microglia and astrocytes. (**A**) Representative Western blot of hippocampal lysates showing expression levels of GFAP (marker for astrocytes), Iba-1 (marker for microglia), and beta-actin (loading control); N = 8 independent observations. (**B**) Summary bar charts showing a significant upregulation of GFAP and Iba-1 in PVD-treated rats and reversal by istradefylline. Values are expressed as mean ± SEM. Statistical significance was assessed using a one-way ANOVA test and a Tukey–Kramer multiple comparison test with a 95% confidence interval. Significance values: * = *p* < 0.05, ** = *p* < 0.01, and *** = *p* < 0.001. NS = non-significant.

**Figure 9:**
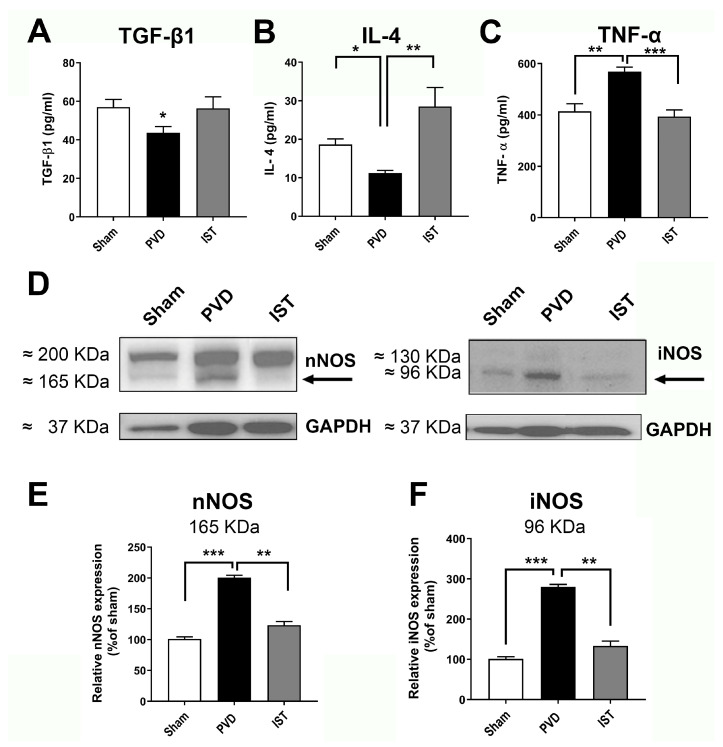
Istradefylline restored the balance between inflammatory and anti-inflammatory mediators following PVD. Treatment with istradefylline restored the levels of anti-inflammatory mediators TGF-β1 and IL-4 after PVD surgery (**A**,**B**). The PVD-induced elevation of inflammatory mediators TNF-α and iNOS (as well as nNOS) was attenuated with istradefylline treatment (**C**–**F**). (**A**–**C**) The total concentrations of TGF-β1, IL-4, and TNF-α were obtained using ELISA from ipsilateral hippocampal lysates. (**D**) Representative Western blot images of total ipsilateral hippocampal tissue lysates (left panel, nNOS and GAPDH; right panel, iNOS and GAPDH). (**E**,**F**) Summary bar charts showing relative expressions of nNOS (**E**) or iNOS (**F**) (% of sham). The administration of istradefylline prevented the PVD-induced increase in both nNOS (165 kDa monomeric nNOS; ~200 kDa believed to be nNOS bound to calmodulin) and iNOS (96 kDa iNOS cleavage product from the 130 kDa iNOS; see faint band above the 96 kDa signal). All tissue lysates were prepared 72 h following surgery. Values are shown as mean ± SEM. N = 5 in each group (independent samples). Significance values: * = *p* < 0.05, ** = *p* < 0.01, and *** = *p* < 0.01.

Consistent with a reduction in neuroinflammation contributing to the attenuation of hippocampal neurodegeneration after a stroke, we determined whether astrocyte and microglia markers are altered by istradefylline. As shown in Figure 8, both the astrocyte marker GFAP and the microglia marker Iba1 were significantly reduced when animals were treated with 3 mg/kg istradefylline following cerebral ischemia. In addition, istradefylline treatment restored the levels of pro-inflammatory and anti-inflammatory mediators. As shown in Figure 9, istradefylline significantly attenuated the elevation of microglia and astrocyte pro-inflammatory markers (inducible nitric oxide synthase (iNOS) and tumor necrosis factor-alpha (TNF-α)) as well as the neuronal NOS (nNOS) following cerebral ischemia induced by PVD surgery (Figure 9C–F). Moreover, istradefylline preserved the levels of anti-inflammatory markers of microglia and astrocytes, including TGF-β and IL-4. These results suggest a neuroprotective potential of istradefylline to suppress neuroinflammation following cerebral hypoxia.

## 3. Discussion

Ischemic stroke, which occurs due to the disruption of blood flow to the brain, has been increasingly recognized as a significant risk factor in the pathogenesis of various neurodegenerative disorders, including Alzheimer’s disease (AD) and Parkinson’s disease (PD) [9,10,47]. The damage caused by ischemic stroke not only leads to immediate motor and cognitive impairments but also sets in motion a cascade of cellular and molecular events that can accelerate neurodegeneration. In the present study, we showed that ischemic stroke resulted in Parkinsonism-like pathology involving motor and non-motor behavioral deficits, which are abrogated by the anti-Parkinsonian drug istradefylline. This selective A2AR antagonist reduced neuronal damage and neurodegeneration in the hippocampus, in part by reducing neuroinflammation from astrogliosis and microgliosis. The increased activity of A2ARs has been linked to the modulation of processes such as mood [48], learning and memory [26], and neurodegeneration in stroke or hypoxia, as well as other neurodegenerative diseases [22,29].

The present study further investigated the interplay between A1Rs and A2ARs in synaptic transmission, neurodegeneration, and behavioral abnormalities after cerebral ischemia. In particular, we focused on the role of A2ARs, which have been shown to be upregulated in ex vivo and in vivo models of cerebral ischemia [21,23,24,25]. We found that preincubation of naïve slices with the A2AR antagonist istradefylline before exposure to hypoxia/reperfusion injury protocol abolished the adenosine-induced post-hypoxia synaptic potentiation or APSP, which was suggested to be mediated by increased calcium-permeable AMPARs [24]. Using paired-pulse ratio (PPR) recordings, we determined that a presynaptic component may underlie, in part, the APSP that develops after normoxic reperfusion. The observed PPR facilitation during hypoxia, which also occurred during selective A1R stimulation [19], indicates a presynaptic A1R-mediated inhibition of glutamate release, which explains the observed synaptic depression during hypoxia. In contrast, blocking A2ARs with istradefylline did not inhibit either the synaptic depression or the increased PPR occurring during hypoxia. Moreover, we recently showed that A1R antagonist DPCPX completely abolished the hypoxia-mediated reduction in fEPSP during hypoxia [24]. Accordingly, this study and our recent findings, collectively, strengthen the hypothesis that the decreased presynaptic glutamate release and subsequent synaptic depression observed during the early phase of hypoxia insult are mainly caused by presynaptic inhibition from A1R stimulation. Similar to hypoxia, the stimulation of A1R by the selective agonist CPA resulted in facilitation in PPR with subsequent synaptic depression through JNKs and p38-MAPKs [19,20,21]. However, the enhanced fEPSP following hypoxia and normoxic reperfusion, called APSP, results from increased presynaptic glutamate release as well as increased calcium-permeable AMPARs and A2ARs [21,23,24]. Since prolonged stimulation of the higher-affinity A1R by the elevated extracellular adenosine during hypoxia results in A1R desensitization [21,23], the enhanced action of the lower-affinity A2ARs following the reperfusion of a normoxic solution is expected to increase presynaptic glutamate release and, hence, enhance synaptic transmission, which were both found to be significantly attenuated by istradefylline. Along with the observed prevention of neuronal injury after hypoxia or PVD-induced focal cortical ischemia by istradefylline, the present results confirm that istradefylline attenuates the pro-excitotoxic effects of AMPAR-mediated APSPs due to hyperactive A2ARs. This istradefylline-induced prevention of enhanced synaptic transmission following normoxic reperfusion strongly indicates that A2ARs play a crucial role in the elevation of extracellular glutamate concentrations via a presynaptic mechanism and in glutamate excitotoxicity via a postsynaptic A2AR-mediated modulation of AMPARs [24] in cerebral ischemia. The increased presynaptic glutamate release caused by shifting the action of elevated extracellular adenosine mainly on the excitatory Gα_s_-coupled A2ARs following the downregulation of the inhibitory neuroprotective A1Rs is a major factor that is widely recognized to promote neurotoxicity [15,21,22,23,29,49].

Moreover, we recently described a cross-talk between A1R and A2AR involving the casein kinase 2 (CK2) that contributes to the observed neuronal damage during a 20 min hypoxia/reperfusion injury model [21,23,24]. We suggested that a prerequisite for the A2AR surface upregulation and A2AR-induced APSPs to occur is a prior A1R stimulation and its subsequent desensitization, as the effects of hypoxia on A2AR-induced APSPs and neuronal damage are absent with an A1R blockade with DPCPX [24]. We also found that CK2 inhibitors increased A2AR surface expression before hypoxia, which is consistent with previous reports showing the negative regulation of A2ARs and other Gα_s_-coupled GPCRs by CK2 [50]. Interesting, this increased A2AR surface expression was also observed after a 20 min hypoxia [24] and three days after PVD-induced focal cortical ischemia [21], raising the possibility that this increased A2AR surface expression may be caused by decreased CK2 function or expression as previously suggested to occur in various neurological diseases [51,52]. However, it is notable that pretreatment with CK2 inhibitors before hypoxia reduced the hypoxia-induced APSP and A2AR surface expression and significantly reduced hippocampal neuronal damage [24]. Consistent with these observations, the present study showed that istradefylline treatment prior to hypoxia or PVD-induced focal cortical ischemia caused significant neuroprotection, further highlighting the role of upregulated A2AR surface expression in ischemic injury. Interestingly, other studies have described A2AR’s control of synaptic transmission and modulation of glutamate excitotoxicity, and A2ARs have been suggested to be a potential target for many neurodegenerative disorders [22], including temporal lobe epilepsy [53], Parkinson’s disease [6], Alzheimer’s disease [54,55], and stress-induced LTP deficits [56]. However, future in vivo studies are needed to further clarify the role of CK2 in the cross-talk of A1R/A2AR activities and how they affect the trafficking of AMPAR subunits during PVD-induced neuronal damage.

In addition to the neuroprotective effect of istradefylline in ex vivo hippocampal slices subjected to hypoxia, this study also provides additional evidence of the neuroprotective effect of istradefylline in an animal model of ischemic stroke. The above results showed that the istradefylline pretreatment of PVD rats resulted in significantly improved cognitive function as observed in the Y-maze task. Similarly, istradefylline prevented cognitive deficits in the novel object recognition task in an animal model of PD induced by 6-hydroxydopamine lesioning [57]. Moreover, istradefylline (KW-6002) attenuated learning and memory deficits and improved performance in the Morris water maze task in rats subjected to stress following maternal separation [56] or chronic stress [58]. In addition to the preserved cognitive function observed in animal models of PD [57] and ischemic stroke presented in this study, the FDA-approved non-dopaminergic anti-Parkinsonian istradefylline attenuated memory deficits in an animal model of AD [54]. Likewise, SCH58261, another selective A2AR antagonist, improved synaptic plasticity and performance in the Y-maze task in an animal model of AD [59,60]. Interestingly, a recent report showed that the impaired hippocampal-dependent spatial memory in AD was linked to the upregulation of A2ARs and their aberrant interaction with mGluR5/NMDAR [55]. Thus, an early blockade of the upregulated A2ARs following cerebral ischemia by istradefylline can be a potential therapeutic approach to prevent cognitive and memory deficits post-stroke. It is noteworthy that istradefylline and other A2AR antagonists failed to improve memory in control naïve animals; however, their effect on memory improvement was dependent on the prevention of the deterioration of cognitive function in brain disorders such as AD [59,60] and ischemic stroke (present study). This could be explained by the fact that A2ARs are less expressed in the hippocampus under normal conditions; however, A2ARs are upregulated in brain disorders such as AD, cerebral ischemia, and PD [56,58,61,62].

The administration of istradefylline following PVD significantly increased the distance traveled by rats and the latency of falling in the OFT and rotarod test, respectively. These results indicate that A2AR upregulation in PVD [42,63,64] is associated with increased motor deficits (present study), and A2AR blockades with istradefylline preserved motor activity in our in vivo model of small-vessel stroke. Similarly, both selective and non-selective A2AR antagonists (istradefylline and caffeine, respectively) exerted neuroprotective effects and improved motor activity in the 1-methyl-4-phenyl-1,2,3,6-tetrahydropyridine (MPTP) model of PD [65,66]. In fact, the preserved motor activity in PVD rats treated with istradefylline may be attributed to the inhibition of striatal dopaminergic neuronal death due to A2AR antagonism [67]. Moreover, previous studies showed that istradefylline can prevent MPTP-induced striatal neurodegeneration of dopaminergic neurons. Indeed, A2AR genetic knockout mice showed less MPTP-induced neurotoxicity and improved locomotor activity [65,66]. In addition to the observed improved motor activity with istradefylline in animal models of PD, the selective A2AR antagonist significantly reduced ‘OFF’ symptoms in clinical trials, which was the basis for the FDA approval of istradefylline for the treatment of PD in conjunction with the conventional levodopa [42,63,64]. Furthermore, an epidemiological study showed that consumption of caffeine, the non-selective A2AR antagonist, decreased the risk of PD in both men and women [68]. Interestingly, the preserved motor activity in istradefylline-treated rats following the induced ischemic stroke may be attributed to a potential complex interaction with the dopaminergic pathway, since A2AR antagonism results in the enhancement of D2R signaling, resulting in an inhibition of the indirect GABAergic pathway of the substantia nigra and thalamus, which suppress motor activity [69]. Previous studies showed that the observed improved motor function in istradefylline-treated PD patients could be linked to additional pharmacological activity, in addition to A2AR selective antagonism. A previous in silico study showed that istradefylline could have partial agonist activity on D2/D3 receptors [70,71]. However, further molecular studies are yet to confirm this prediction.

In addition to the preserved motor activity and attenuation of cognitive impairment with istradefylline treatment, this study also shows novel evidence that istradefylline inhibited behavioral disability, including anxiety and depression, following cerebral ischemia. The observed increased time spent in the center square in OFT, as well as the increased latency to immobility in the FST of PVD rats treated with istradefylline, is consistent with the results of previous studies, which suggested that A2ARs play a vital role in causing post-stroke anxiety and depression [72,73]. In agreement with these results, a previous study showed that the administration of istradefylline resulted in a significant reduction in immobility time in FST in the PD model in both rats and mice, suggesting an antidepressant effect of the selective A2AR antagonist istradefylline [74]. Similarly, another study showed that the pharmacological blockade of A2ARs with SCH58261 attenuated anxiety-like behavior in the AD model as displayed by a significant increase in time spent in the center square in OFT and in the open arms of the elevated plus maze task [59]. In addition, Kaster et al. reported that istradefylline showed anti-anxiety and antidepressant effects in rats subjected to chronic stress [58]. Previously, it was thought that the improved performance in FST and OFT (observed as decreased immobility time and increased distance traveled, respectively) could be attributed to the positive effect of the A2AR antagonist on motor activity and not due to its antidepressant or anxiolytic actions, which may lead to false positive results. However, a previous study showed that istradefylline inhibited anxiety and stress caused by maternal separation through neuromodulation of the hypothalamic–pituitary–adrenal axis (HPA-axis), where A2AR antagonism resulted in a reduction in plasma corticosteroid levels and decreased surface expression of hippocampal glucocorticoid receptors, which were elevated following stress conditions [56]. Moreover, a previous report showed that the antidepressant effect of istradefylline observed in FST with rats and mice was similar to the conventional tricyclic antidepressants (TCAs). In addition, the antidepressant effect of istradefylline was abolished by the co-administration of corticosterone in a small dose that does not affect motor activity, suggesting that istradefylline has antidepressant properties and the improved performance in FST is not mainly due to preserved motor activity [74]. However, more studies are needed to further examine whether the PVD-induced upregulation of A2AR [21] and possible modulation of the HPA axis are both involved in causing anxiety and depression following cerebral ischemia. Therefore, the present study provides novel evidence that the FDA-approved A2AR antagonist istradefylline can be a promising therapy in mood disorders such as post-stroke anxiety and depression.

In this study, we showed the crucial role of A2AR antagonism in the attenuation of inflammatory cytokines and other factors from both microglia and astrocytes in cerebral ischemia. We previously reported that minocycline and the matrix metalloprotease-2 and -9 (MMP-2 and MMP-9) inhibitor batimastat decreased lacuna-like cavity formation in the PVD stroke model and decreased the density of microglia and astrocytes at the cortical injury site [36]. Moreover, using the same PVD stroke model, we previously reported that A1Rs were downregulated while A2ARs were upregulated in both the ipsilateral and contralateral hippocampus [21], suggesting that this PVD stroke model may also promote microglia and astrocyte activation at distant sites away from the original ischemic injury location. The observed inhibition of pro-inflammatory mediators isolated from the hippocampus, such as TNF-α, iNOS, and nNOS, accounts for the improved cognitive function and motor activity observed in istradefylline-treated rats. Similarly, recent studies showed the neuroprotective potential of A2AR antagonism through the inhibition of microglia activation and the suppression of microglial pro-inflammatory cytokines in multiple neurodegenerative disorders, including PD and AD [75,76,77,78]. In this study, we showed that istradefylline inhibited hippocampal cell death and neurodegeneration through the inhibition of microglia and astrocyte activation and the promotion or restoration of the expression of anti-inflammatory mediators, including TGF-β1 and IL-4. Therefore, the improved cognitive, mood, and motor behaviors observed in PVD animals administered with istradefylline indicate the neuroprotective effects of istradefylline and its potential in reducing the risk of developing post-stroke neurodegenerative disorders like dementia, PD, and AD. However, more studies are yet to investigate the impact of A2AR antagonism in ischemic stroke in order to slow the progression of AD or PD.

The majority of neuroprotective agents that have been shown to be neuroprotective in preclinical animal trials have failed to be translated into clinical practice in ischemic stroke patients because adverse effects or a lack of therapeutic efficacy was observed [79,80]. Therefore, most preclinical animal studies must now adhere to the STAIR (Stroke Therapy Academic Industry Roundtable) criteria, which are guidelines and recommendations for conducting preclinical trials for neuroprotective and restorative agents in animal stroke models [81,82]. Examples include carefully choosing the therapeutic window for drug initiation to achieve optimal drug efficacy, as well as conducting a long-term study to follow up the behavioral and physiological changes after stroke injury [83]. Another factor that needs to be addressed is whether the neuroprotective effect of istradefylline is dependent on the continuation of drug administration in the long term, or whether the three-day single-daily-dose administration of istradefylline in the acute phase will be sufficient to prevent the long-term neuronal damage and cognitive deficits caused by stroke. Furthermore, the potential mechanisms through which istradefylline acts to prevent stroke-induced neurodegeneration need to be further investigated through the longer-term studies and using other stroke models such as the middle cerebral artery occlusion (MCAO) model. Additionally, it is important to determine if there are sex differences in the adenosinergic receptor system that may influence the recovery mechanisms and possible differential response to istradefylline in males and females [84]. It has been known that older premenopausal females have a lower risk of stroke incidence compared to males of the same age because of the effect of estrogen on decreasing cardiovascular complications [85]. Moreover, previous reports have shown that estrogen exerts a neuroprotective effect in cerebral ischemia [86,87]. Therefore, future studies are needed to confirm using juvenile and middle-aged female and male rats to determine if the levels of neurodegeneration, behavioral abnormalities, and changes in neuroinflammatory markers are significantly attenuated in female rodents to rule out any potential sex differences in the response to therapeutic treatments.

In summary, the neuroprotective potential of the A2AR antagonist istradefylline suggests that this anti-Parkinsonian drug could be repurposed for stroke neuroprotective therapy. Our findings showed that istradefylline significantly attenuated neuronal death and neurodegeneration after PVD-induced focal cortical ischemia, and this was strongly correlated with significant improvements in motor and non-motor behavioral deficits. The administration of istradefylline in focal cortical cerebral ischemia may be effective in countering the effects of upregulated A2AR expression subsequent to stroke damage, which contributes to increased neuroinflammation, neurodegeneration, and behavioral abnormalities in our in vivo focal cortical stroke model. Finally, the current findings suggest that there may be shared molecular and biochemical pathways linking stroke and neurodegenerative diseases, which warrants further studies to test whether istradefylline and other clinically approved drugs indicated for other diseases could be repurposed for neuroprotective/adjunctive therapy in neurodegenerative disease.

## 4. Materials and Methods

### 4.1. Animal Subjects

Male *Sprague-Dawley* rats at 25–35 days old (Charles River Canada, Montreal, QC, Canada) were used in all studies. We used only male rats to test our hypothesis to avoid any biological variations that might be caused by changes in the levels of estrogen during the estrous cycle in female rats. Estrogen can be a confounder during electrophysiology recordings since previous studies reported that estrogen has a neuroprotective effect in cerebral ischemic injury [88] and heart ischemia/reperfusion [89].

**Figure 10:**
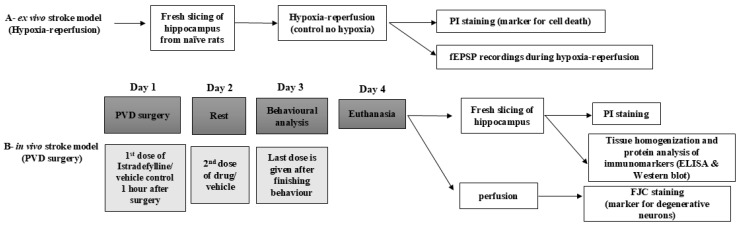
Schematic illustration of the ischemic stroke induction and treatment protocol to investigate the potential neuroprotective effects of istradefylline in a small-vessel-stroke model in male rats and in an ex vivo stroke model (hypoxia/reperfusion). Focal cortical ischemia was induced by pial blood vessel disruption (PVD) as described in the Methods Section. Rats subjected to PVD received either a vehicle control or istradefylline 1 h after the surgery. Behavioral tasks were performed on the third day to assess post-stroke memory deficits and other behavioral abnormalities. On the fourth day, the rats were sacrificed for subsequent post-mortem analysis of brain tissue samples.

After arrival, the animals were kept for at least 1 week for handling before being used in any procedure (see Figure 10 for timelines and list of all procedures and animal treatments). Rats were housed two per cage in standard polypropylene cages in a temperature-controlled (21 °C) colony room on a 12/12 h light/dark cycle. Experimental procedures were carried out during the light phase. Rats were randomly divided into three groups (n = 15 in each group) based on the surgery described below and received the following treatments. The first group was the control group (sham), while both the other two groups were subjected to PVD surgery; group 2 denotes the PVD/vehicle control group referred to in the graphs as (PVD), and treatment group 3 denotes the PVD/istradefylline group, with a selective A2AR antagonist, referred to as (IST) as the treatment group.

Another group of naïve rats was used for electrophysiology studies in our ex vivo ischemic/reperfusion stroke model.

### 4.2. Hippocampal Slice Preparation and Electrophysiological Recordings

On the fourth day after PVD surgery, male *Sprague-Dawley* rats from 3 groups (sham, PVD + DMSO, and PVD + istradefylline) were anesthetized with halothane and rapidly decapitated, with the brains immediately excised and submerged in an oxygenated, ice-cold high-sucrose dissection medium containing the following: 87 mM NaCl, 25 mM NaHCO_3_, 25 mM glucose, 75 mM sucrose, 2.5 mM KCl, 1.25 mM NaH_2_PO_4_, 7.0 mM MgCl_2_, and 500 μM CaCl_2_ [19]. Hippocampal slices from both the ipsilateral and contralateral sides of the lesion were taken at 400 μm thickness using a vibrating tissue slicer (VTS1200S, Leica Biosystems, Heidelberg, Germany), and slicing was performed in the same ice-cold oxygenated dissection medium as above. Slices were maintained for at least 1 h at room temperature before performing any experiments in oxygenated artificial cerebrospinal fluid (aCSF) containing the following: 126 mM NaCl, 2.5 mM KCl, 2.0 mM MgCl_2_, 1.25 mM NaH_2_PO_4_, 26 mM NaHCO_3_, 10 mM glucose, and 2.0 mM CaCl_2_ [19]. Oxygenation was accomplished by continually bubbling the solution with 95% O_2_/5% CO_2_. Electrophysiological recordings from hippocampal brain slices were previously described [19,20,21,22,23,24,25].

### 4.3. Drug Treatments

Rats subjected to PVD surgery are injected intraperitoneally with istradefylline 3 mg/kg or the vehicle control 1 h after the surgery and once daily for 3 consecutive days. Istradefylline was dissolved in 0.9% saline (DMSO, Sigma, St. Louis, MO, USA) and administered at a dose of 3 mg/kg after recovery from PVD surgery. A preliminary experiment was performed to choose the minimal effective dose to inhibit behavioral deficits following PVD surgery. However, we finally used 3 mg/kg istradefylline, as other previous studies have used a similar dose, which was found to be safe and effective in rats for the prevention of motor deficits and retinal toxicity [77,90]. Istradefylline was purchased from Bioorbyt Ltd (cat #orb1300666, Cambridge, UK). For electrophysiology and propidium iodide staining, istradefylline was first dissolved in DMSO and diluted in aCSF to the desired final concentration of 1 µM.

### 4.4. Biochemistry and Western Blotting

Hippocampal slices from the treatment groups (sham, pial vessel disruption (PVD) alone, and PVD + istradefylline) were prepared as previously described [42,63,64]. Slices were then transferred to homogenization tubes and homogenized in a lysis buffer (pH 8.0) containing 50 mM Tris, 150 mM NaCl, 1 mM EDTA, and 1 mM NaF, as well as the following protease inhibitors: 1 mM PMSF, 10 µg/mL aprotinin, 10 µg/mL pepstatin A, 10 µg/mL leupeptin, 2 mM Na_3_VO_4_, 20 mM sodium pyrophosphate, 3 mM benzamidine hydrochloride, and 4 mM glycerol 2-phosphate with 1% NP-40 detergent. After tissue homogenization, a Bradford Assay was performed with DC Protein assay dye (Bio-Rad, Canada, Mississauga, ON, Canada) to determine protein concentration in the lysates. Protein lysates (50 µg) from the different treatment groups were diluted with 20 µL of 3× Laemmli sample buffer, boiled for 5 min, and then separated in 12% SDS-PAGE gels for 20 min at 80 V followed by 1 h at 160 V. Proteins were transferred from gels to 0.2 µm of the polyvinylidene difluoride (PVDF) blotting membrane (GE healthcare life sciences, 2.5 h, 0.3 mA at 4 °C). Membranes were incubated with 5% fat-free milk for 1 h at room temperature to block nonspecific backgrounds then treated with primary antibodies overnight at 4 °C as follows: Iba-1 (Invitrogen, Carlsbad, CA, USA, cat # GT10312; 1:500 dilution), GFAP (Proteintech, Rosemont, IL, USA, cat # 16825-1-AP; 1:5000), nNOS (Millipore, Burlington, MA, USA, cat # 07-571-I; 1:1000), and iNOS (Abcam, Cambridge, UK, Cat # ab3523; 1:1000). The following day, the membranes were incubated with the appropriate HRP-conjugated secondary antibody at room temperature. The membranes were reprobed with antibodies against β-actin (Santa Cruz Biotechnology, Dallas, TX, USA, cat # sc-47778; 1:5000) or GAPDH (Santa Cruz Biotechnology, cat # sc-32233; 1:1000) to ensure loading consistency. Analyses were performed using Fiji (NIH, public domain), and data were expressed as the percentage of the intensity of the target protein to that corresponding to the loading control. All original blots used in this study can be found online in the Supplementary Materials.

### 4.5. Enzyme-Linked Immunosorbent Assay (ELISA)

To detect the expression of pro-inflammatory and anti-inflammatory cytokines, rats (N = 6 from each group) were euthanized after three days post-PVD, and the hippocampal tissue lysates were prepared as described above. The concentrations of IL-4, TNF-α, and TGF-β were calculated using ELISA kits for rat anti-rabbit antibodies of each cytokine (Thermofisher Scientific, Waltham, MA, USA). Samples were prediluted (1:10) based on an initial trial to find the appropriate dilution factor that validates the standard curve of the purchased ELISA kits. All samples were run in duplicates.

### 4.6. Propidium Iodide Staining

Propidium iodide (PI) is a fluorescent intercalating agent that is used effectively as a marker for cell death and the evaluation of cell viability since PI cannot cross the cell membrane of intact healthy cells, as it can only enter and label cells with disrupted plasma membranes and produces strong red fluorescence when excited by green light. Propidium iodide staining and the subsequent confocal imaging of rat hippocampal slices were used to examine the effect of istradefylline administration on cell survival after ischemic stroke induced by PVD surgery. The methods used were adapted from previous studies [22,23,24,32]. Following the equilibration of hippocampal slices for 1 h after slicing as described above, slices were incubated in fresh oxygenated aCSF at room temperature for 2 h. Then, 5 μg/mL propidium iodide (Sigma) was added to the aCSF, and slices were incubated for 1 h. Following the incubation period, slices were rinsed thoroughly in aCSF and then fixed in 4% paraformaldehyde at 4 °C overnight. The following day, slices were washed 3 × 15 min in 1X PBS and then mounted on glass microscope slides (VWR) and sealed using Prolong Gold Antifade Reagent (Invitrogen). After the addition of PI, all subsequent procedures were performed in the dark to prevent photobleaching.

Hippocampal slices were imaged using a Zeiss LSM700 laser scanning confocal microscope (Carl Zeiss Group, Toronto, ON, Canada) using green light (543 nm) to induce PI fluorescence. The whole hippocampus was imaged in pieces using a 10× objective lens, and images of CA1 pyramidal neurons were obtained using the Zeiss Plan-Apochromat 63×/1.6 oil-immersion objective lens (Carl Zeiss). CA1 images were acquired as Z-stack images of 200 μm depth into the hippocampal slice, with each Z-stack image taken at 2 μm. Two Z-stack images were taken along CA1 for each slice and were averaged using densitometry analysis. Data was collected using Zeiss Zen 2009 version 5.5 software (Carl Zeiss) and was analyzed using ImageJ (Public domain, Version 1.54p). Z-stack images closest to the outer top and bottom of the hippocampal slices were not analyzed, as the neuronal damage in those areas was enhanced by the slicing procedure. The inner-most 20 μm (~100 μm into the slice) segments were combined as maximum intensity projections, and intensities were compared between treatment groups using densitometry analysis. Collected densitometry data was normalized to a representative hippocampal slice from the sham group. Data was graphed as a percentage of the sham value and analyzed for significance against this control value (100%). Full hippocampal images were assembled as montages of the entire hippocampal slice using Adobe Photoshop CS6 (Adobe Systems, Mountain View, CA, USA).

### 4.7. FluoroJade C Staining

FluoroJade C (FJC) is a polyanionic fluorescein derivative that can be used as a sensitive and selective marker for degenerating neurons. Consequently, we used FJC to quantify the level of neurodegeneration in the hippocampus after inducing ischemic stroke. On the fourth day after PVD surgery, rat brains were obtained and sectioned as previously described [36]. In brief, anesthetized rats were intracardially perfused with 4% PFA in PBS for 30 min. After perfusion, brains were removed and fixed with 4% PFA in PBS overnight. Brains were then stored in 30% sucrose (*w*/*v*) in 0.1 M PBS for an additional 3 days to ensure cryoprotection before slicing. The brains were then frozen in liquid nitrogen in a Tissue-Tek OCT mounting medium, and 40 µm coronal sections of the hippocampus were cut with a cryostat. To visualize degenerative neurons, brain sections were mounted on gelatin-coated slides, air-dried on a slide warmer at 45 °C for 20 min., and subjected to FJC staining. The slides were first immersed in a solution containing 1% NaOH in 80% ethanol for 5 min, followed by 2 min rinses in 70% ethanol and then in distilled water. Brain slices were then incubated in 0.06% freshly prepared potassium permanganate solution for 10 min. Following a 2 min rinse in distilled water, slides were transferred to the 0.0001% FJC staining solution and stained for 10 min on a mechanical shaker to ensure uniform staining of the slices. The proper dilution was accomplished by first making a 0.01% stock solution of FJC dye (Millipore) in distilled water and then adding 1 mL of the stock solution to 99 mL of 0.1% acetic acid. The working diluted solution of FJC was used within 2 h of preparation, while the stock solution was stored at −20 °C and used within 3 months [36]. Slides were washed three times each for 1 min in distilled water and then air-dried on a slide warmer at 50 °C for 30 min. Then, slides were rinsed in xylene, and coverslips were mounted using Prolong Gold Antifade Reagent (Invitrogen). Digital images were obtained with Zeiss LSM 700 (Carl Zeiss) using a 10× objective for the hippocampal montages and a 63×/1.4 oil-immersion objective lens for the magnified regions of the hippocampal pyramidal body layers. Two Z-stack images of the CA1 region (taken at 1 μm intervals) were averaged using a similar densitometry analysis performed with the PI analysis above. Collected densitometry data was normalized to the representative hippocampal slice from the sham group. Data was graphed as a percentage of the sham value and analyzed for significance against this control value (100%). Full hippocampal images were assembled as montages of the entire hippocampal slice using Adobe Photoshop CS6 (Adobe Systems, Mountain View, CA, USA).

### 4.8. Pial Vessel Disruption as a Model of Small-Vessel Stroke

Disruption of class II-sized vessels on the surface of the cortex (pia), known as pial vessel disruption (PVD), has been shown to induce a small focal cortical lesion, which, within 3 weeks, forms a lacuna-like fluid-filled cyst surrounded by a barrier rich with reactive astrocytes [36,91,92]. Using histological staining, the PVD stroke model induces an approximately 1 mm^3^ permanent, non-reperfusion lesion, which is confined to the cortex and does not extend into the underlying corpus callosum [91,92,93]. PVD surgery, which is described briefly below, has been modified by our lab and extensively studied as a small-vessel in vivo animal stroke model since it has several advantages over other stroke models [21,36]. For instance, this PVD model is a non-perfusion small-vessel-stroke model that produces permanent damage to class II-sized vessels, and the cortical lesion volumes of approximately 1 mm^3^ can be reliably reproduced and closely resemble a lacunar infarction [91,93]. On the contrary, most of the focal or global animal stroke models used by other groups are ischemic/reperfusion models that involve the transient occlusion of large vessels, such as the middle cerebral artery occlusion (MCAO) model [94,95], and the cerebral ischemic damage often encompasses large volumes of brain regions.

In brief, PVD surgery was performed as follows: Male *Sprague-Dawley* rats weighing approximately 250 g received 2% isoflurane for the induction of anesthesia, and then the hair of the skull was shaved. Rats were kept immobile by transferring them to a stereotaxic frame with a temperature-controlled heating pad connected to a rectal thermoprobe to monitor and maintain their body temperature throughout the surgery at 37 °C. Anesthesia was maintained by 2% isoflurane delivered through a stereotaxic frame throughout the surgery, and then rats were subcutaneously injected with Buprenorphine (0.035 mg/kg) for pain management. A craniotomy was performed with a 5 mm diameter trephine positioned on the right and rostral side of the bregma adjacent to the coronal and sagittal sutures. Cool sterile saline was applied intermittently to prevent overheating from the high-speed drilling. After removal of the dura and exposing the cortical surface and the overlying pial vessels, medium-sized (class II) pial vessels were disrupted by fine-tipped forceps. The piece of bone was placed back, and the scalp was then closed with a wound clip. Sham animals received the same treatment with dura removal but no vessel disruption. Animals were kept in a cage separately under a warm lamp during the recovery from anesthesia, and thereafter, the animals were returned to their cages.

### 4.9. Y-Maze

Rats were transported to the behavior room at least 1 h before starting any behavior tasks to allow for acclimatization. The Y-maze apparatus consists of three arms that are joined by a triangle-shaped center to form a “Y” shape. Each arm has a rectangular base that is 45 × 12 cm^2^, and all areas of the maze are surrounded by 35 cm tall walls. The task consisted of two trials separated by a 90 min interval. In the first trial (acquisition), one of the three arms was blocked (novel arm), and then rats were placed in the maze facing the end of a randomly chosen arm (start arm). The trial was 15 min long, during which rats were free to explore the start arm and the old arm, but not the novel arm of the maze. After the first trial, rats were placed back in their cages for a 90 min break. In the second trial (retrieval), rats were placed in the maze for 5 min and were free to explore all arms, including the novel arm that was blocked in the first trial. For both trials, there were visual spatial cues on the walls outside the maze for rats to easily view throughout their exploration periods. A video camera was used to record both trials. The video-tracking software, EthoVisionXT Version 12 (Noldus, Leesburg, VA, USA), was used in a single-blinded way to automatically score time spent in each arm in seconds (s). These scores were determined for the second 5 min retrieval trial. Time spent in each of the three arms was calculated as a percentage of the total trial time.

### 4.10. Open Field Test

The open-field-test apparatus is a 56 × 56 cm^2^ square-shaped field surrounded by 57 cm tall walls. The bright light that shines directly above the maze causes the 28 × 28 cm^2^ center square to be the brightest and most exposed area of the field. Rats were initially placed in this center square and were free to explore the entirety of the field for 15 min. A video camera was used to record their behavior. Since rodents have the tendency to mainly explore the peripheral areas when placed in an open field, a phenomenon called thigmotaxis, the open field test was used as a validated behavioral test to assess the degree of anxiety that is directly related to the level of thigmotaxis exhibited by the rats in the open field [96,97]. EthoVisionXT (Noldus) was used to automatically score the following: center square entries and center square duration (s). Furthermore, heat maps for both Y-maze and open field tasks were obtained by EthoVisionXT (Noldus).

### 4.11. Forced Swim Test (FST)

The forced swim test (FST) is a behavioral test that measures depressive symptoms such as despair and learned helplessness. When rodents are first placed in water, they are expected to swim as vigorously as possible to escape this stressful situation [98]. However, as time passes, the animal reaches a point of helplessness and despair, which is reflected by immobility [98]. The FST was used to assess the depressive-like behavior of rats following PVD surgery and evaluate the potential of istradefylline to restore their mobility. The FST apparatus is a 30 × 30 × 60 cm^3^ rectangle-shaped acrylic glass container that is two-thirds filled with water at 25 °C [98]. Rats were placed in the container for 10 min, during which their behavior was recorded by a video camera. The FST was the last of the behavioral tests to be conducted on the third experimental day to prevent its stressor from affecting the other behavioral tests that were conducted.

The video files were uploaded to EthoVisionXT (Noldus), which automatically scored two activity statuses throughout the test. The highly active and inactive statuses were separated by a threshold of 0.15% of the total activity; then, the percentage of time spent immobile during the 10 min trial and the latency of immobility were calculated. Rats were also scored for both success and vigor as a function of the continuous movement of 4 limbs and swimming with their heads above water, respectively [34]. Criteria for success scores were as follows: 3, continuous movement of all four limbs; 2.5, occasional floating; 2, floating more than swimming; 1.5, occasional swimming using all four limbs; 1, occasional swimming using only hind limbs; 0, no use of limbs. Criteria for vigor scores were as follows: 3, entire head above water; 2.5, ears but not eyes are usually below water; 2, eyes but not nose are usually below water; 1.5, entire head below water for three seconds; 1, entire head below water for periods ≥ six seconds; 0, animal on the bottom of the tank for periods of ten seconds or longer. Trials were stopped and excluded if rats spent more than 20 s swimming at the bottom of the tank. A sum of scores for the last three minutes of the test was used to evaluate the immobility of rats undergoing different treatments. The treatment groups were double-blinded during the experiment and analysis.

### 4.12. Rotarod

The rotarod test was performed according to the method by Deacon [99] to assess for motor coordination and post-stroke motor deficits, with slight modifications. The four-chambered rotarod (Powermax II, 1.8° Step motor, Economex enclosure model) from Columbus Instruments, Ohio, USA, was used for the experiment. Rats were placed in the behavioral room 30 min prior to experimentation for habituation. Rats were pre-trained in the rotarod for 2 days before induction (two trials each day) and then tested 72 h following PVD surgery. During the pre-surgery rotarod baseline studies, all animals underwent rotarod habituation training for two days (two trials per day) prior to PVD surgeries or treatments. By the fourth trial, animals from each treatment group achieved latency times of falling of approximately 55 s. These results indicate that all animals acquired sufficient motor coordination and balance prior to treatment. Rats were placed on the rod, one in each chamber facing away from the direction of rotation, and then the rotation started with an accelerated rate of 5 rpm/s. The latency time of falling from the rod was recorded. The test was repeated three times, and the mean value for each animal was calculated. The maximum time and speed of the rotation were set as 120 s and 50 rpm, respectively. The apparatus was cleaned with 70% alcohol between each trial, and the observer who scored the experiment was blinded to the treatment groups.

### 4.13. Statistical Analysis

All graphs were constructed using GraphPad Prism 6.0 (GraphPad, La Jolla, CA, USA), where values were expressed as mean ± SEM for all treatment groups. Statistical significance was assessed using a one-way ANOVA test and a Tukey–Kramer multiple-comparison test with a 95% confidence interval using GraphPad InStat version 6.0 (GraphPad, La Jolla, CA, USA). The reported N values are obtained from independent experiments on the brains of different animals and are randomly selected. Probability values (*p*) of less than 0.05 were considered statistically significant. Behavioral analysis was performed independently in a single-blinded manner using EthoVisionXT (Noldus) software. Rotarod and FST vigor and success scores were manually scored in a single-blinded manner.

## Data Availability

The data that support the findings of this study are available upon request from the corresponding author.

## Supplementary Materials

The following supporting information can be downloaded at https://www.mdpi.com/article/10.3390/ijms26125680/s1.
