# Supplementary material for: The Anti-Parkinsonian A2A Receptor Antagonist Istradefylline (KW-6002) Attenuates Behavioral Abnormalities, Neuroinflammation, and Neurodegeneration in Cerebral Ischemia: An Adenosinergic Signaling Link Between Stroke and Parkinson’s Disease
Source: Int J Mol Sci. 2025 Jun 13;26(12):5680. doi: 10.3390/ijms26125680 (PMC12193193; doi:10.3390/ijms26125680)

MAX_SHAM2 FJC 63X CA1 ZSTACK shot 1

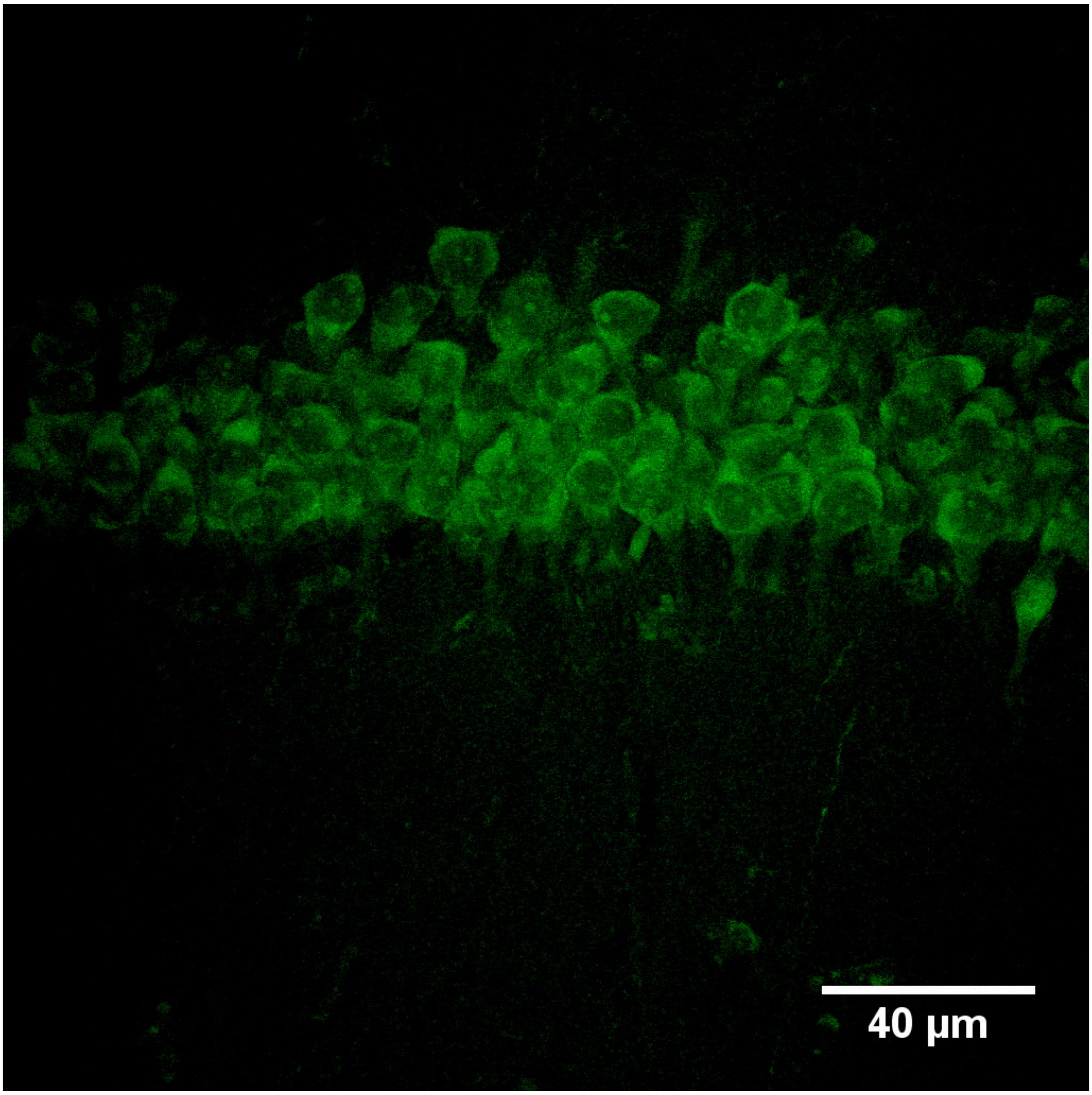

SHAM ipsi

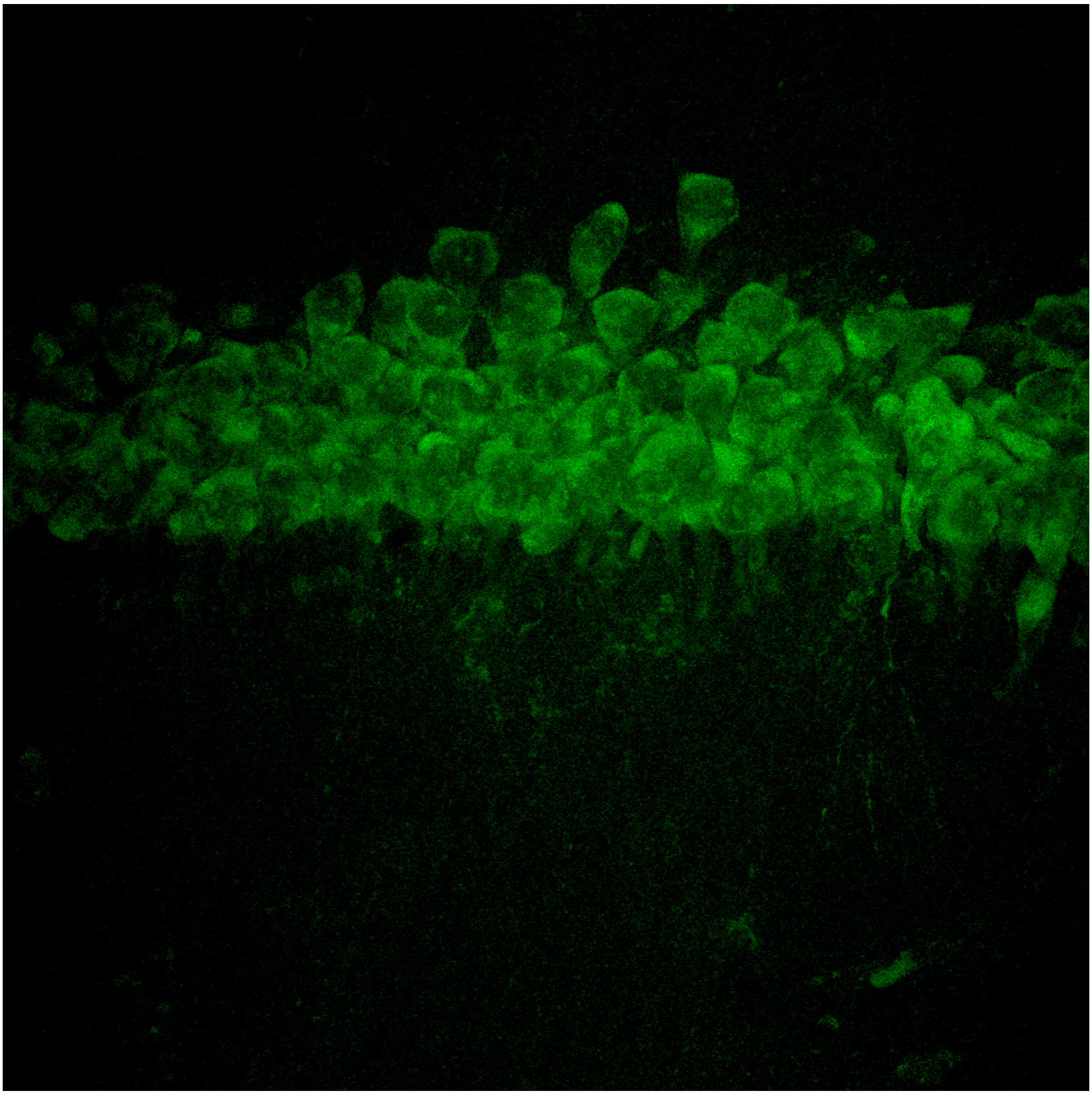

SHAM2 FJC 63X CA1 shot 1

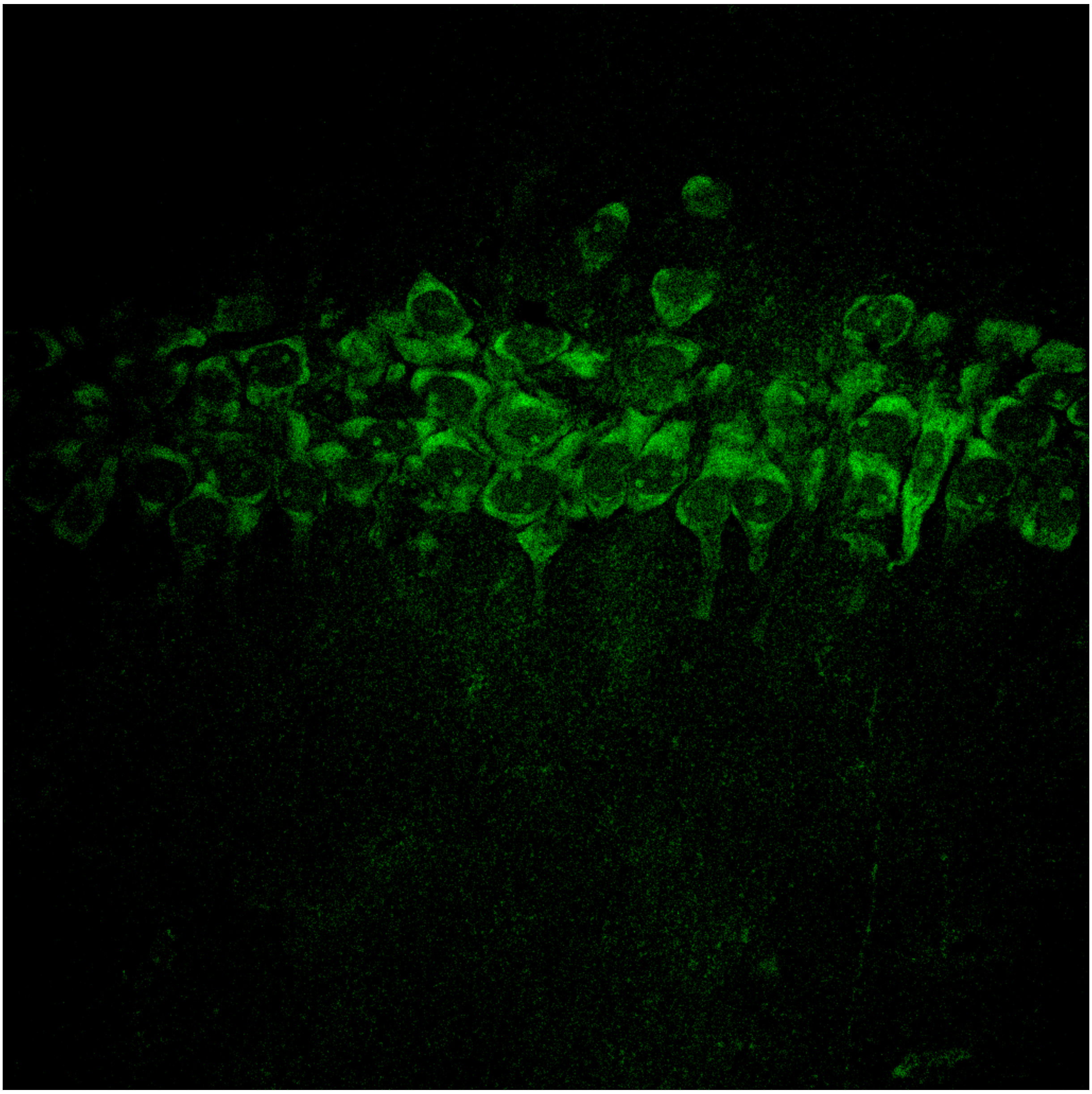

SHAM3 FJC 63X CA1 shot 1

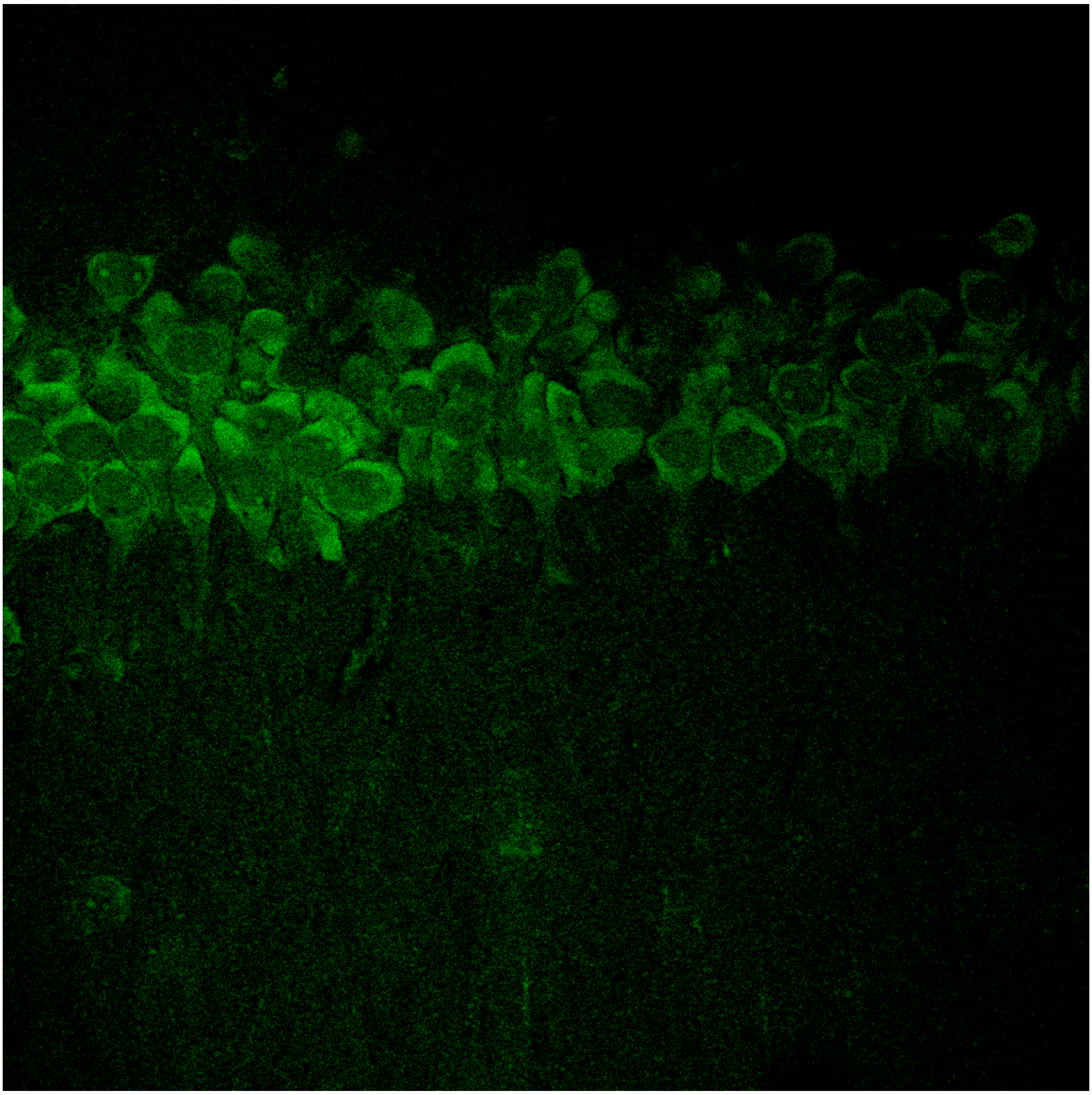

MAX_PVD2 FJC 63X CA1 ZSTACK shot 1

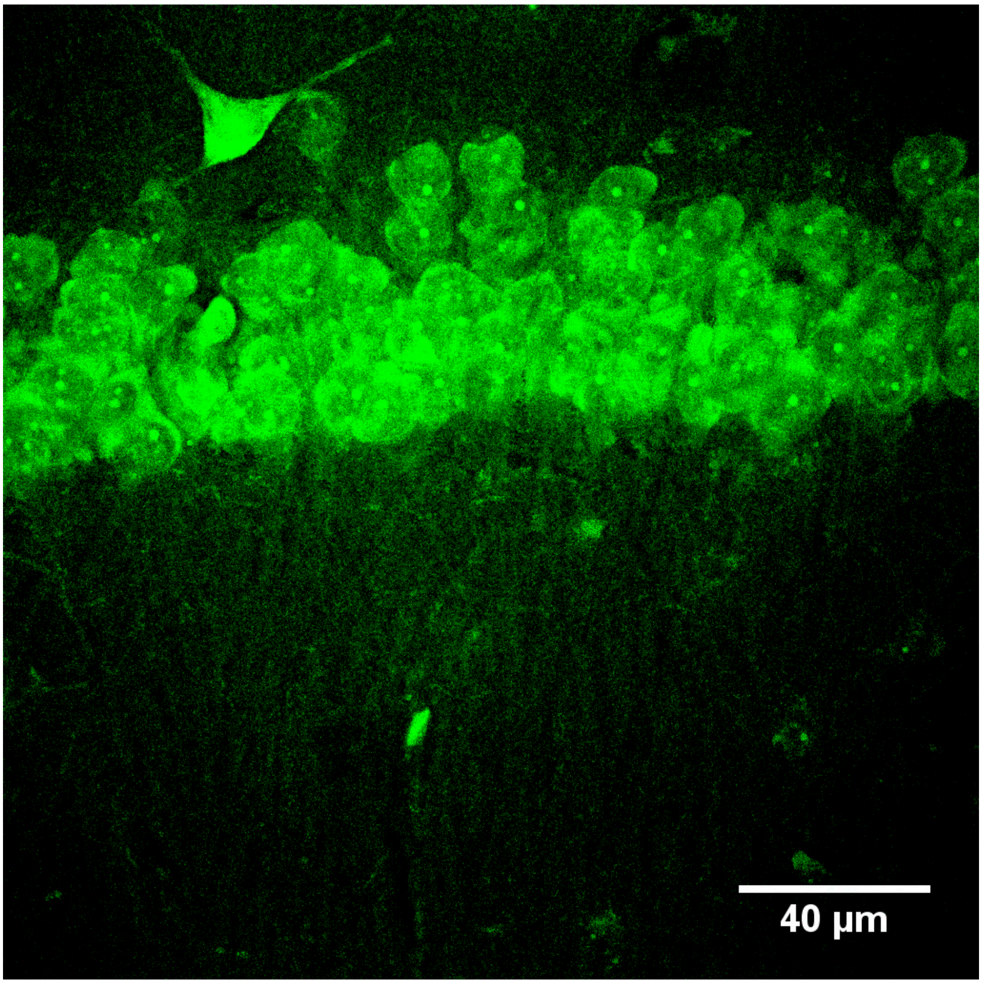

PVD2 IPSI

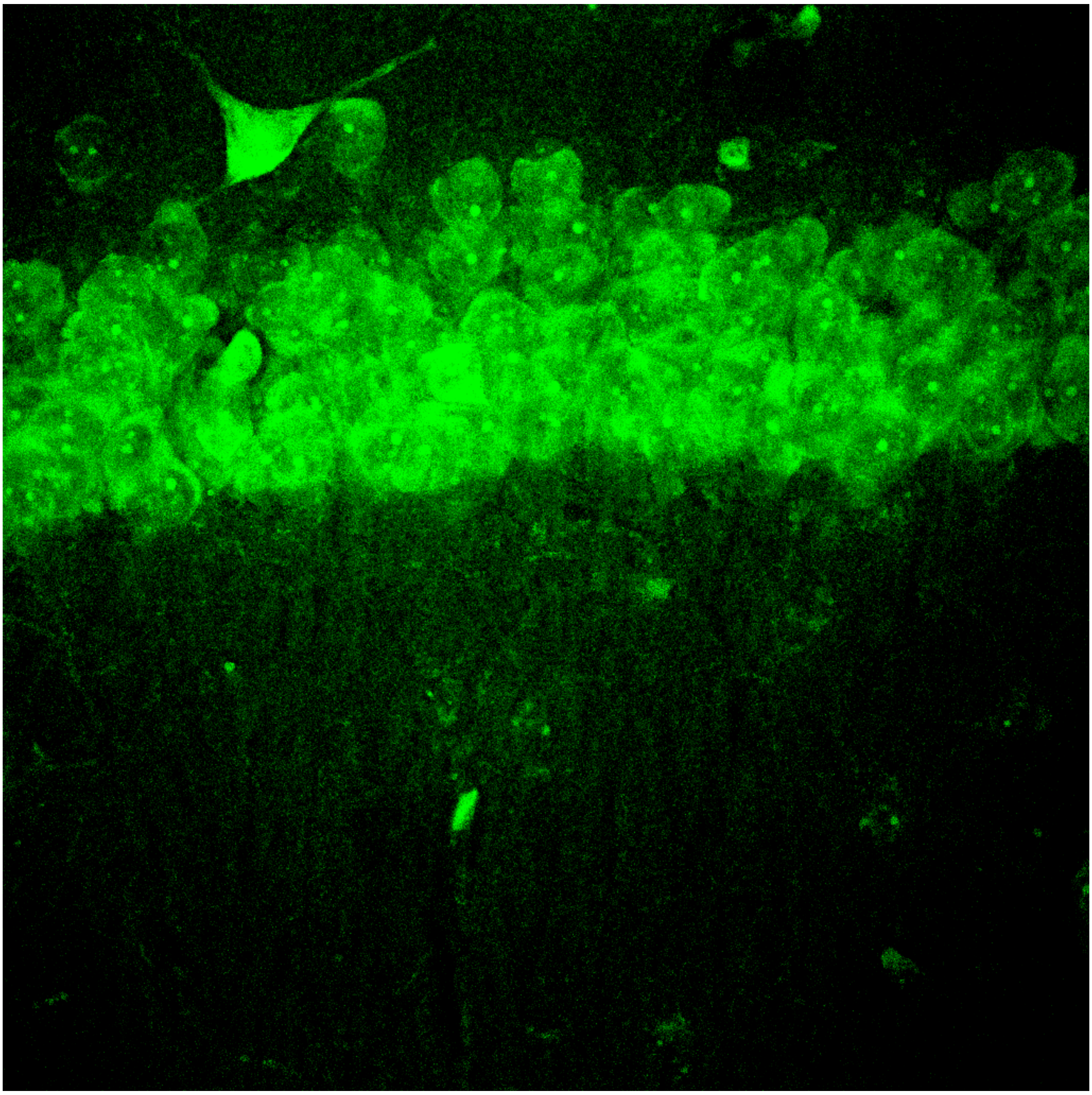

PVD2 + ISTRADEFYLLINE FJC 63X CA1 shot 1

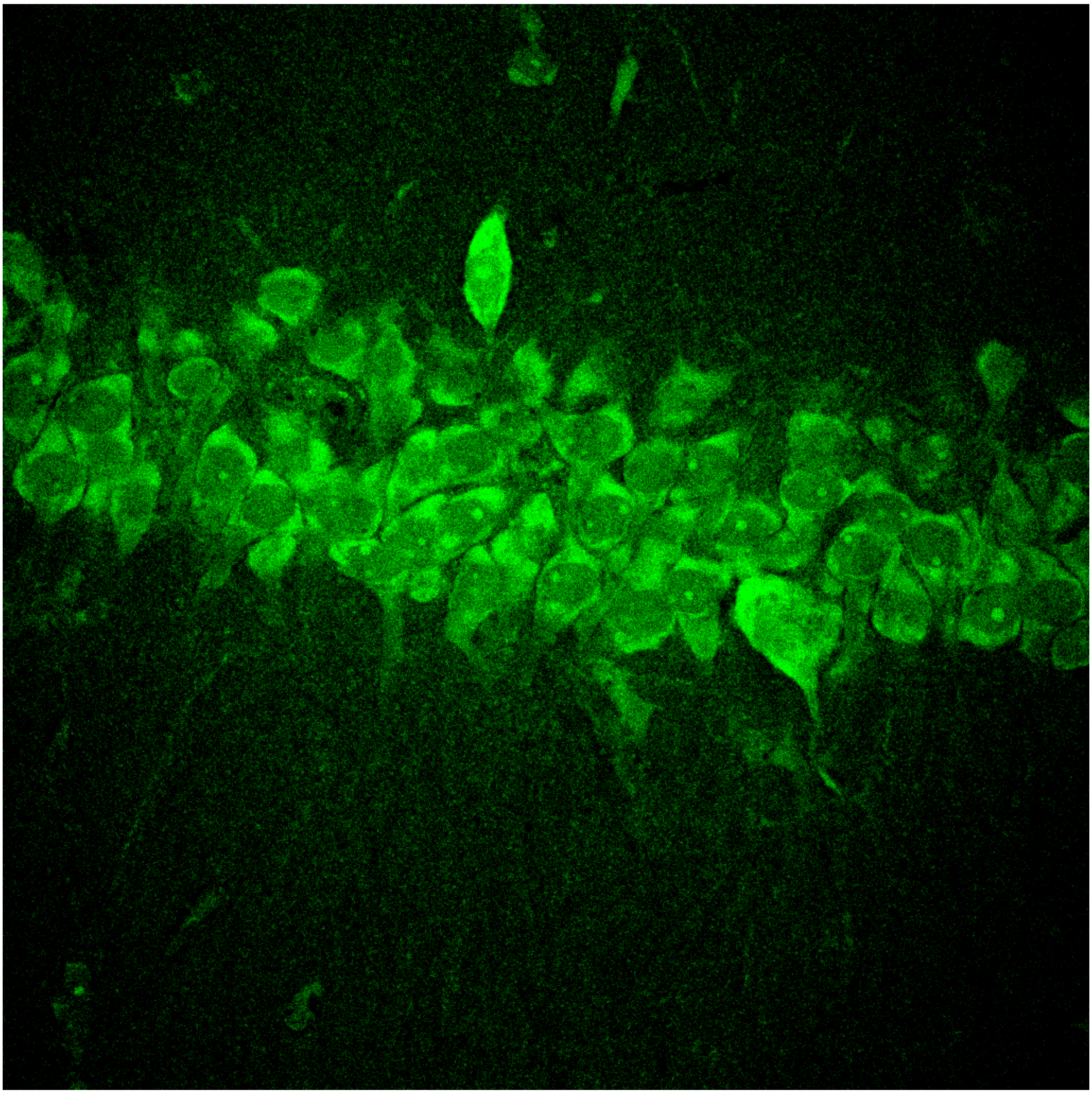

MAX_PVD3 + ISTRADEFYLLINE FJC 63X CA1 shot 1

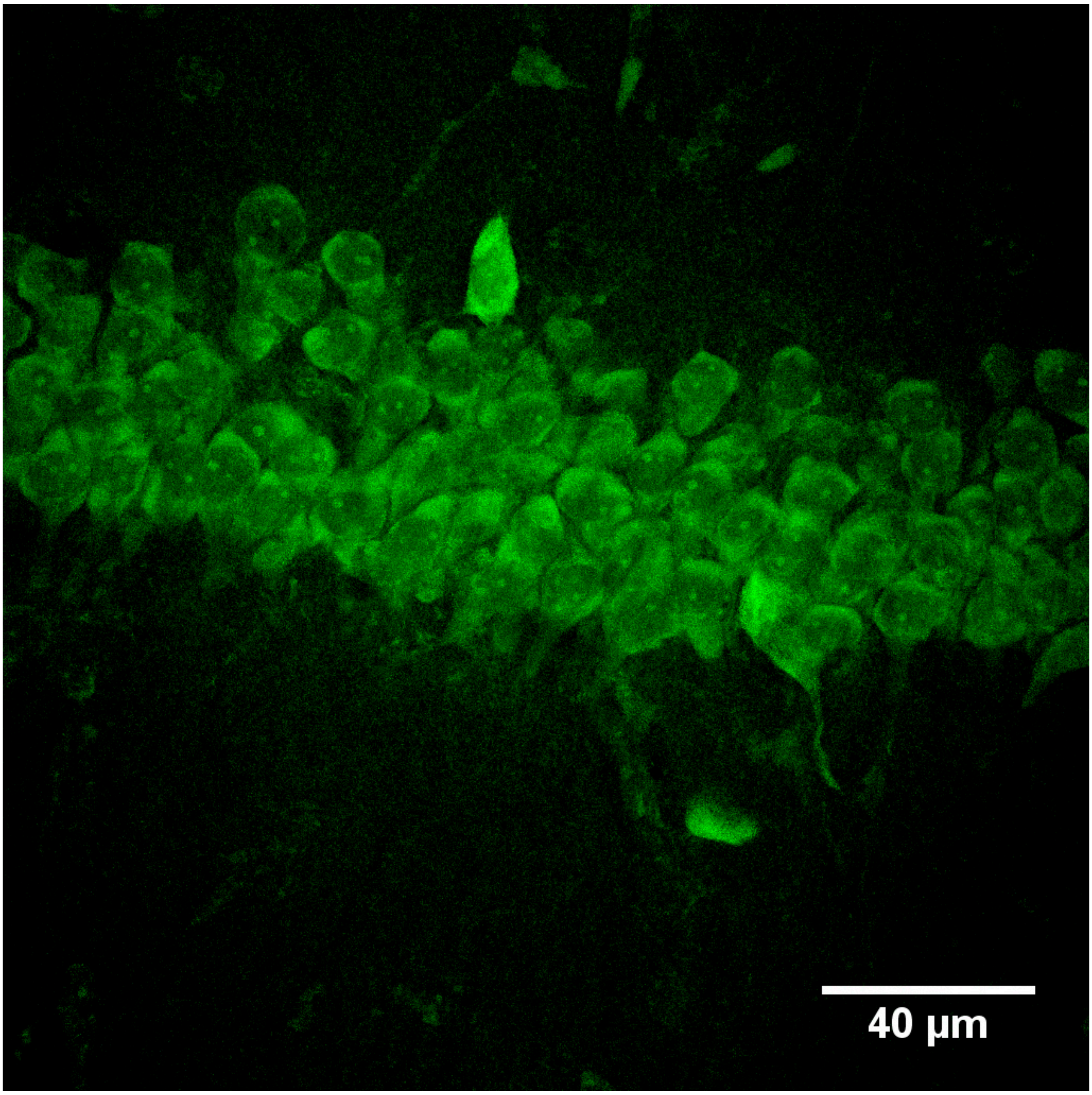

PVD3 + ISTRADEFYLLINE FJC 63X CA1 shot

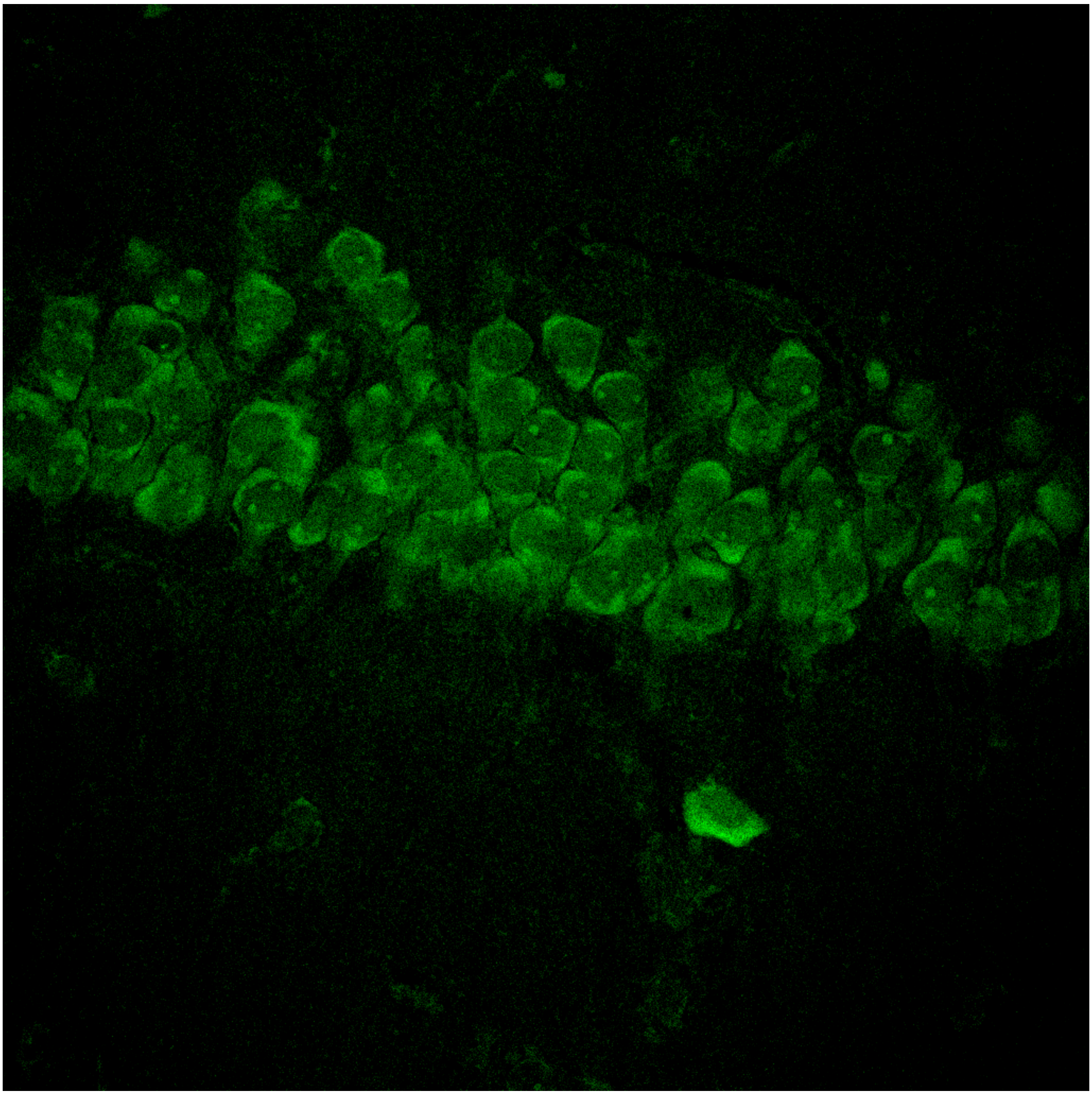

PVD+ISTRADEFYLLINE FJC 63X shot 3

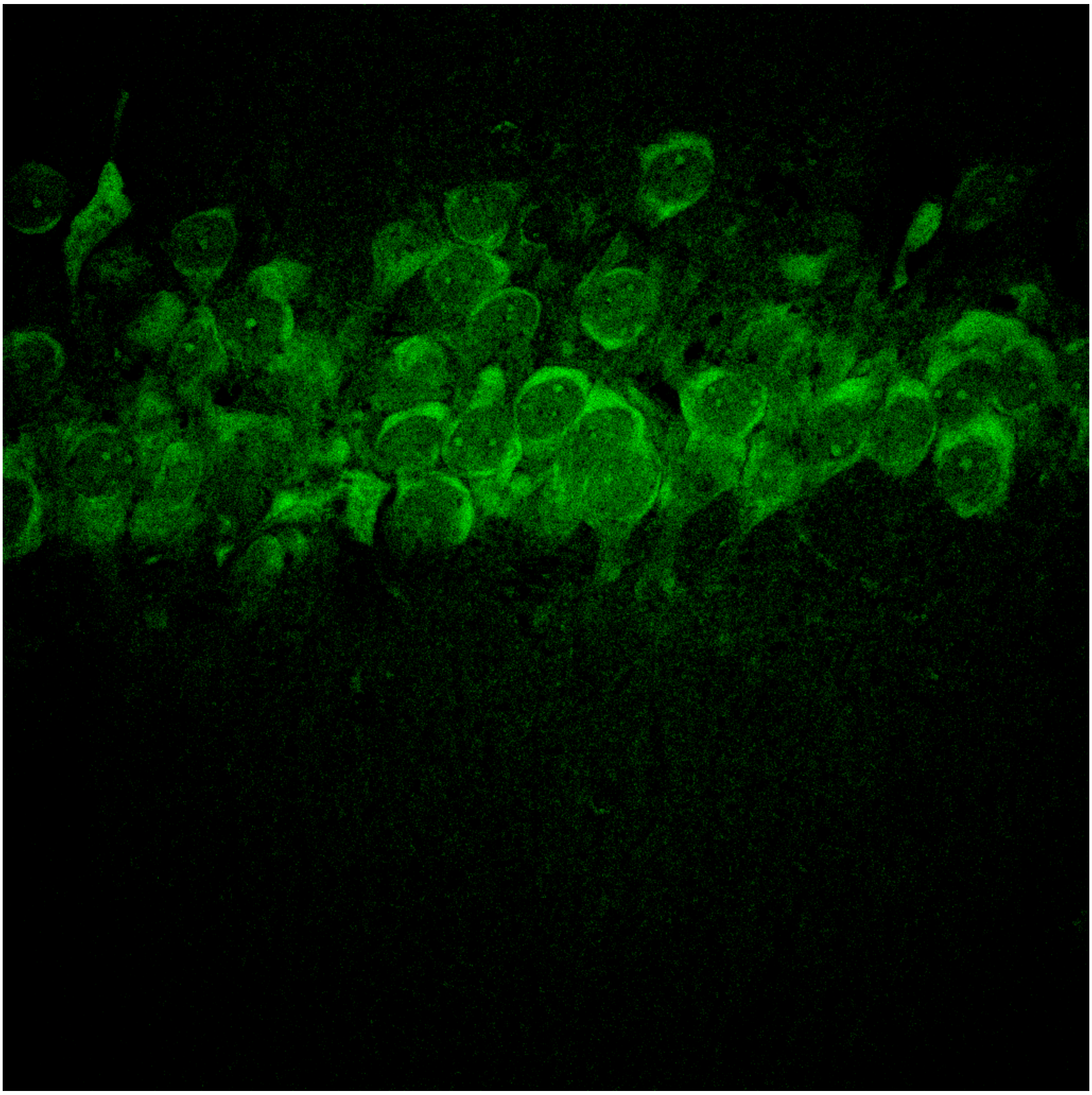

PVD+ISTRADEFYLLINE FJC 63X shot 1

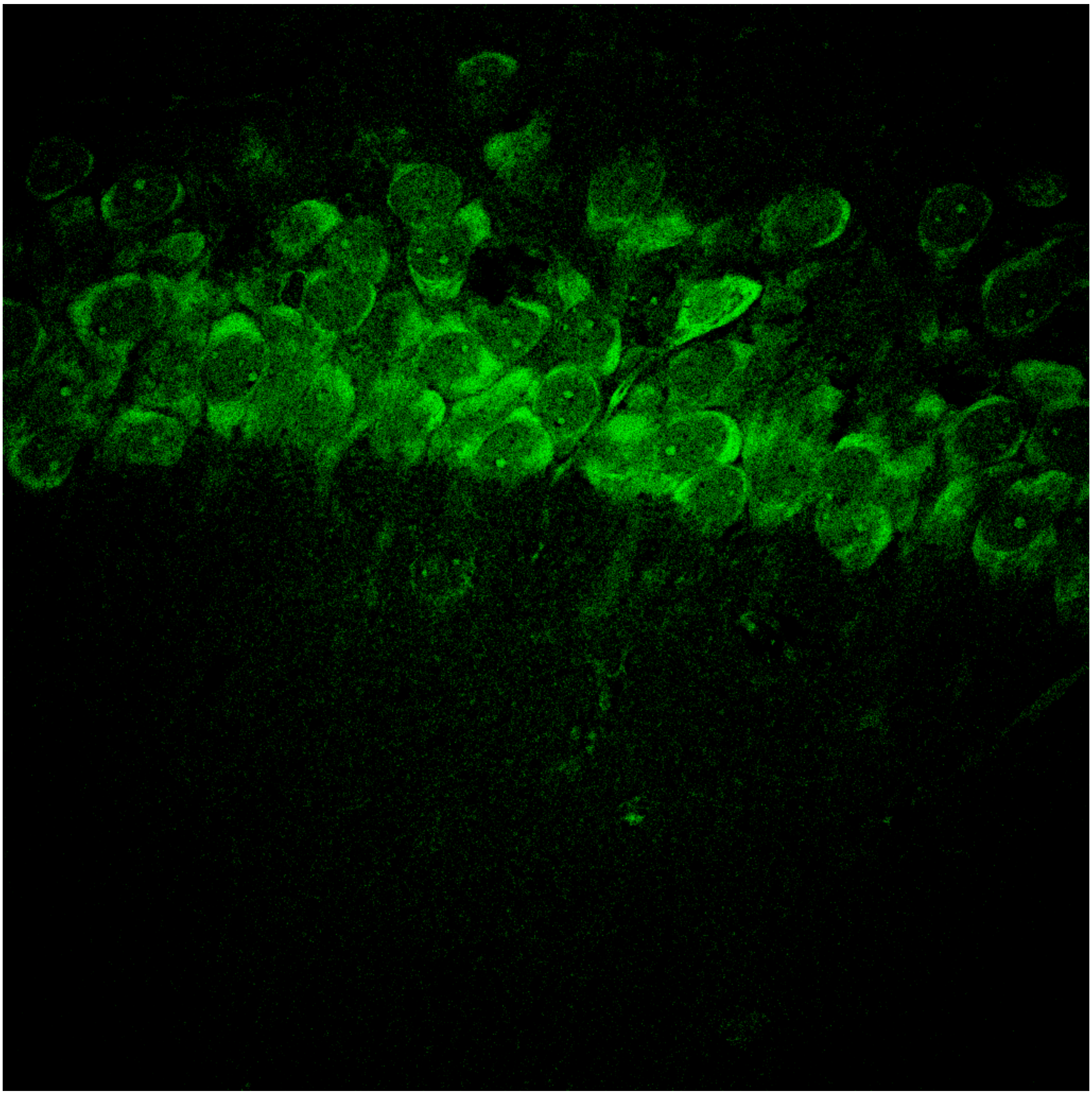

PVD+ISTRADEFYLLINE FJC 63X shot 2

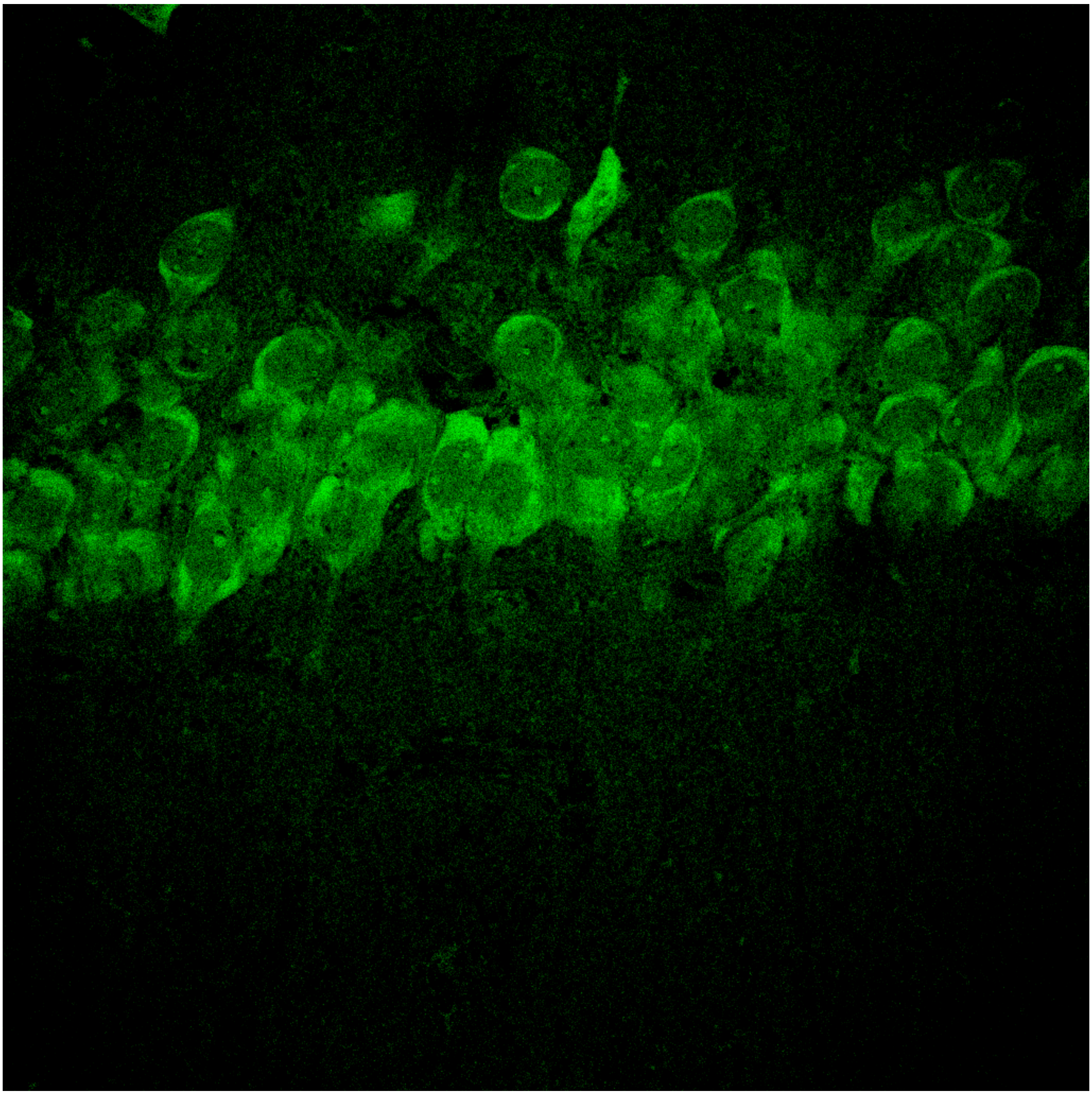

No Hypoxia PI 63X Hippocampus CA1 zstack image 3_Maximum intensity projection

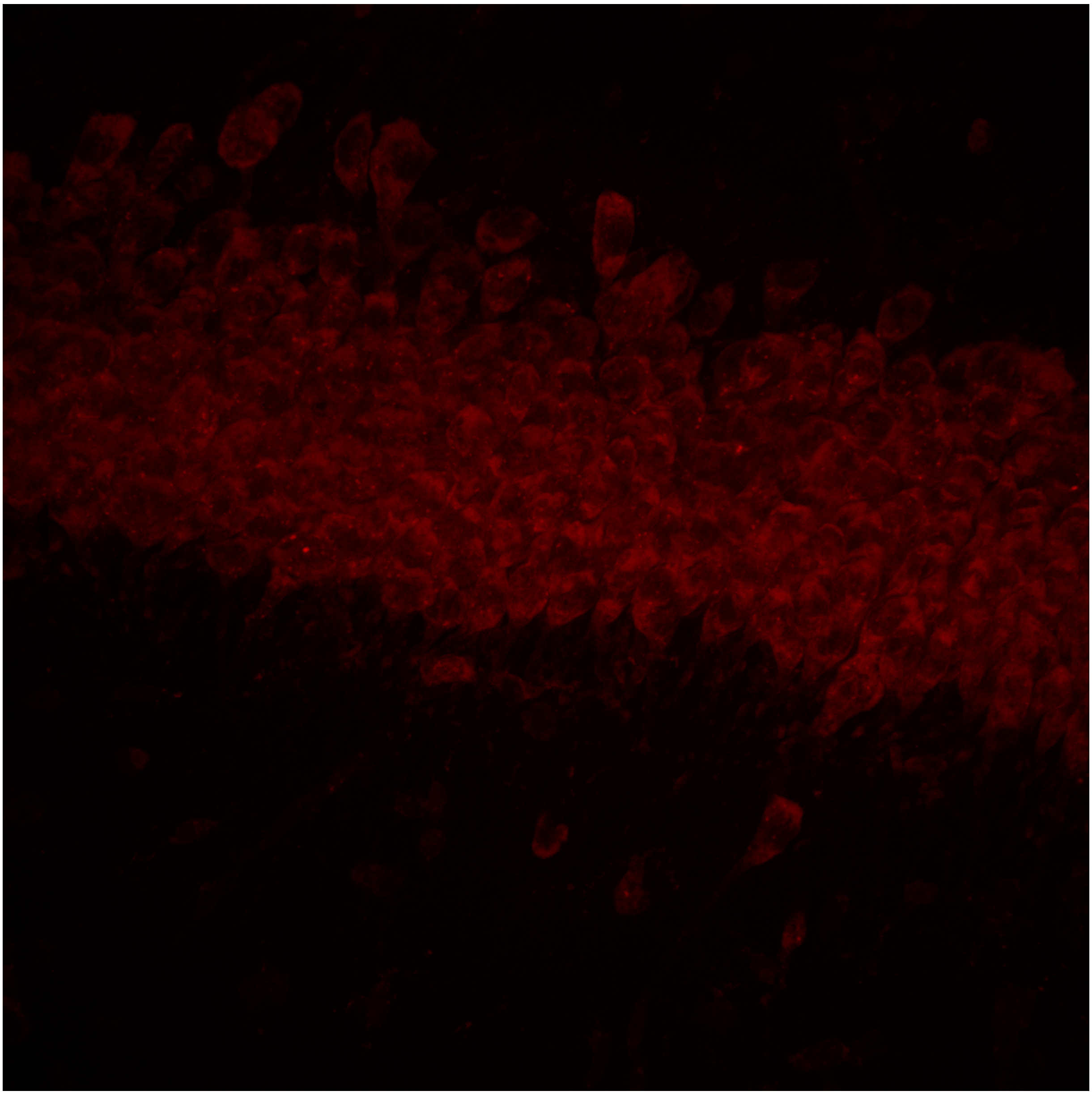

Hypoxia PI CA1 63X Zstack 3_Maximum intensity projection

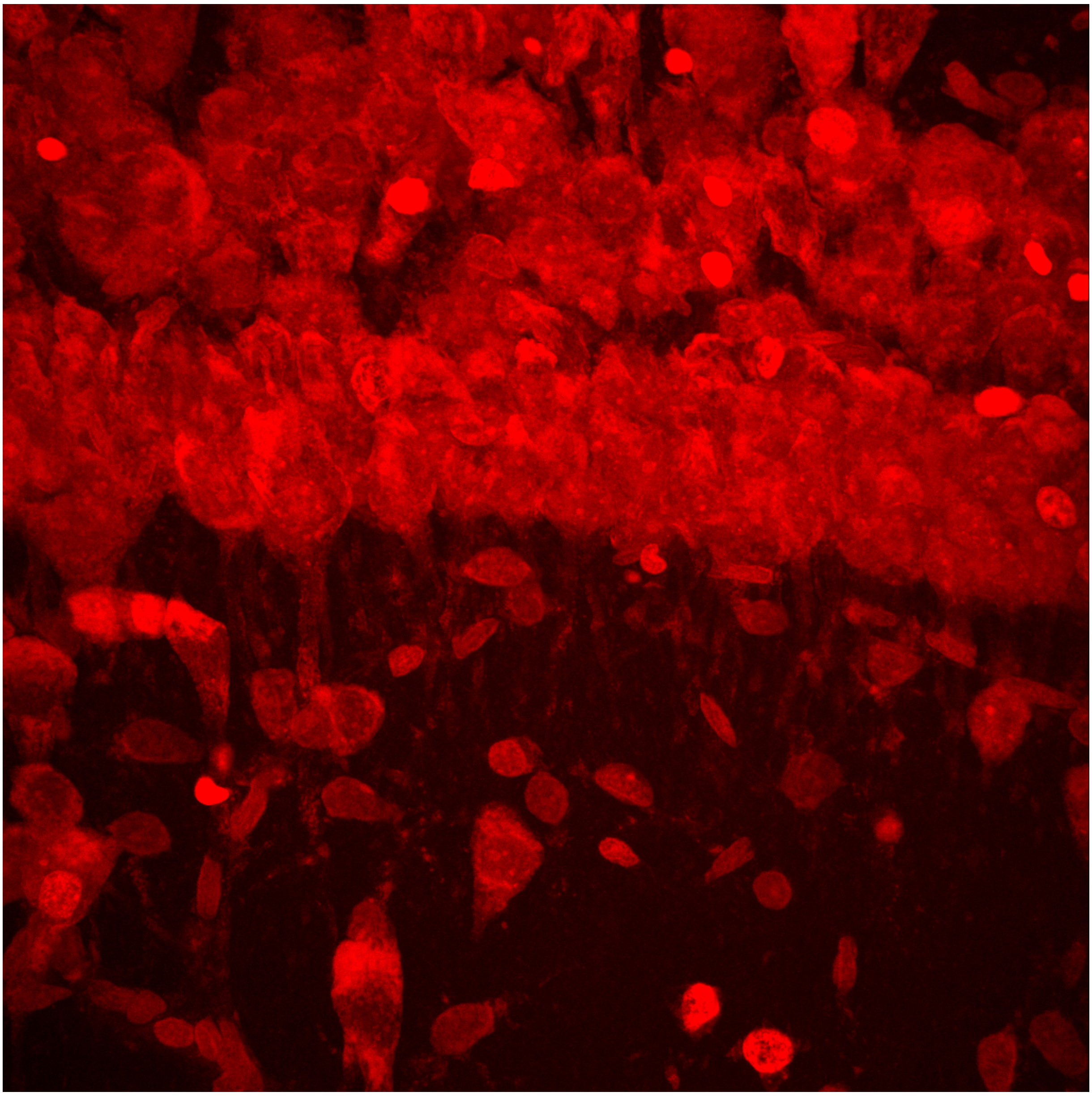

Istrad pretreated hypoxia reperfusion PI CA1 63X Zstack 5_Maximum intensity projection

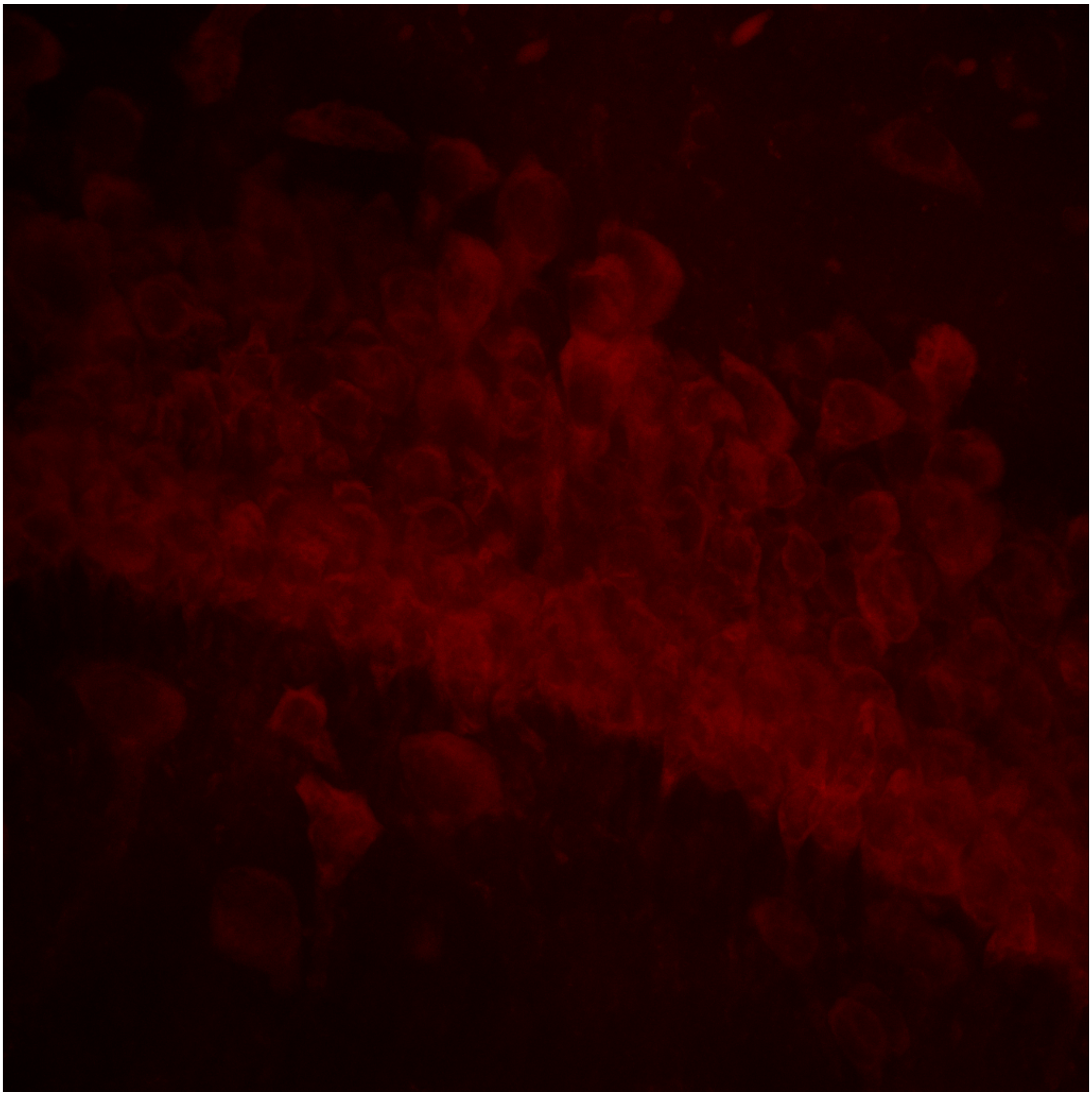

SHAM contralateral 63 X

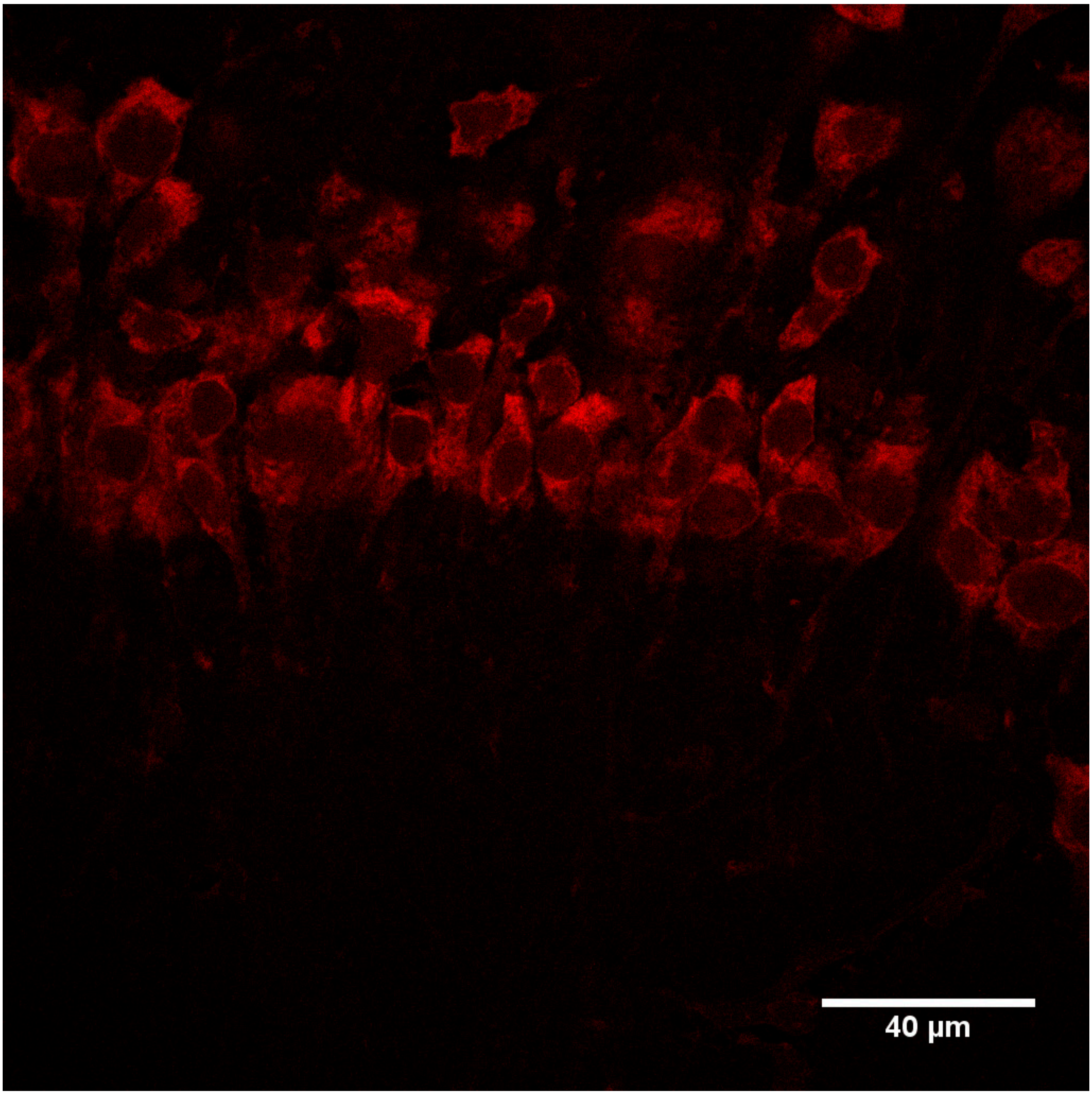

SHAM1 Ipsilateral 63 X shoot 1

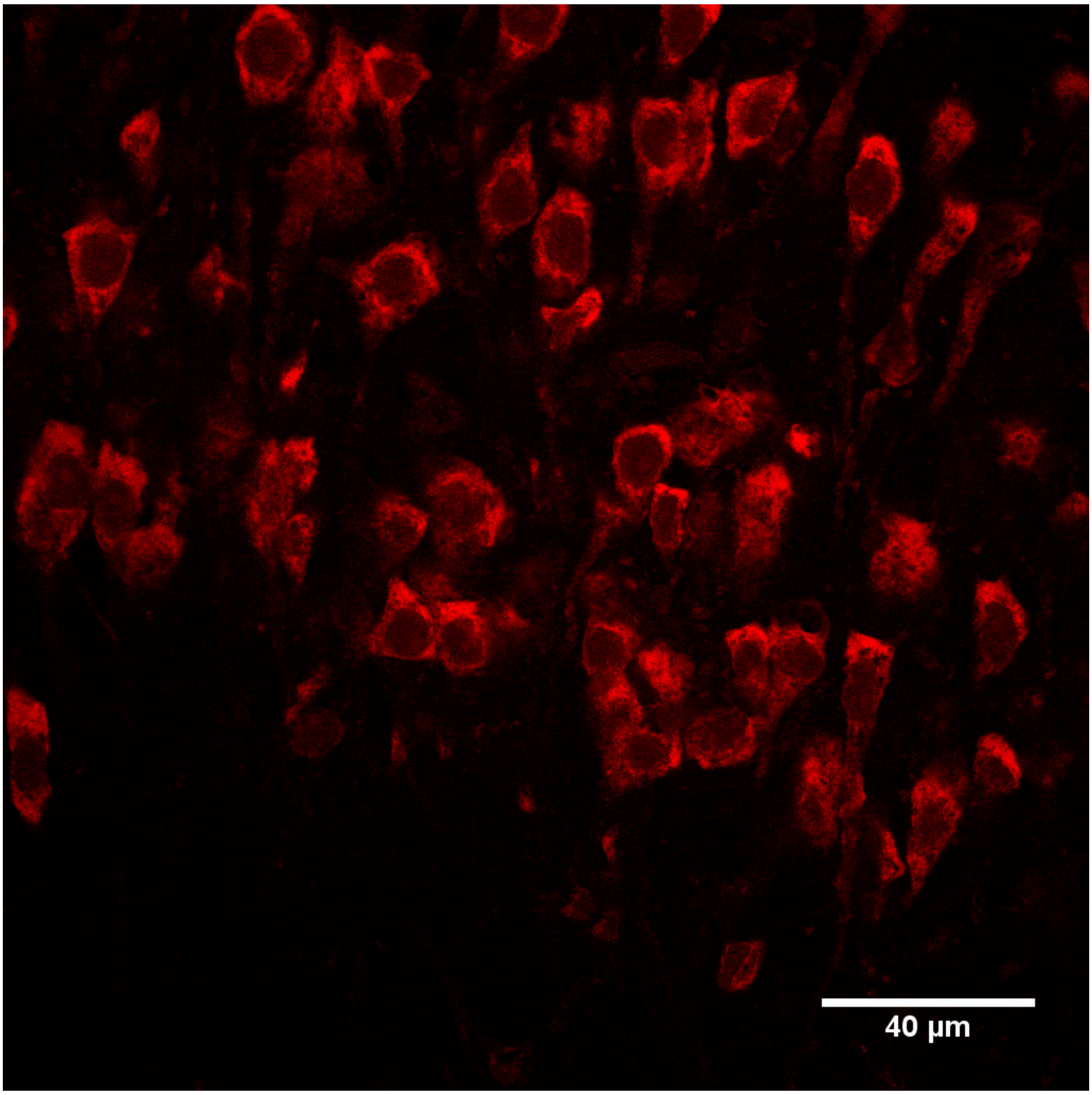

PVD Ipsilateral 63X

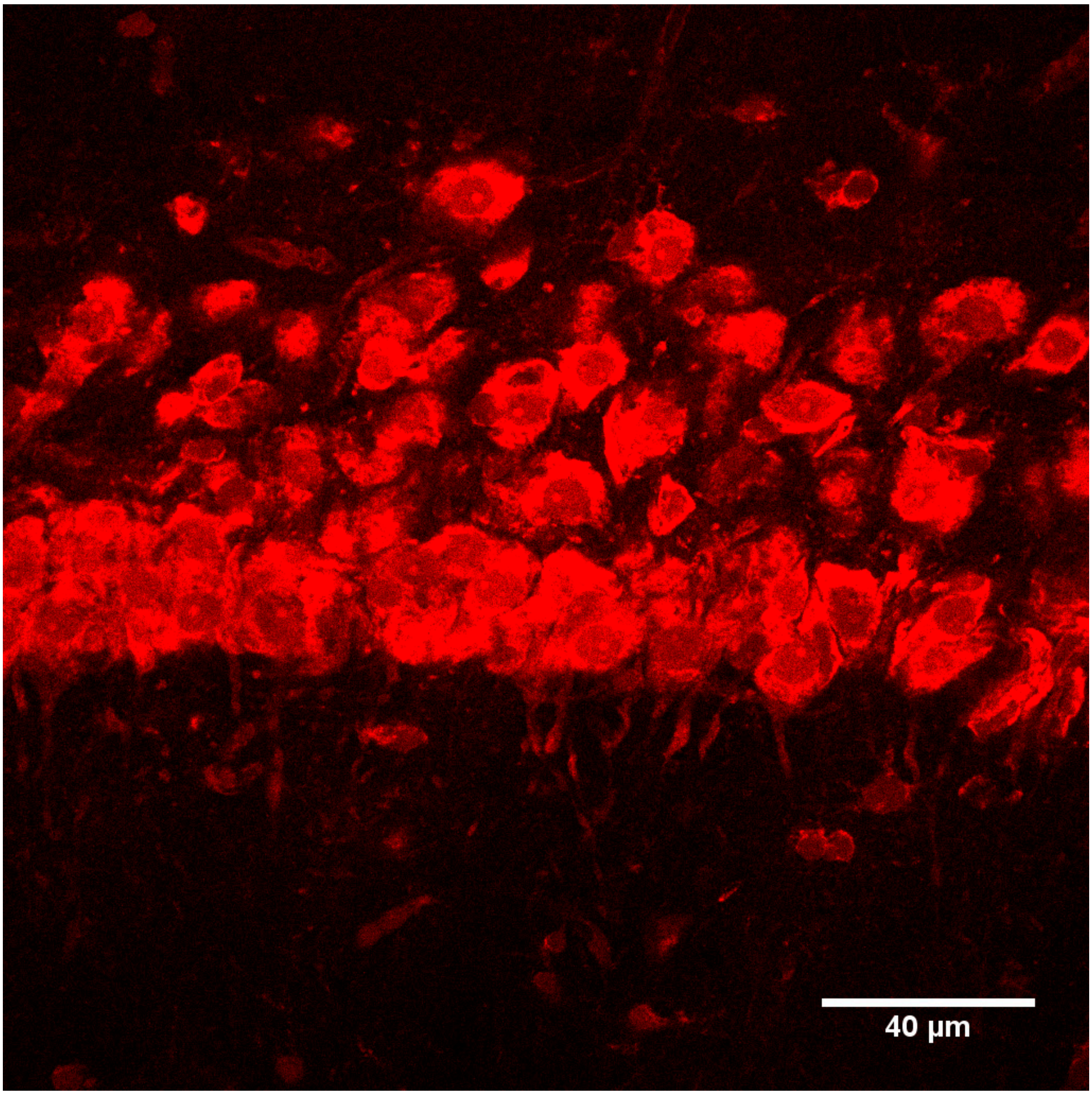

PVD1contrailateral63Xshoot1

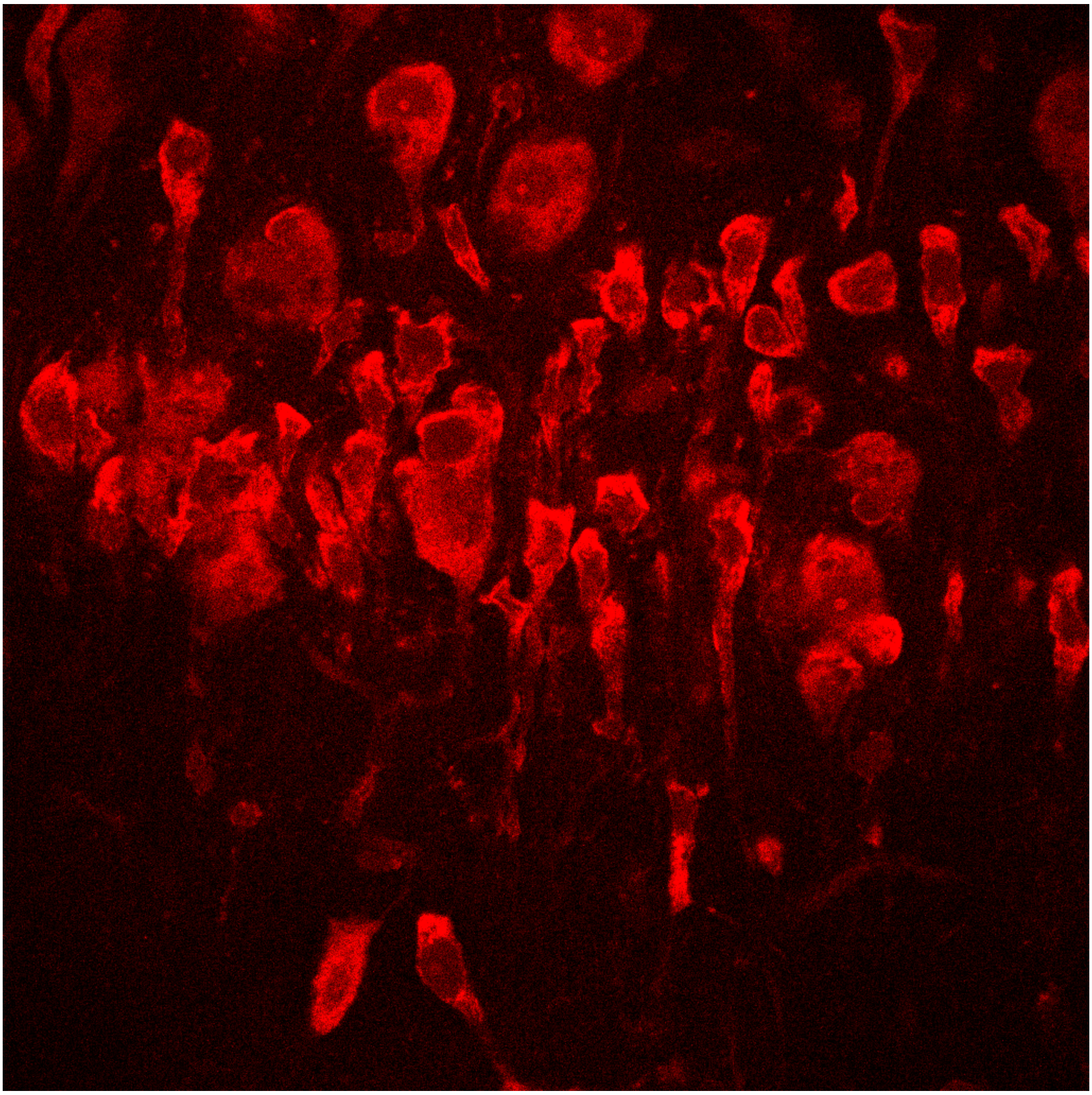

PVD1IpsilateralCA163Xshoot1

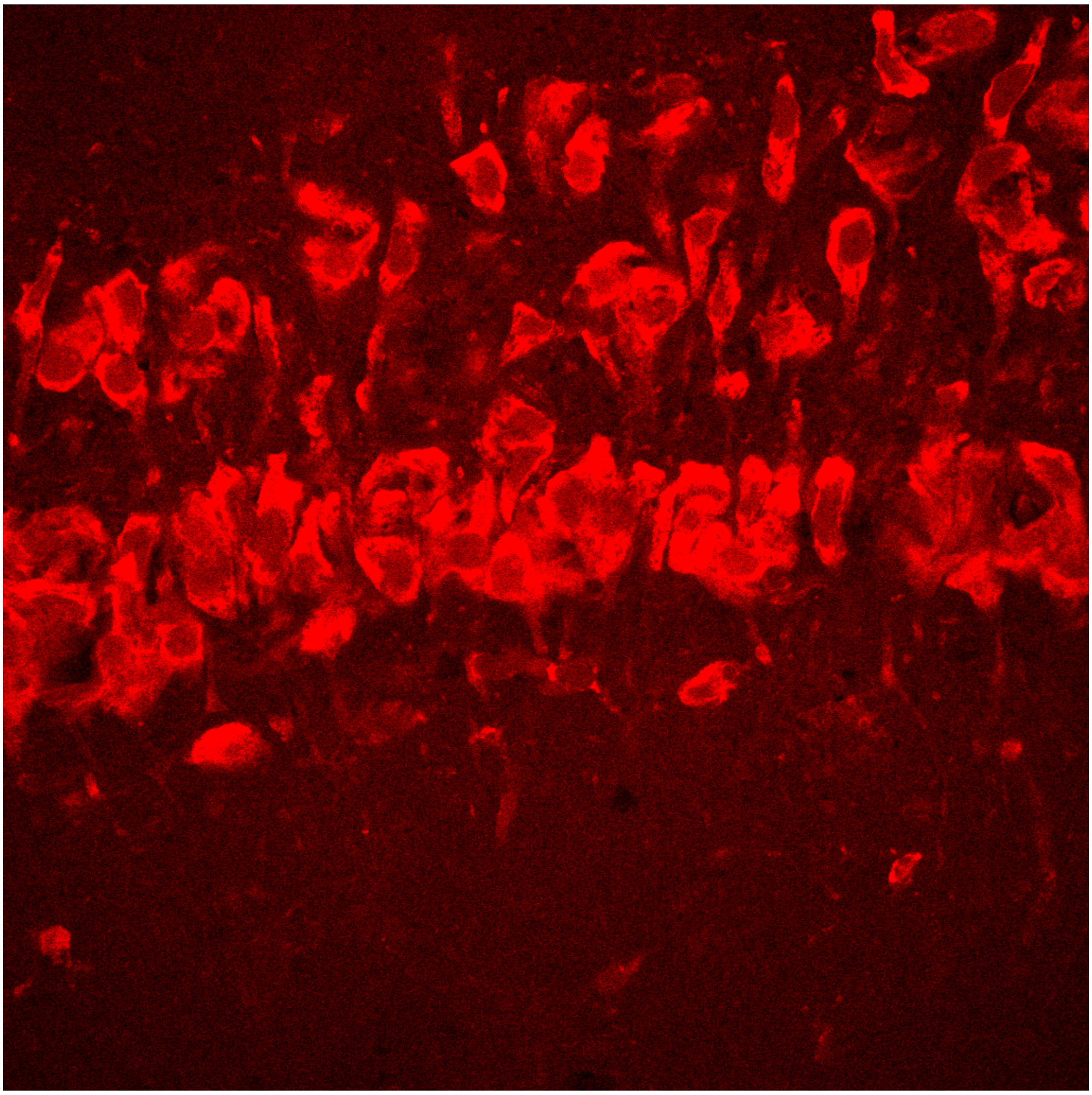

PVD2IpsilateralCA163Xshoot1

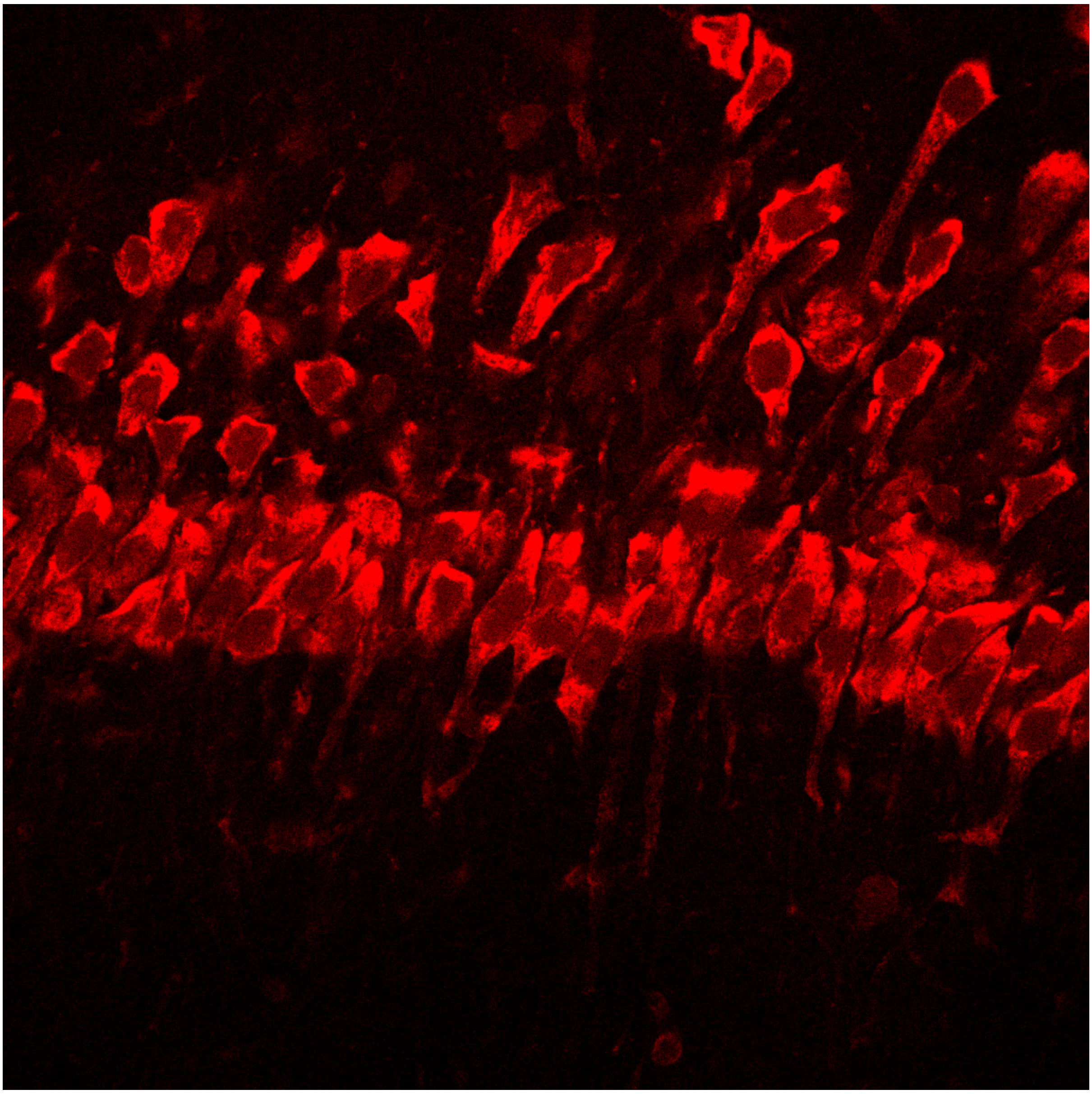

PVD2 Contralateral CA1 63X

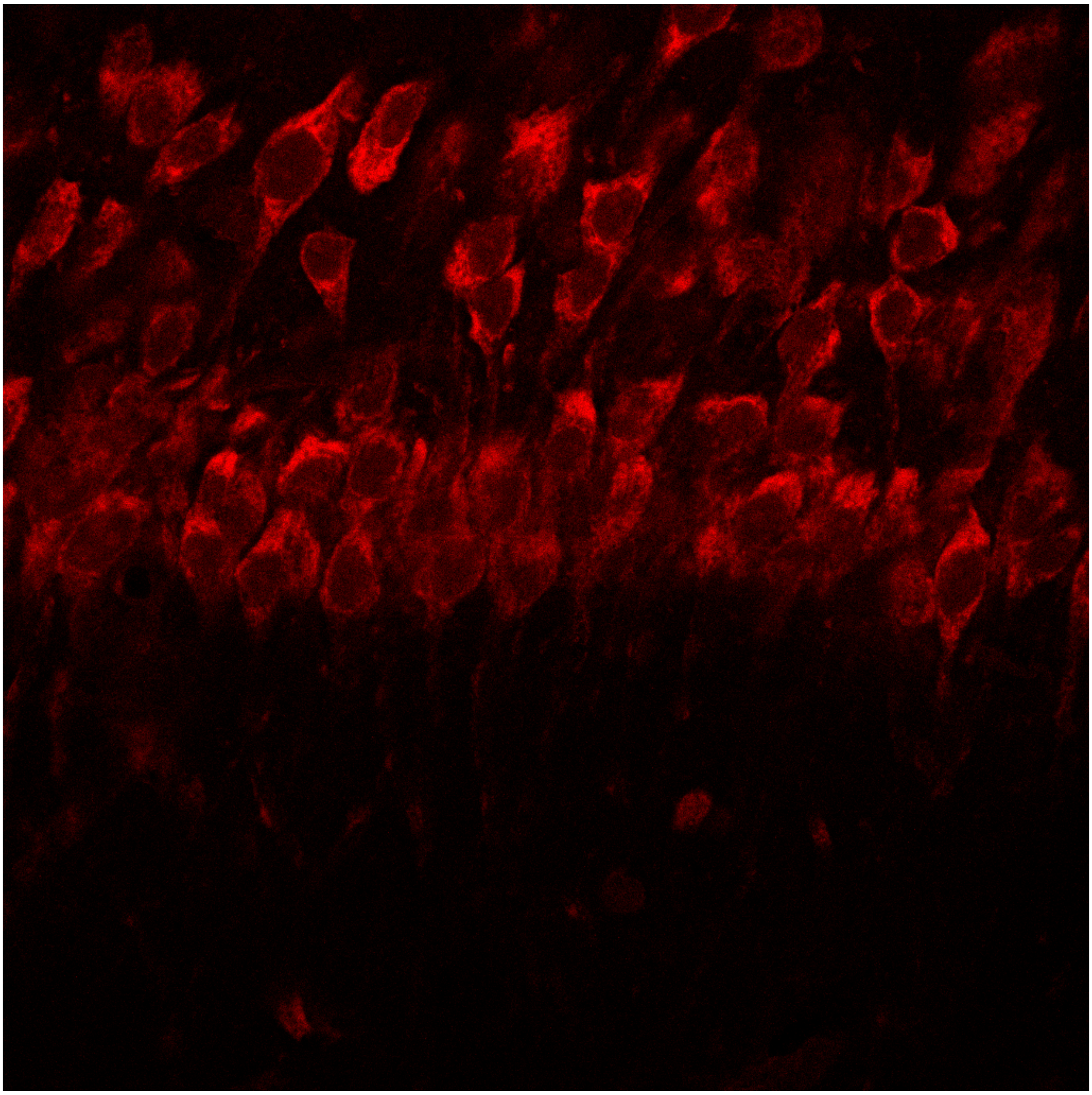

PVD3IpsilateralCA163Xshoot2

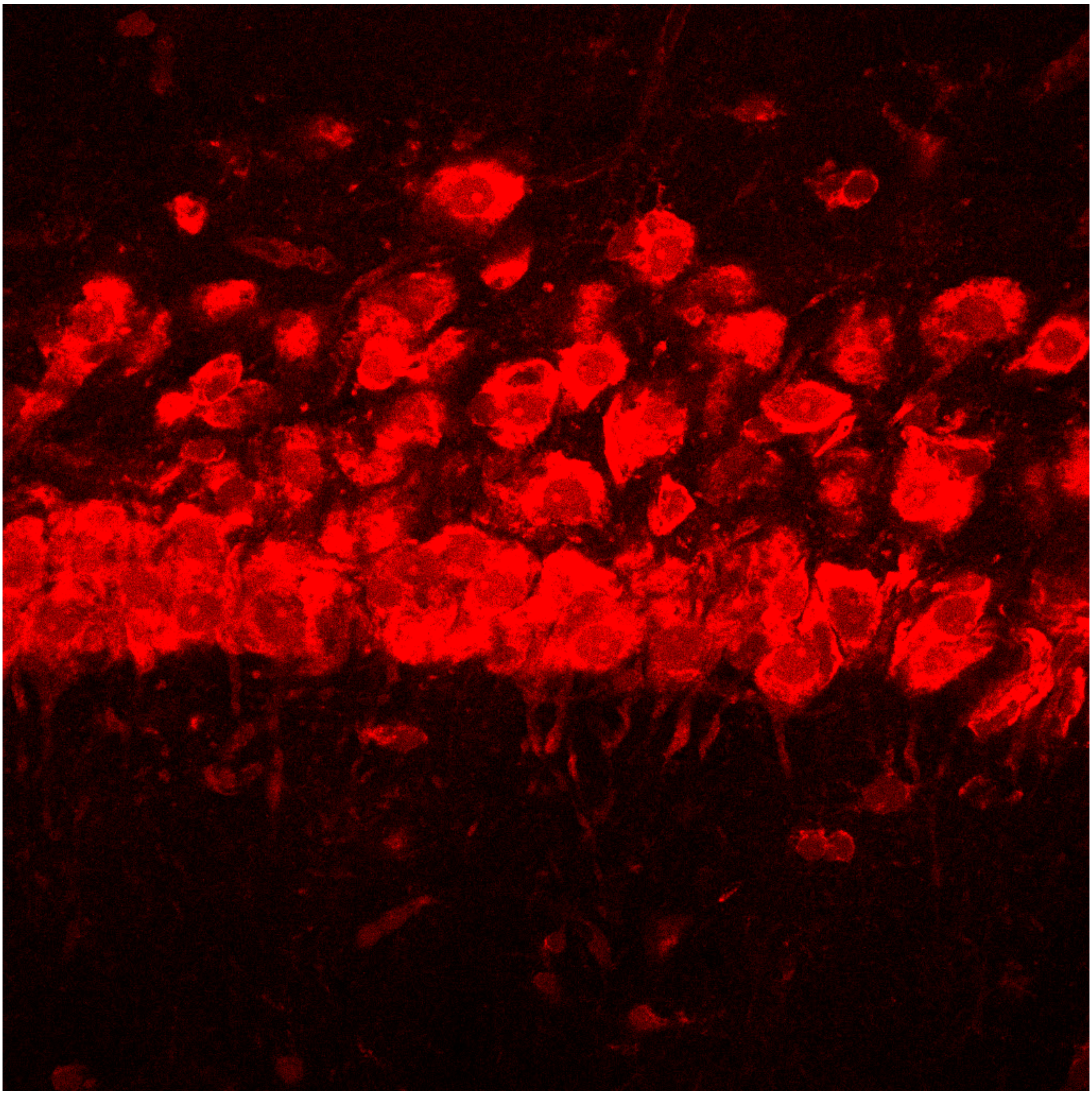

PVD3ContralateralCA163Xshoot1

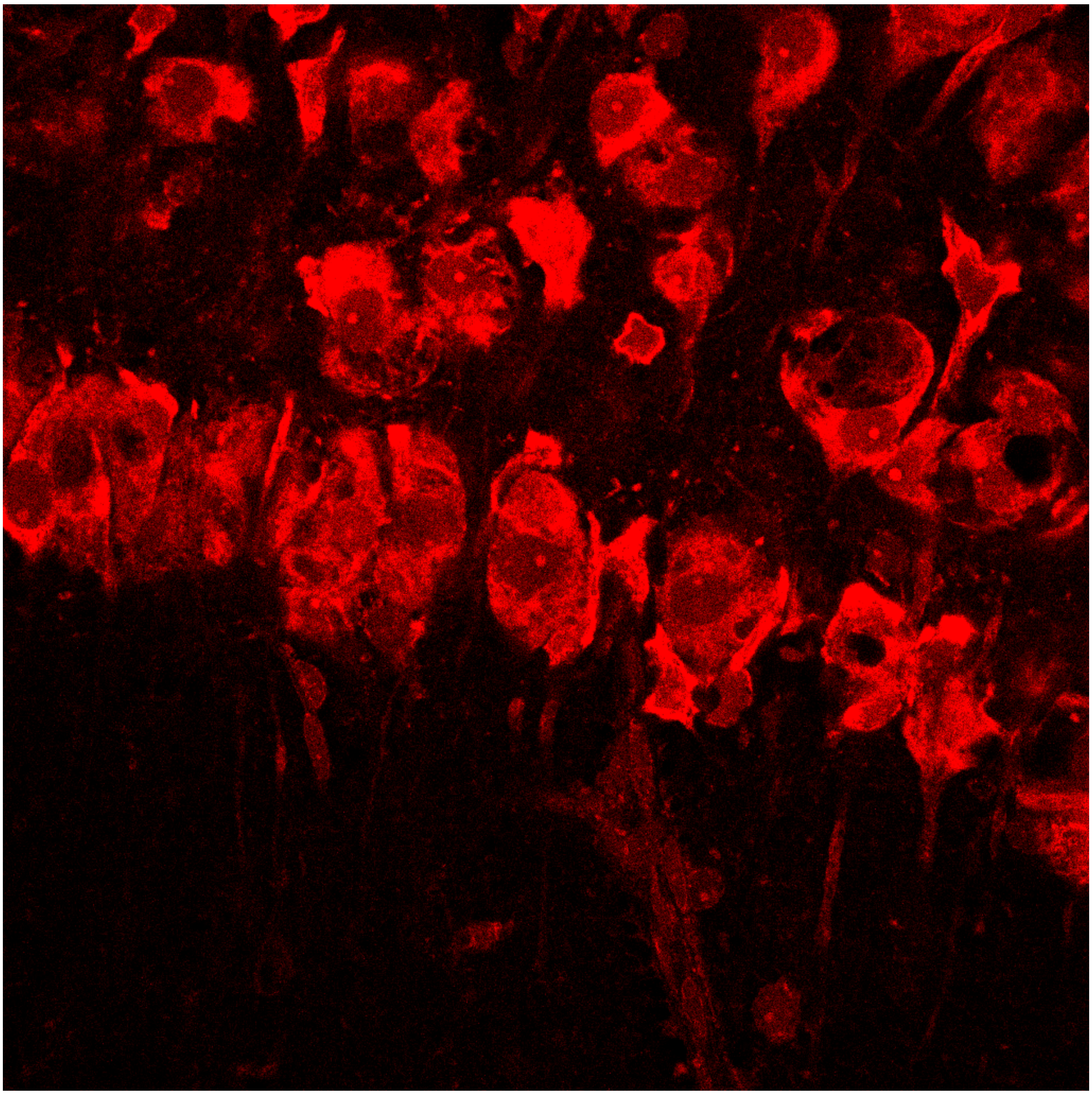

PVD2Istradefyllineipsilateral63Xshoot1

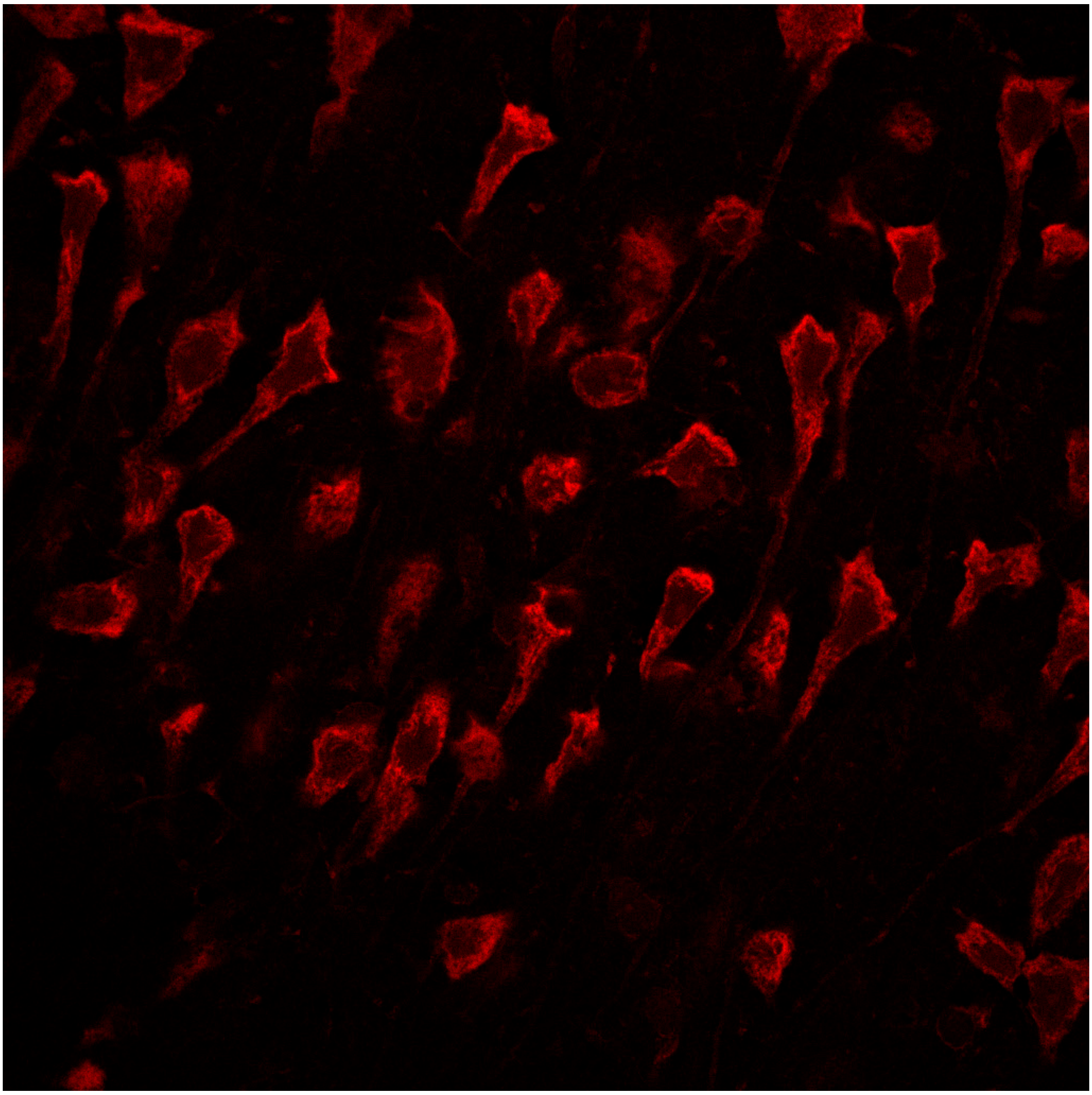

PVD2Istradefyllinecontrailateral63Xshoot1

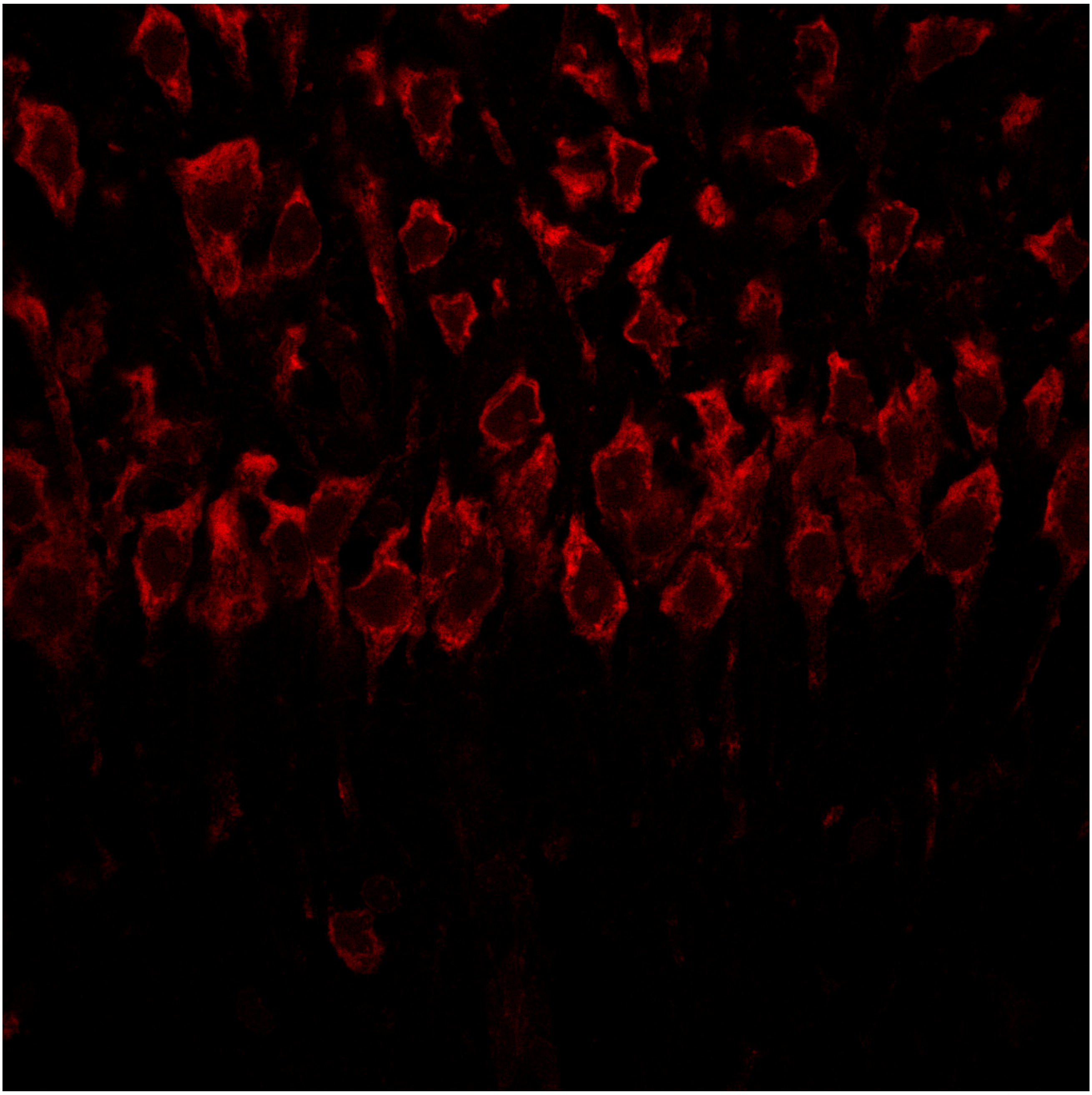

PVD Istradefylline ipsilateral 63 X

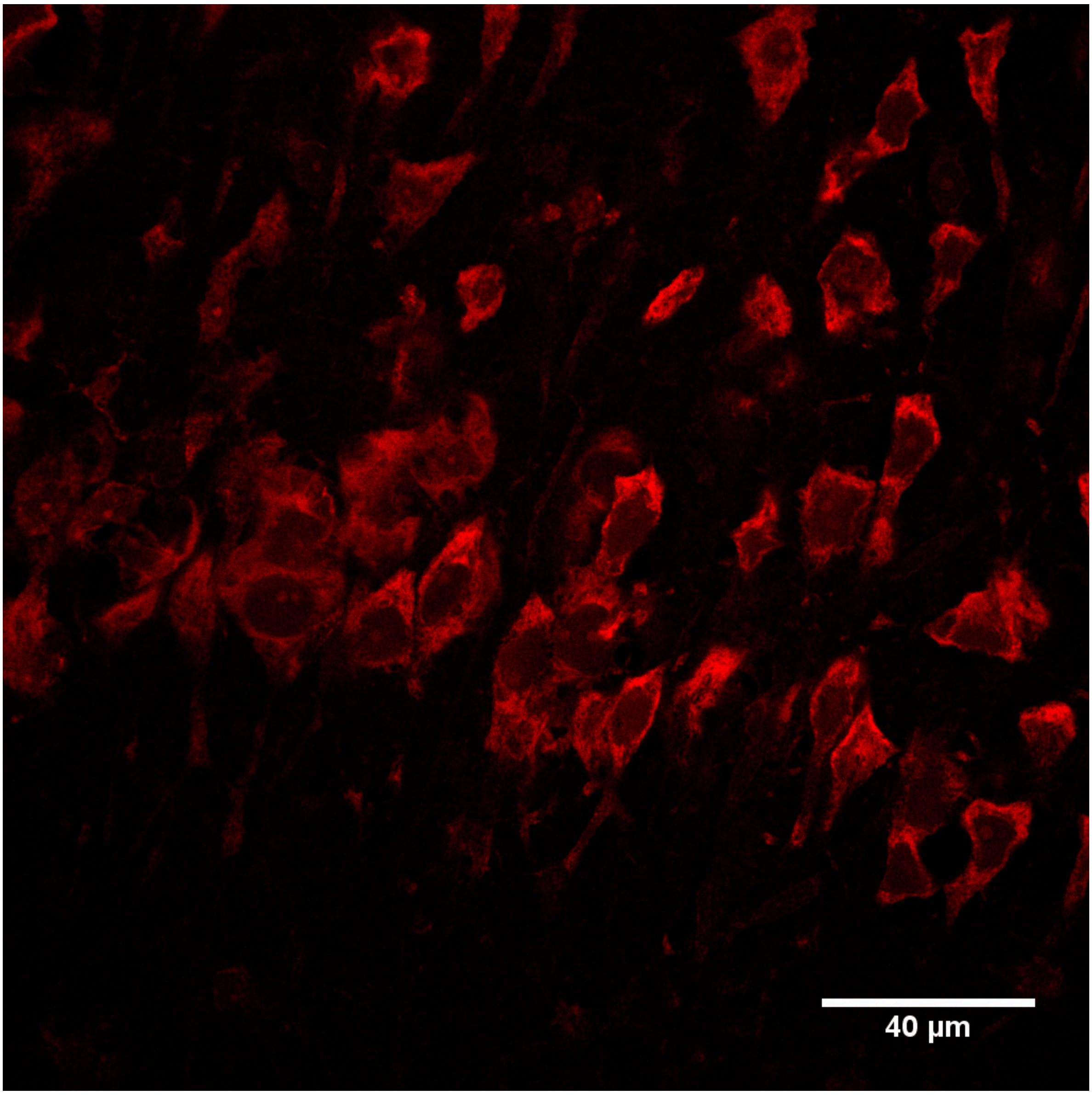

PVDIstradefylline contrailateral 63 X

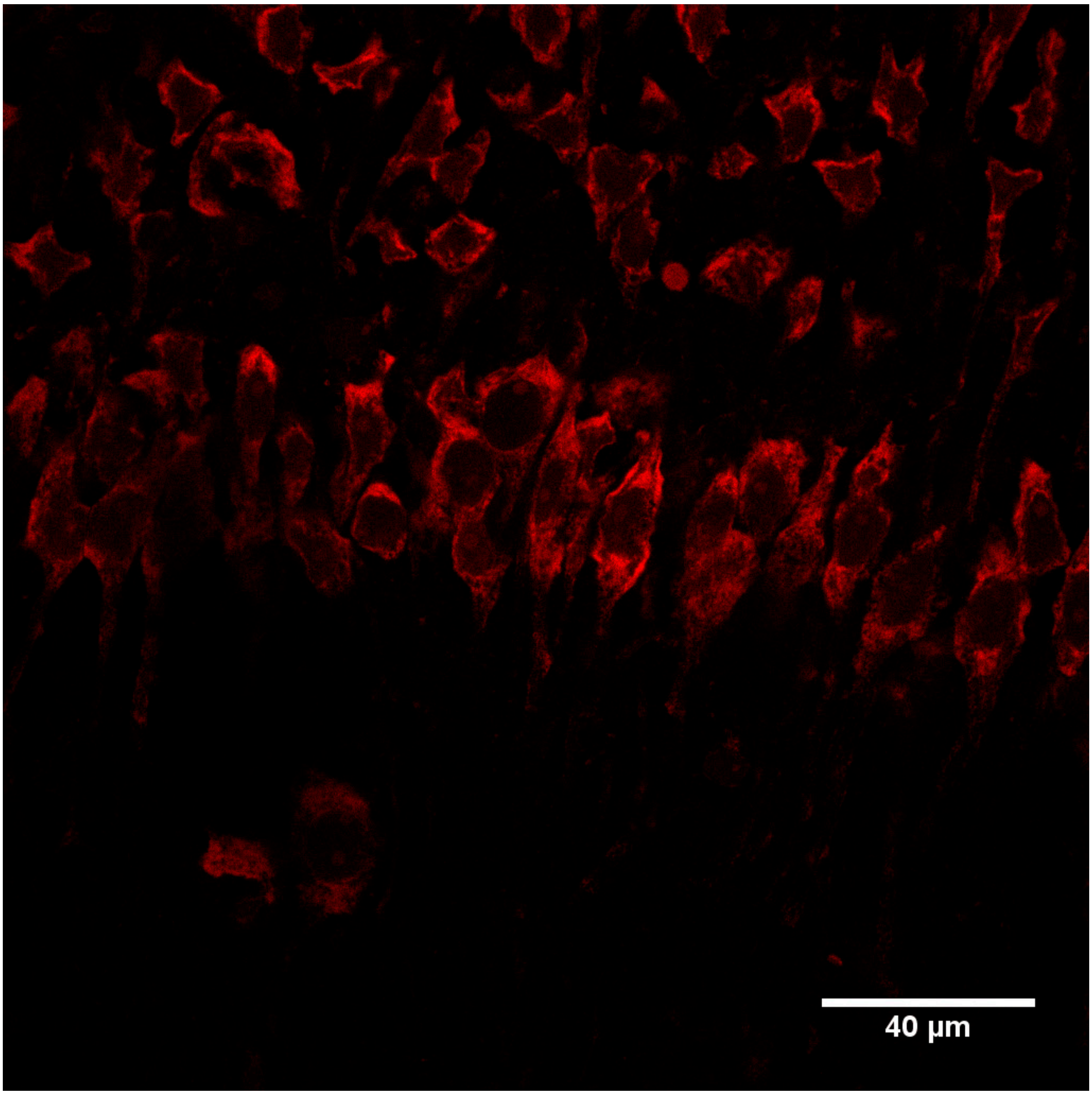

PVD1Istradefyllineipsilateral63Xshoot1

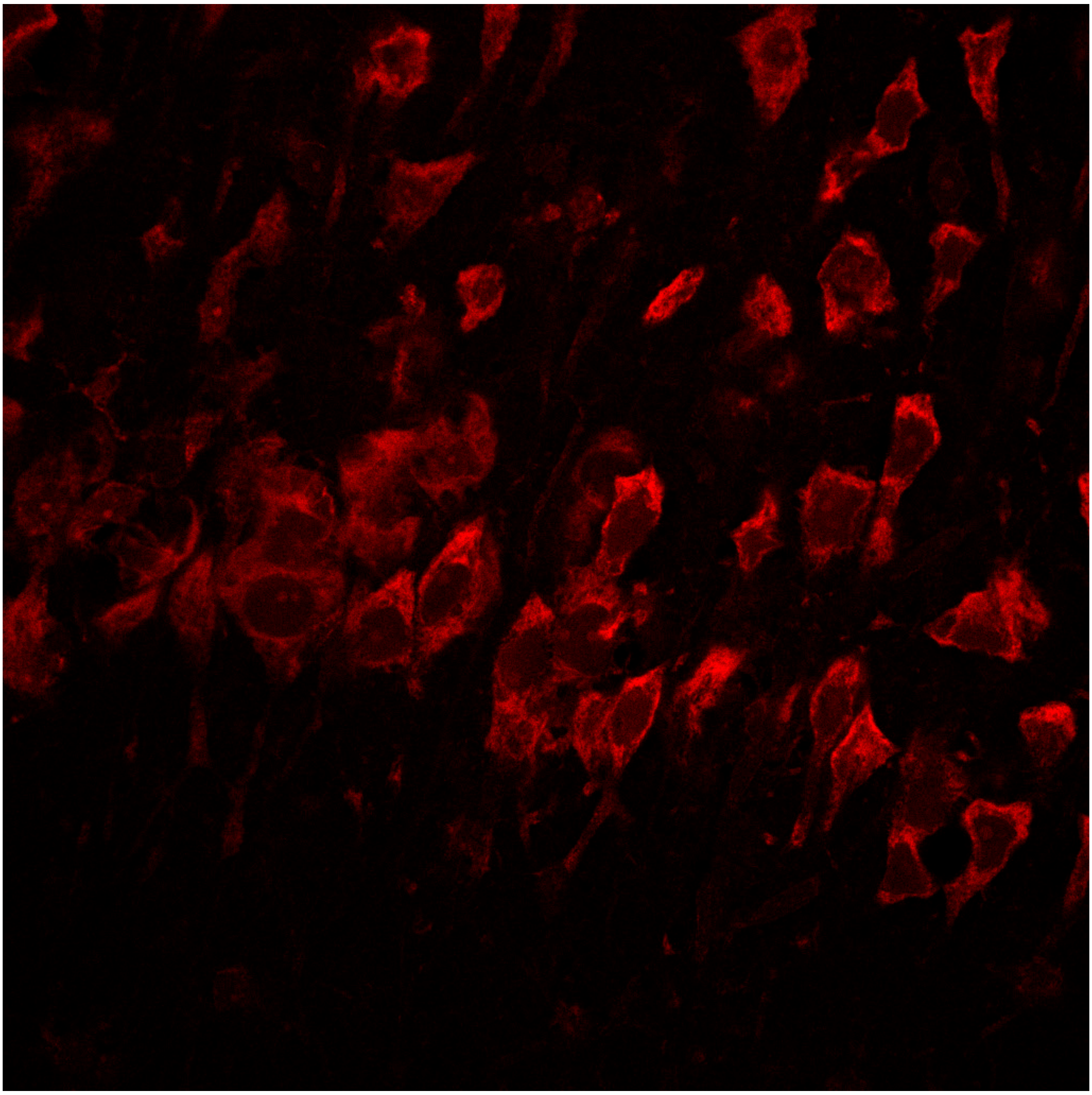

PVD1Istradefyllinecontrailateral63Xshoot1

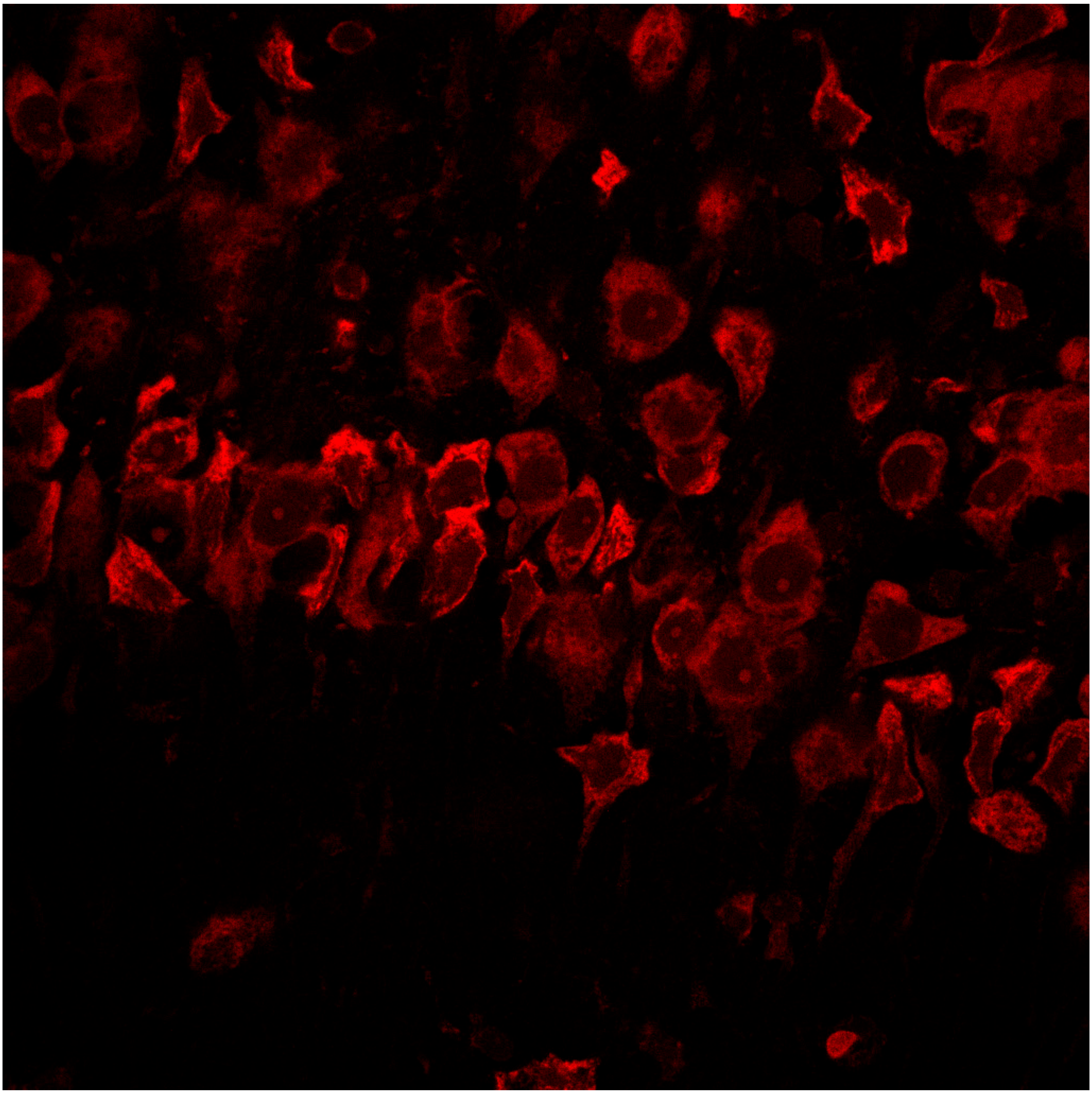

Supplement: Supplementary file 1 [file ijms-26-05680-s001.zip › FluoroJade C and Prop Iodide_June 4 2025.pdf]
