# Supplementary material for: The Anti-Parkinsonian A2A Receptor Antagonist Istradefylline (KW-6002) Attenuates Behavioral Abnormalities, Neuroinflammation, and Neurodegeneration in Cerebral Ischemia: An Adenosinergic Signaling Link Between Stroke and Parkinson’s Disease
Source: Int J Mol Sci. 2025 Jun 13;26(12):5680. doi: 10.3390/ijms26125680 (PMC12193193; doi:10.3390/ijms26125680)
Supplement: Supplementary file 1 [file ijms-26-05680-s001.zip › GFAP For ISTRA paper.pdf]

GFAP

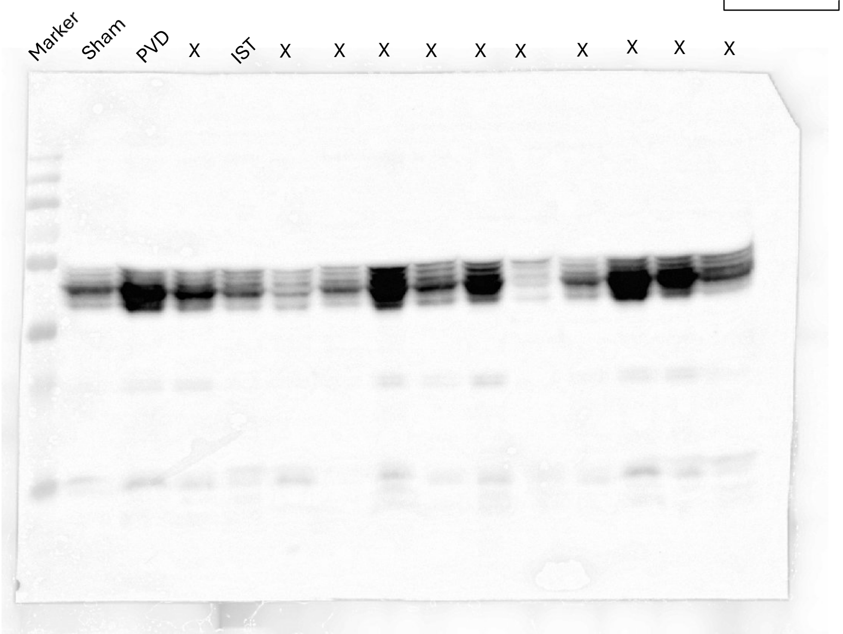
Marker
Sham
PVD
X
IST
X
X
X
X
X
X
X
X
X
X

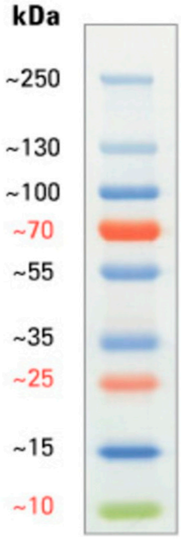
kDa
~250
~130
~100
~70
~55
~35
~25
~15
~10

B-actin

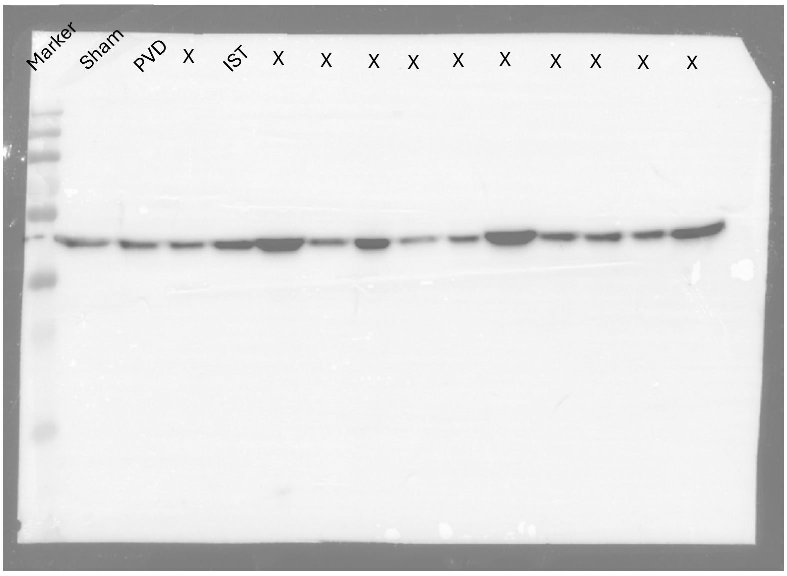
Marker
Sham
PVD
X
IST
X
X
X
X
X
X
X
X
X
X

GFAP

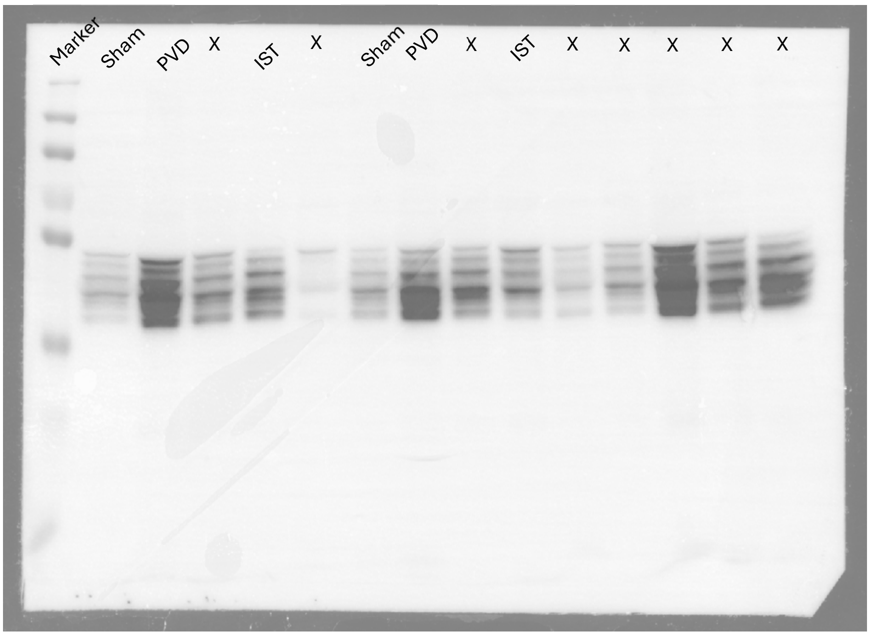
Marker
Sham
PVD
X
IST
X
Sham
PVD
X
IST
X
X
X
X
X

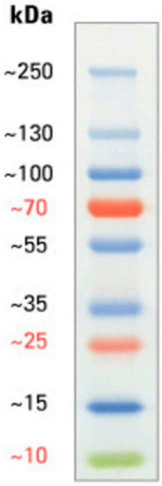
kDa
~250
~130
~100
~70
~55
~35
~25
~15
~10

B-actin

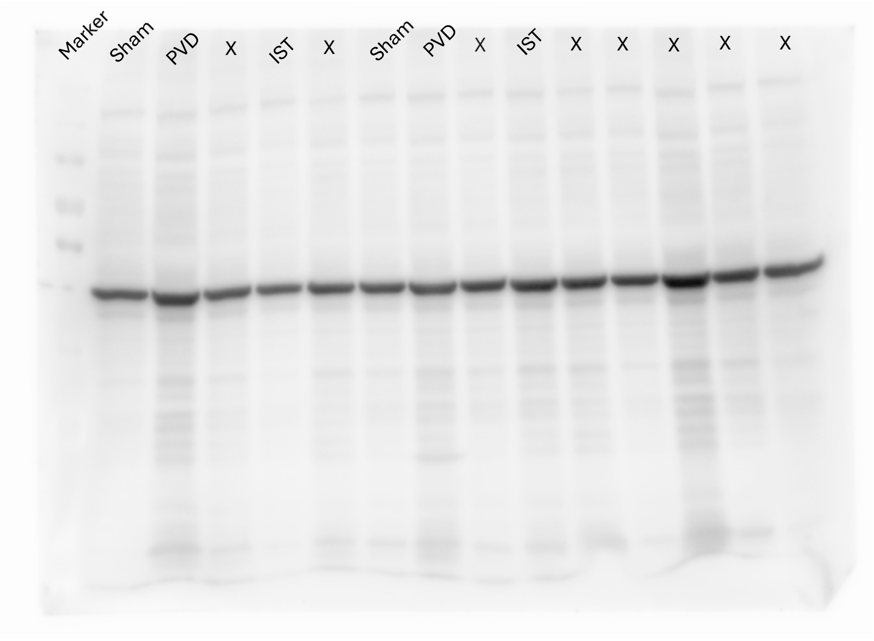
Marker
Sham
PVD
X
IST
X
Sham
PVD
X
IST
X
X
X
X
X

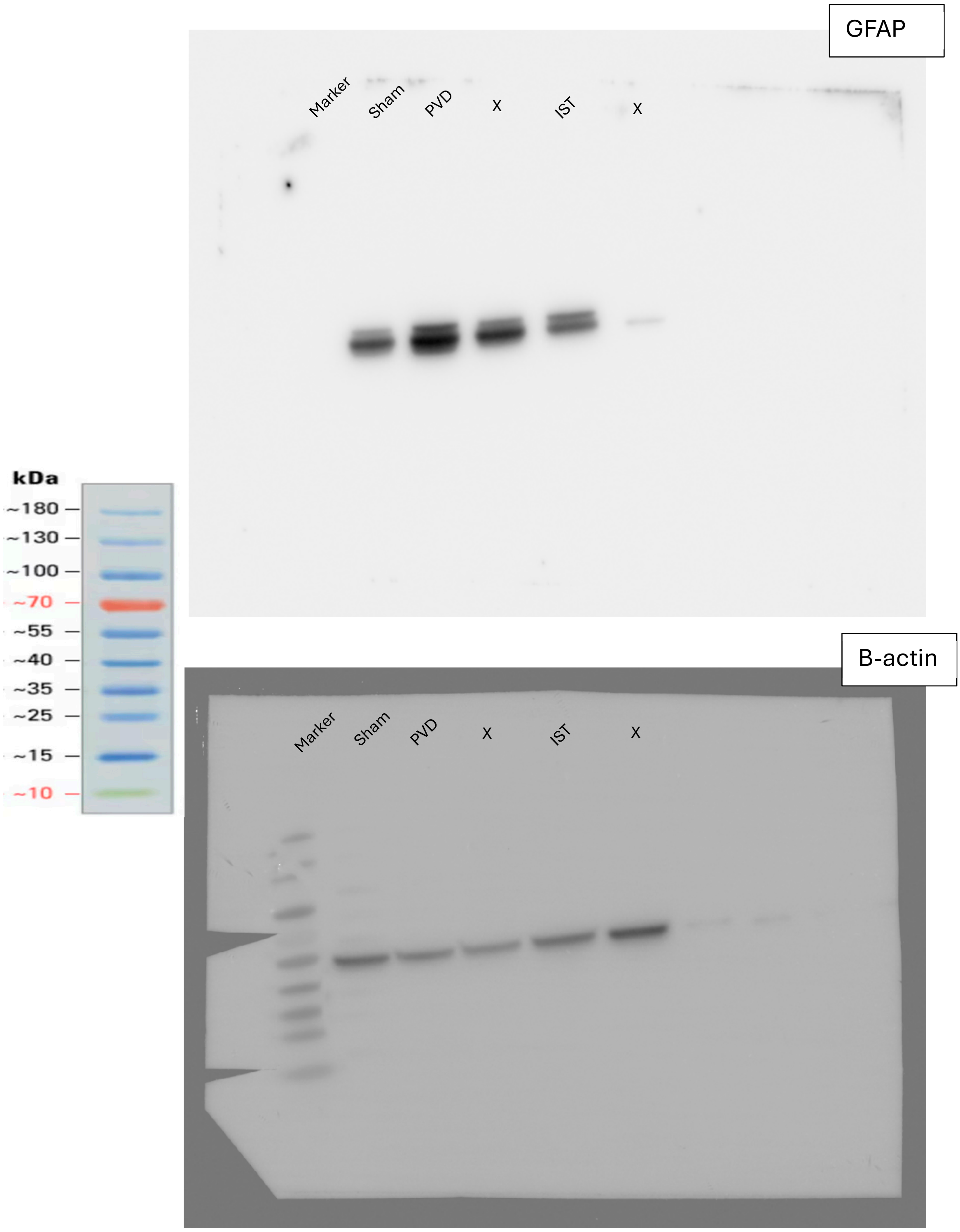
GFAP
Marker
Sham
PVD
X
IST
X
kDa
~180
~130
~100
~70
~55
~40
~35
~25
~15
~10
B-actin
Marker
Sham
PVD
X
IST
X

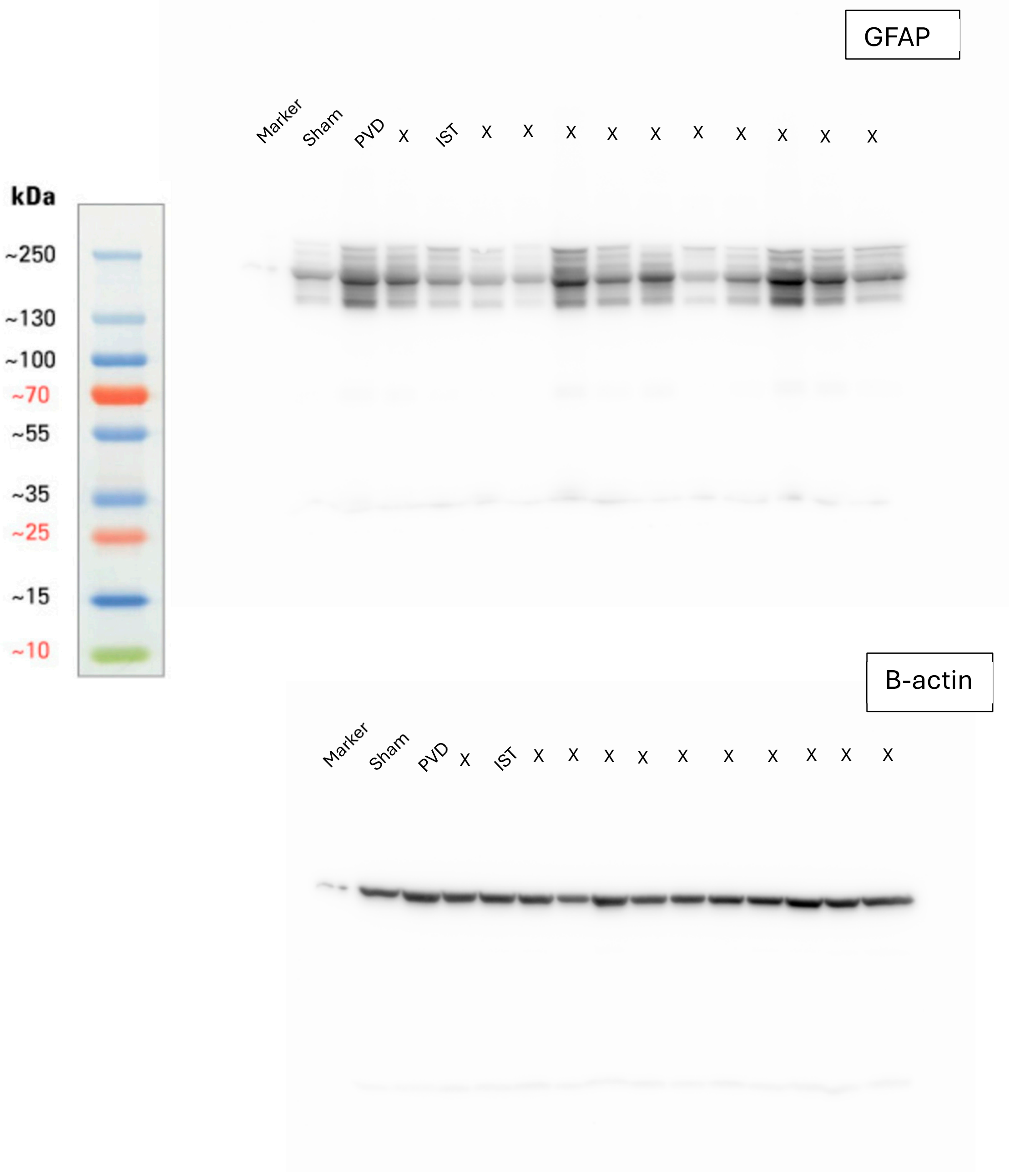
GFAP
Marker
Sham
PVD
X
IST
X
X
X
X
X
X
X
X
X
X
kDa
~250
~130
~100
~70
~55
~35
~25
~15
~10
B-actin
Marker
Sham
PVD
X
IST
X
X
X
X
X
X
X
X
X
X

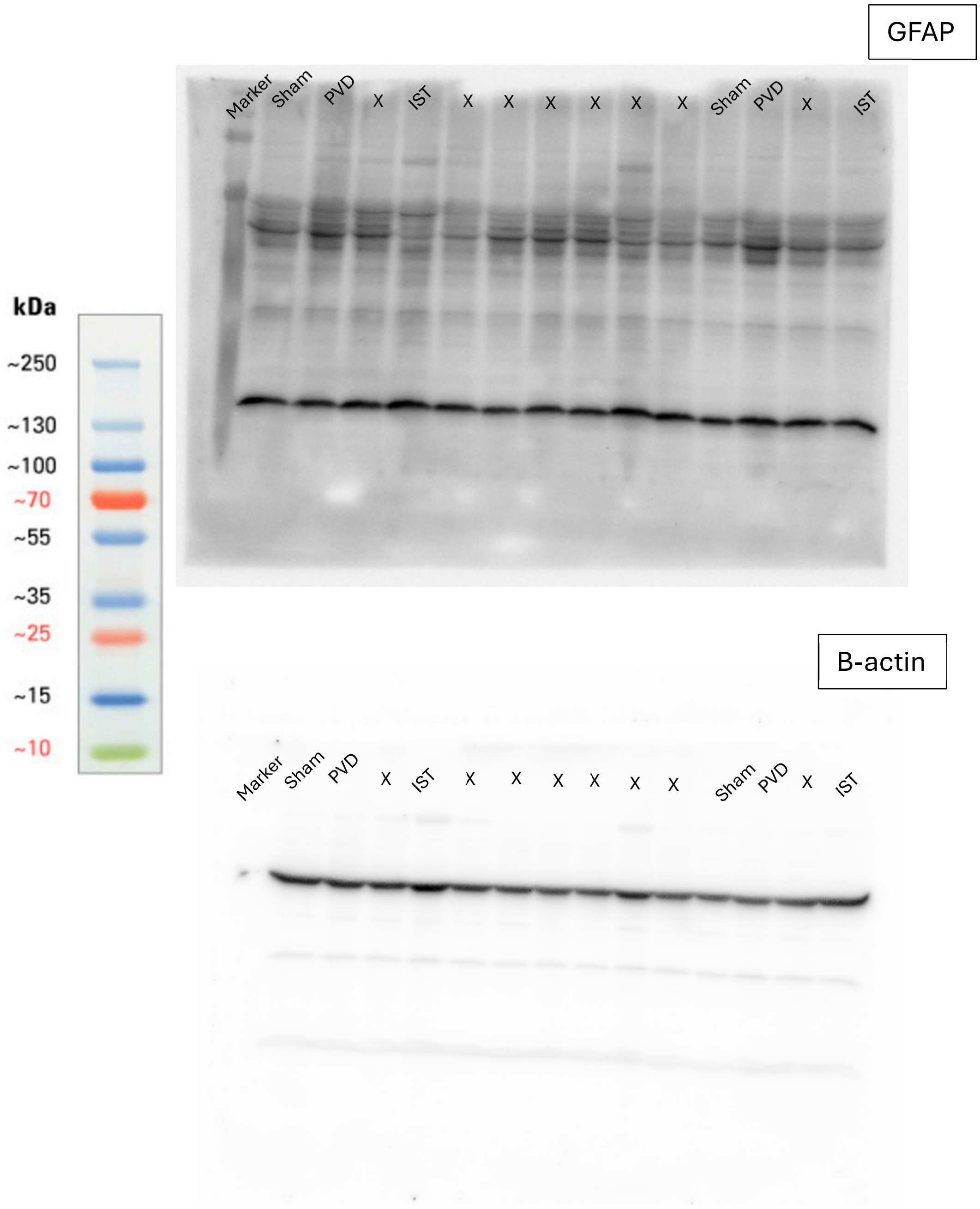
GFAP
Marker Sham PVD X IST X X X X X X Sham PVD X IST
kDa
~250
~130
~100
~70
~55
~35
~25
~15
~10
B-actin
Marker Sham PVD X IST X X X X X X Sham PVD X IST
