# Supplementary figures and images for: The Anti-Parkinsonian A2A Receptor Antagonist Istradefylline (KW-6002) Attenuates Behavioral Abnormalities, Neuroinflammation, and Neurodegeneration in Cerebral Ischemia: An Adenosinergic Signaling Link Between Stroke and Parkinson’s Disease
Source: Int J Mol Sci. 2025 Jun 13;26(12):5680. doi: 10.3390/ijms26125680 (PMC12193193; doi:10.3390/ijms26125680)

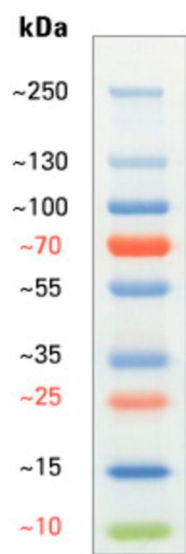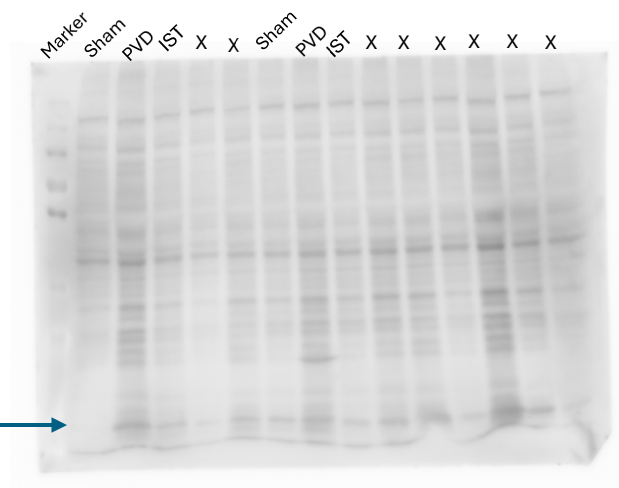

IBA1

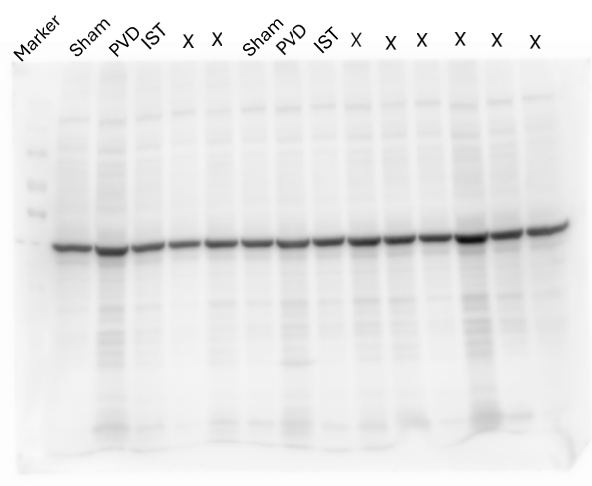

B-actin



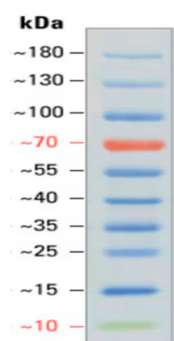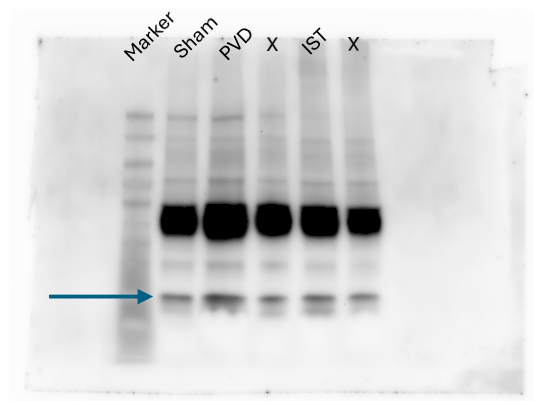

IBA1

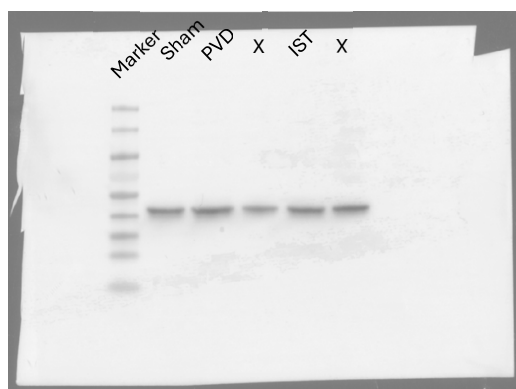

B-actin

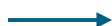

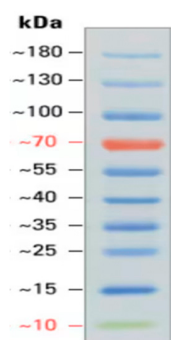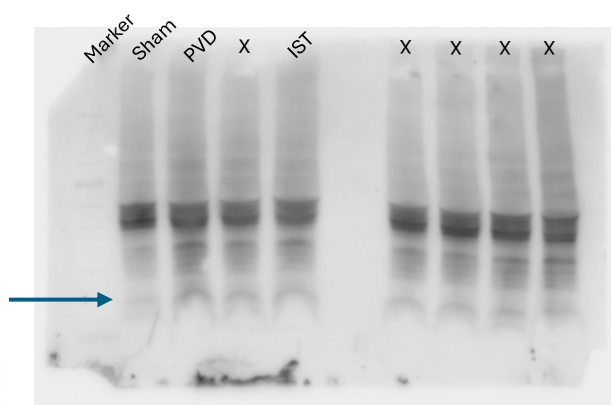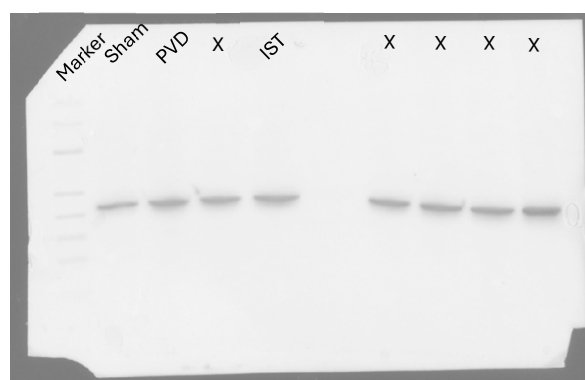

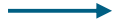

IBA1

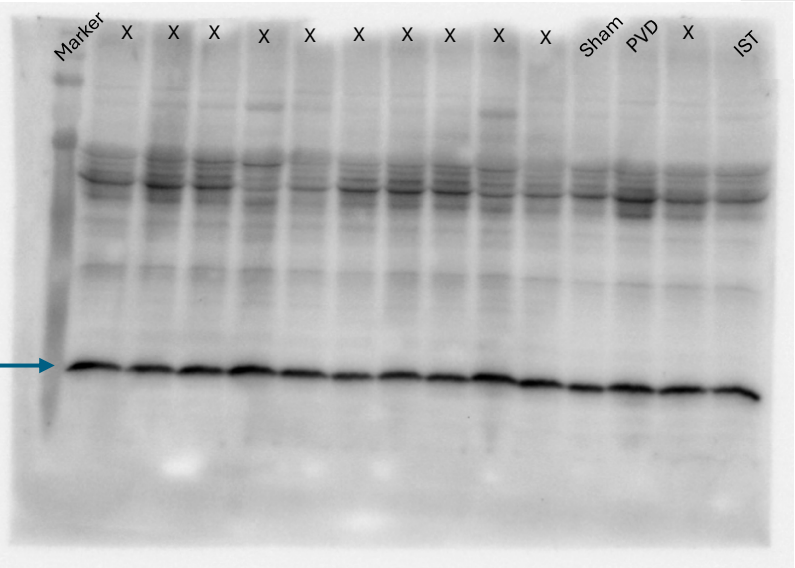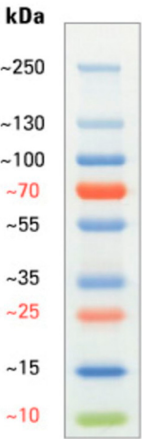

B-actin

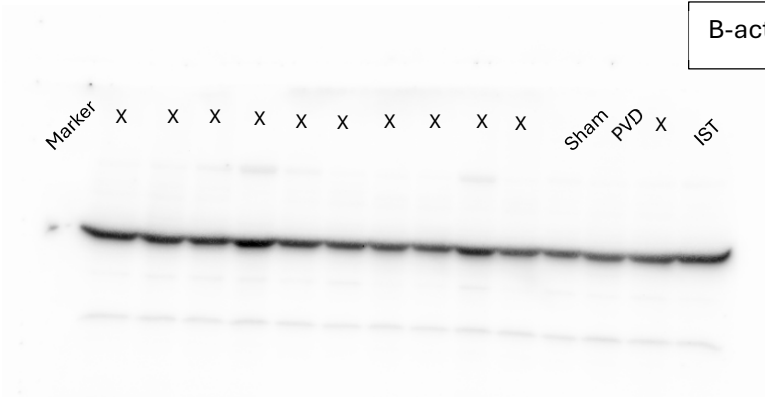

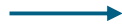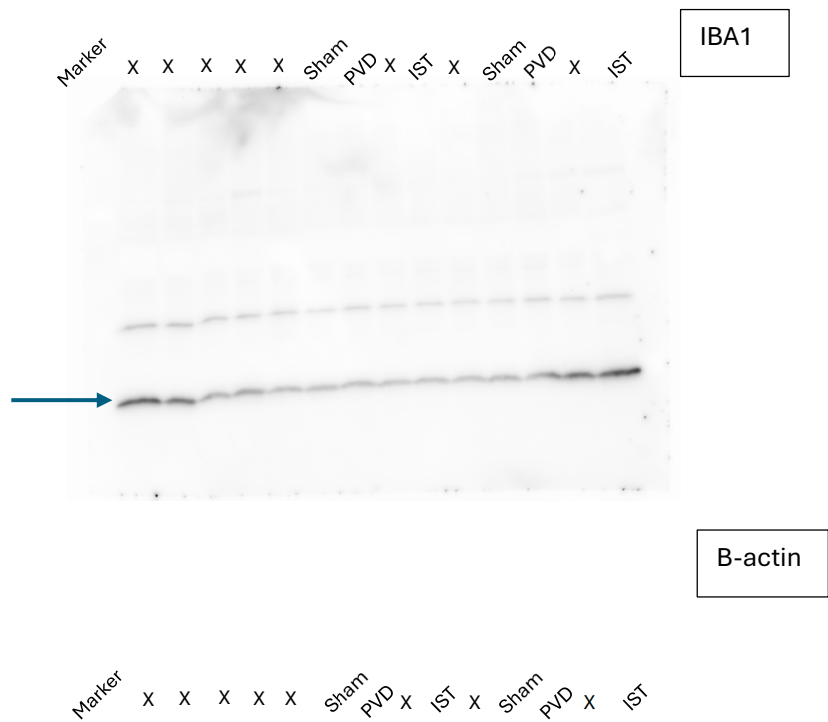

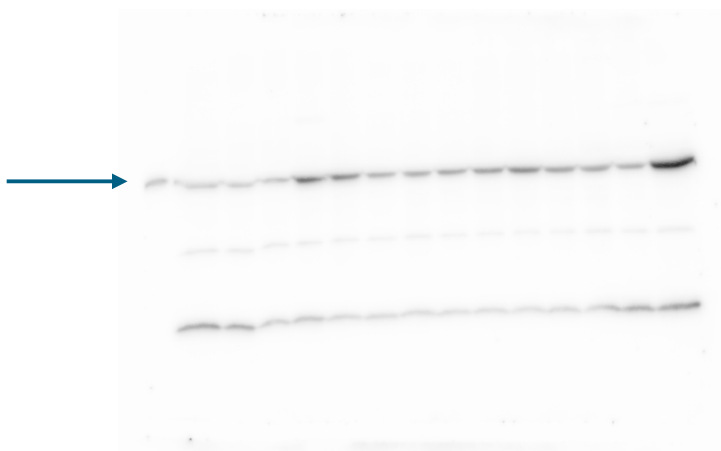

Supplement: Supplementary file 1 [file ijms-26-05680-s001.zip › IBA1 For ISTR paper.pdf]

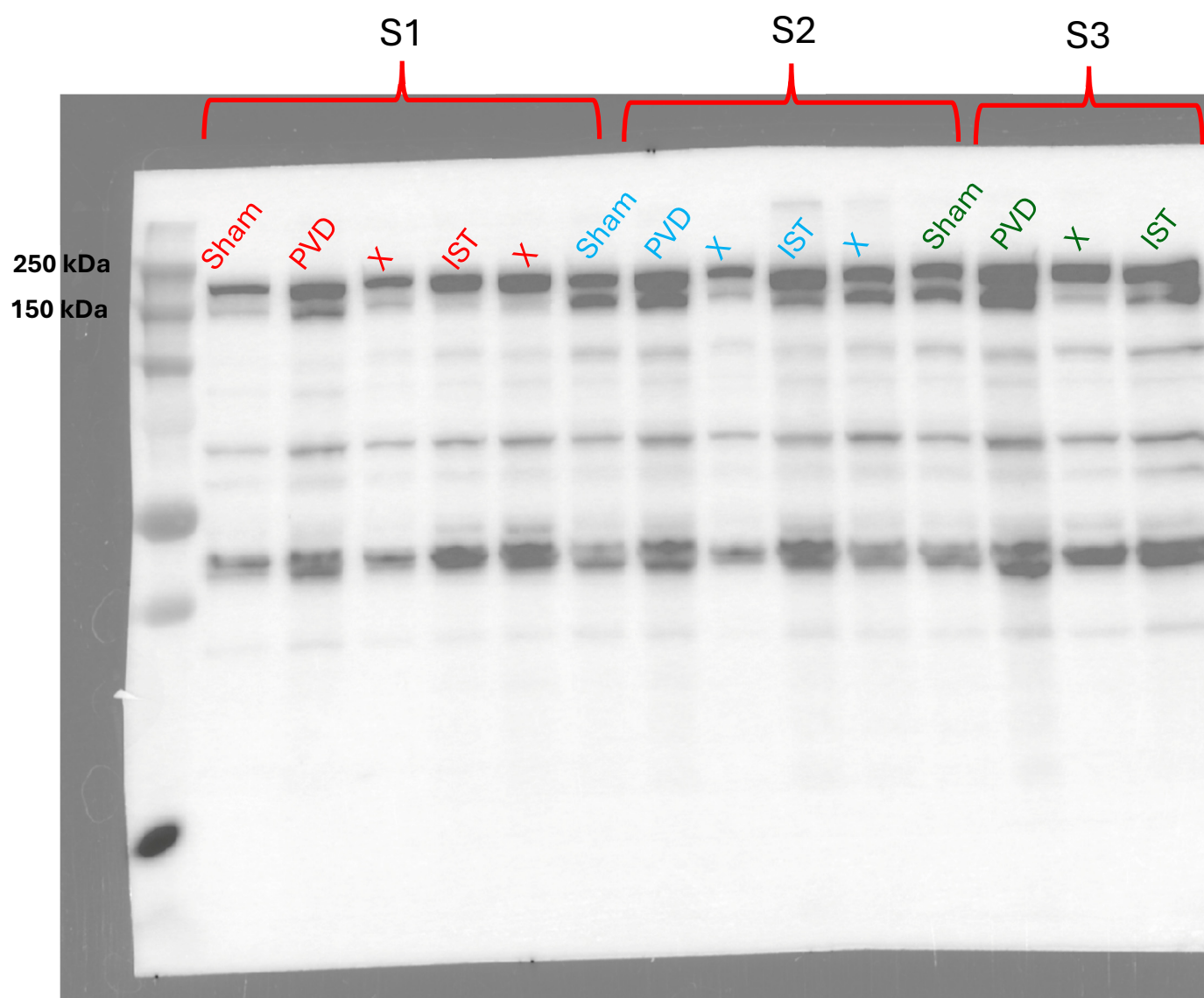

Supplement: Supplementary file 1 [file ijms-26-05680-s001.zip › nNOS original blots.pdf]
